# Strategies to Promote Resiliency: A Randomized Embedded Multifactorial Adaptative Platform (REMAP) Clinical Trial to Study Interventions to Improve Recovery After Surgery in High-Risk Patients

**DOI:** 10.1097/AS9.0000000000000566

**Published:** 2025-04-02

**Authors:** Katherine M. Reitz, Hasan Nassereldine, Jason Kennedy, Ryan Zeh, Farah Khandwala, Christopher W. Seymour, Melanie Quintana, Kert Viele, Michelle Detry, Alison Morris, Barbara Methe, Brian Zuckerbraun, Timothy D. Girard, Oscar C. Marroquin, Stephen Esper, Jennifer Holder-Murray, Anne B. Newman, Timothy R. Billiar, Scott Berry, Derek C. Angus, Matthew D. Neal

**Affiliations:** From the * Department of Surgery, University of Pittsburgh, Pittsburgh, PA; † Division of Vascular Surgery, University of Pittsburgh, Pittsburgh, PA; ‡ Department of Vascular Surgery, Veterans Affairs Pittsburgh Healthcare System, Pittsburgh, PA; § Department of Critical Care Medicine, UPMC, Pittsburgh, PA; ‖ Berry Consultants Statistical Innovation, Austin, TX; ¶ Department of Medicine, UPMC, Pittsburgh, PA; # Clinical Analytics, UPMC Health System, Pittsburgh, PA; ** Department of Anesthesiology, University of Pittsburgh, Pittsburgh, PA; †† Department of Epidemiology, University of Pittsburgh, Pittsburgh, PA; ‡‡ UPMC Presbyterian, Pittsburgh, PA.

**Keywords:** clinical trials, elective surgery, frailty, information management, metformin

## Abstract

**Objective:**

In a randomized, embedded, multifactorial, adaptive platform (REMAP) trial, we hypothesized that perioperative metformin would improve postoperative time alive and out of the hospital, defined by 90-day hospital-free days (HFD-90), among nondiabetic aged adults.

**Background:**

As our population ages, patients are increasingly frail requiring an emphasis on treatments to counteract their diminished resilience, especially following the stress of surgery. Growing literature supports metformin as an antiaging and anti-inflammatory therapy with beneficial effects extending into the perioperative period.

**Methods:**

At-risk adults (≥60 years) scheduled for elective surgical interventions were randomized to placebo (N = √3) or metformin (N = 1:1:1; 500 mg:1000 mg:1500 mg) for short (7–28 days), intermediate (29–90 days), or long (>90 days) preoperative durations. An adaptive sample size of (N = 1000–2500) would identify at least a 15% improvement in HFD-90 for >1 metformin doses. Using intention-to-treat analysis, Bayesian ordinal logistic regression compared HFD-90 and frequentist logistic regression compared 90-day reoperation and readmission.

**Results:**

Before trial closure, we randomized 302 (N = 106 placebo, N = 196 metformin [N = 64, 500 mg; N = 66, 1000 mg; and N = 66, 1500 mg]) patients without differences in baseline demographics (age 68 ± 6 years, 45% females, and 92% White race) or interventions (spine [29%], general [38%], colorectal [13%], and other [20%]). The odds of HFD-90 did not significantly differ between all doses and duration of metformin or placebo. There were no differences in the odds of reintervention (OR = 1.1 [95% CI, 0.6–2.0]) or readmission (OR = 1.5 [95% CI, 0.7–2.8]).

**Conclusions:**

Pretreatment with metformin did not improve postoperative outcomes in this REMAP trial, although trial enrollment was markedly limited by the COVID-19 pandemic and is underpowered.

## INTRODUCTION

Elderly individuals, who are more frequently frail, account for more than one-third of all surgical procedures and, therefore, face a higher risk of postoperative complications and death.^1^ The culmination of medical, social, and functional challenges define frailty rendering individuals increasingly susceptible to physiological stress, diminishing their resilience and reserve.^2,3^ In addition, a single major stressor, such as a surgical intervention, provides an accelerated and temporally specific reduction in resiliency.^4^ Prehabilitation before surgery has been shown to improve outcomes,^5^ and the aging population undergoing surgery represents a vulnerable cohort for whom the benefits of prehabilitation may be even further enhanced.

A wide array of comprehensive screening and treatments, including methods such as smoking cessation, improved nutrition, and exercise programs are being explored to prevent and mitigate the impact of aging and the resulting diminished physiological reserve.^6,7^ Such efforts have expanded to include antiaging pharmacotherapies aimed at reducing the adverse effects of aging. Aging is a continuous and gradual inflammatory process that leads to tissue dysfunction and a decline in physiological function, ultimately contributing to the development of age-related diseases such as neurodegenerative disorders, cardiovascular conditions, and diabetes, thus increasing frailty.^8^ One such therapy is metformin. Beyond its role in glycemic control and use for diabetes, metformin has an excellent safety profile with ever-expanding support of its anti-inflammatory and antiaging properties among diabetic and nondiabetic patients.^9–11^ We have previously shown through retrospective analysis that exposure to metformin was an independent predictor of improved mortality following major surgery.^11^

Based upon our prior findings with metformin in major surgery and the need for enhanced prehabilitation in older patients, we developed a randomized, embedded, multifactorial, adaptive platform (REMAP)^12,13^ trial to assess the impact of perioperative metformin compared with placebo across a network of hospitals within a single healthcare system: Strategies to Promote ResiliencY (SPRY). We hypothesized that perioperative metformin would improve postoperative time alive and at home as measured by 90-day hospital-free days (HFD-90) when compared with placebo among aged, nondiabetic adults.

## METHODS

### Trial Design

The SPRY REMAP trial design aligns with the recommendations of the Adaptive Platform Trials Coalition,^14^ SPIRIT guidelines,^15^ CONSORT guidelines (Supplemental File CONSORT statement, see http://links.lww.com/AOSO/A487), and the University of Pittsburgh Human Research Protection Office (STUDY20040236). The trial is registered in clinicaltrials.gov (NCT03861767).

#### SPRY Core Protocol

SPRY is the first core protocol describing the inclusion of a trial within the electronic health record (EHR) and routine perioperative healthcare delivery for older at-risk patients. SPRY was designed with the intent to evaluate several domains concurrently using Bayesian statistical analysis and response adaptive randomization measuring the treatment effect in prespecified strata (ie, colorectal, general, and orthopedic surgical interventions). In the REMAP design, patients have the possibility of being randomly assigned to one of several treatments across a range of concurrently active domains.

#### SPRY-Metformin Domain-Specific Appendix

SPRY-Metformin was a single, multihospital, healthcare system, placebo-controlled, adaptive, phase III clinical trial, blinded at the level of the patient, clinician, research team, and data analyst. Prespecified adaptive randomization strategies for dose and duration dropping as well as thresholds for treatment efficacy, inferiority, futility, and equivalence were defined for interval analysis with 500 patients enrolled and for every additional 500 patients up to 2500 followed for 90 days. The trial was integrated into the EHR and clinical workflow through a custom software, the SPRY-application, which automated the initial process of screening, enrollment, and randomization. Furthermore, the data abstraction process mirrored the methodology used usually for retrospective EHR data acquisition and analysis, however, with simultaneous real-time data updates. The complete description of the trial design and trial embedding was previously published.^13^

### Participants

All patients were recruited from a multihospital single provider and insurer healthcare system serving Southwestern Pennsylvania and the surrounding states. The embedded application reviewed the EHR for all adults (age > 18) scheduled for outpatient surgical clinic and preoperative anesthesia evaluations. Application included all adults with a potentially increased risk of postoperative morbidity and mortality including increased age (≥60 years) or numerous comorbid conditions (Charlson Comorbidity Index > 2). The application excluded all patients with an EHR diagnosis of diabetes. Identified patients were then approached at in-person or e-visits for their interest in participation and confirmation they had a planned major, elective surgical intervention and met all inclusion as well as no exclusion criteria (Supplemental Table 1, see http://links.lww.com/AOSO/A487). Major surgical interventions were defined by the need for general anesthesia. Trial screening began on April 15, 2019, and concluded on April 28, 2022. The University of Pittsburgh Human Research Protection Office mandated screening and enrollment pauses (March 20, 2020–August 17, 2020) and UPMC followed national guidelines limiting elective surgical interventions in response to the COVID-19 pandemic.

### Study Drug and Randomization

Patients meeting all inclusion and exclusion criteria were then randomized √3:1:1:1 to placebo and 3 doses of daily metformin ER (500, 1000, or 1500 mg). Randomization was stratified by enrollment clinic, age category (<60 with Charlson Comorbidity Index >2 or ≥60 years), and expected preoperative study-drug duration. Pragmatically, dependent on the intended window between enrollment and their elective operation, patients were stratified into 3 preoperative study-drug durations (short [7–28 days], medium [29–90 days], and long [>90 days]). Study drug exposure continued through 90 postoperative days without any planned perioperative therapy interruptions. Considering historical practice suggesting cessation of metformin perioperatively, we embedded EHR safety alerts. The embedded trial automatically monitored resultant hospitalization laboratory results, highlighting potential hepatic or renal dysfunction, or an order for intravenous contrast agents. If appropriate, an EHR alert informed nursing to hold study drug until providers could review its receipt.^13^

Study drug adherence was defined by verbal confirmation of study drug completion at the 90-day follow-up timepoint. Trial withdrawal was defined by verbal confirmation from the participant concerning withdrawing consent from the study.

### Outcomes

The primary endpoint was the ordered categorical variable HFD-90. HFD-90 is a patient-centered outcome summing the postoperative days alive and out of the hospital.^16^ The score is distributed as follows: −1 was attributed patients that die intraoperatively or within 90 days postoperatively, 0 represents patients who were alive but hospitalized for the entire 90-day postoperative period, and 1 to 90 cumulates the postoperative days alive and out of the hospital. The calculation of HFD-90 was embedded and summed in real-time through EHR data generated from clinical practice. Index admission secondary outcomes included postoperative intensive care unit admission, intensive care unit length of stay, hospital length of stay, discharge disposition, and inhospital mortality. Thirty-day outcomes included organ failure-free days, surgical site infection, and surgical site occurrence defined as by the ventral hernia working group as any surgical site complication, including infection, seroma, wound dehiscence, and the formation of enterocutaneous fistulae. 90-day outcomes included reintervention, readmission, venous thromboembolic events, and mortality. Adverse events were monitored throughout the perioperative periods.

Due to the early termination of the trial and termination of funding, the secondary endpoints planned for 365 days of monitoring were only assessed up to postoperative day 90. In addition, the longitudinal quality of life and frailty secondary endpoints initially planned for assessment on day 30 and day 90 postoperatively were not evaluated.

### Sample Size

Power calculations for a Bayesian adaptive trial estimated the average power and trial efficiency of different sample sizes through simulation (statistical analysis plan [SAP], Supplemental File 2, see http://links.lww.com/AOSO/A487).^13,17^ Retrospective institutional EHR data created a virtual patient dataset, reflecting the HFD-90 distributions within each surgical stratum. Then, numerous simulated clinical trials were conducted, with patients randomly assigned to the study drug or placebo, incorporating interim analyses and prespecified adapted randomization. For example, assuming an expected maximal study drug treatment effect of 15% reduction in HFD-90 across all strata a maximum of 2000 to 2500 patients would provide >84% power to declare best metformin dose superiority over placebo if that dose was best across all durations. However, if the best dose was not effective in the short duration, the power to declare superiority is 77%. The summation of the simulations informed the adaptive randomization with a maximal sample size of 2500 patients and interim analysis and adaptive randomization at that time to occur for each 500 patients enrolled. The trial was terminated for enrollment futility on April 28, 2022, before the first interim analysis.

### Statistical Analysis

All analyses were prespecified (SAP, Supplemental File 2, see http://links.lww.com/AOSO/A487). Patients were analyzed through intention to treat (ITT) and per-protocol analysis (PPA). The ITT cohort consists of all patients enrolled and randomized to any treatment dose and duration who underwent a surgical intervention. The PPA cohort consists of all patients enrolled and randomized who underwent a surgical intervention, did not withdraw from the trial, and were adherent to their randomized study drug allocation. The primary cohort was ITT, but all analyses were recapitulated in the PPA cohort.

Summary statistics and error terms described demographic, baseline, and operative characteristics. Ordinal variables will be summarized by the number and cumulative frequency, categorical variables will be summarized by the proportion in each category, and continuous variables will be summarized by the mean, standard deviation or median, and interquartile range pending the distribution of the data.

The primary analysis of HFD-90 was conducted using Bayesian ordinal logistic regression permitting borrowing of information across different doses and durations on the treatment effect of metformin presented as median proportional odds ratio (mpOR) with 95% credible interval (95% CrI). An mpOR >1 indicates a worse outcome, whereas a higher mpOR suggests a higher odds of fewer HFD-90. Secondary outcomes will be analyzed through frequentist approach using univariable linear, logistic, and Cox regression where appropriate presented as beta-coefficients, odds ratios (OR), and hazard ratios (HRs), respectively, each with 95% confidence intervals (CIs). An OR and HR >1 indicates a higher odds and hazard that the outcome of interest occurred. For example, a higher OR suggests a higher odds of more HFD-90.

We performed 5 sensitivity analyses on the primary and 3 on the secondary outcomes. Primary outcome sensitivity analyses included both Bayesian and frequentist approaches. Bayesian sensitivity analysis included comparing HFD-90 by metformin treatment duration to placebo. Frequentist sensitivity analysis included comparing HFD-90 between metformin and placebo (1) regardless of treatment dose or duration and by (1) treatment doses (2) treatment durations and (3) placebo versus low dose and short duration versus all other treatment combinations (SAP, Supplemental File 2, see http://links.lww.com/AOSO/A487). while Secondary outcome sensitivity analysis included only the frequentist approach comparing metformin and placebo by (1) treatment doses (2) treatment durations and (3) placebo versus low dose and short duration versus all other treatment combinations. Finally, exploratory subgroup analysis was performed to understand the variation of the effect of metformin across the different subgroups such as age, frailty, surgical strata, sex, and surgical stress.

For all Bayesian models, a significant result will be indicated by a one-sided posterior probability greater than 0.975. For all Frequentist models, a one-sided *P*-value of less than 0.025 constituted statistical significance. A more detailed description of the data summary, data presentation, and data analysis (primary, secondary, and sensitivity analysis) is available in the SAP in the appendix.

## RESULTS

**FIGURE 1.**
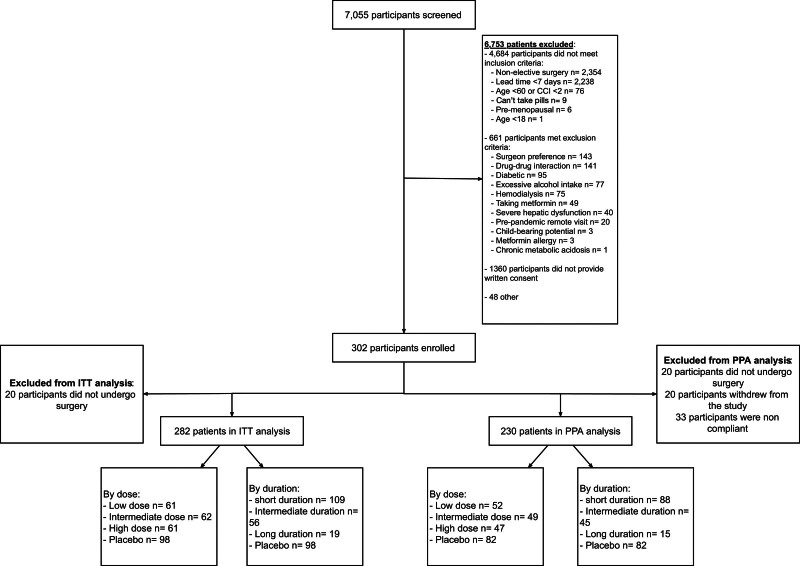
Consort diagram. Surgeon preference is when patients and providers opted to not pursue surgical intervention after evaluation in clinic. CCI indicates Charlson comorbidity index; ITT, intention-to-treat; PPA, per-protocol analysis.

We enrolled 302 patients, of which 196 (65%) were randomized to metformin (500 mg, N = 64 [33%]; 1000 mg, N = 66 [34%]; 1500 mg, N = 66 [34%]) and 106 (35%) to placebo (Fig. 1). Most patients (N = 183 [61%]) were pragmatically assigned to the preoperative short duration of study drug with N = 85 (28%) assigned to the intermediate and N = 34 (11.3%) the long. The dosage of study drug was equitably distributed between preoperative duration periods. The trial was terminated before the first interval analysis due to low enrollment in the setting of the COVID-19 pandemic and poor tolerance of perioperative study drug. As such, no interim results triggered response adaptive randomization. As previously planned, trial termination would result in public disclosure and a declaration of results.

**FIGURE 2.**
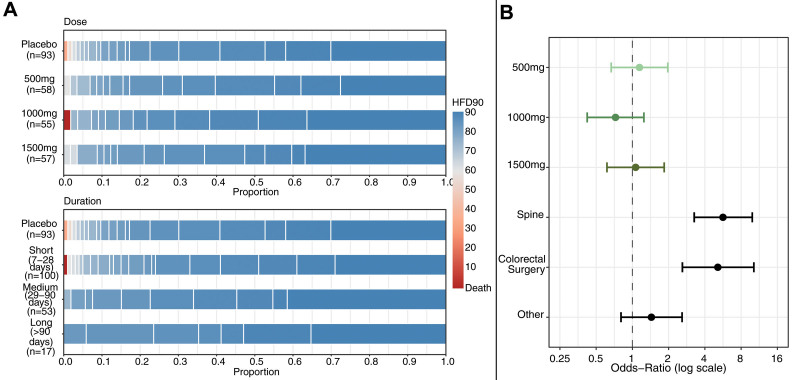
Distribution of 90-day hospital-free days in the Intention-to-treat group. A, Stacked proportion of post-discharge 90-day hospital-free days for each intervention and duration. Red represents worse outcomes and blue represents better outcomes. B, Forest plot representing model-estimated odds ratios. The dot is the posterior median and the line corresponds to the 95% credible interval.

After excluding the 20 patients that did not undergo an operation, 282 patients were included in the ITT analysis, and after excluding the 52 (18.4%) noncompliant patients (19 [6.7%] of which completely withdrew from the study), we included 230 patients into the PPA analysis. Noncompliance among the ITT analysis did not differ by metformin dose (*P* = 0.53) nor by duration (*P* = 0.92). ITT analysis baseline demographics, comorbid conditions, and surgical characteristics did not differ between groups (Supplemental Table 2, see http://links.lww.com/AOSO/A487). The following characteristics have also been described for the overall cohort, ITT, and PPA groups by treatment arm (Supplemental Tables 3 and 4, see http://links.lww.com/AOSO/A487), dose (Supplemental Tables 5–7, see http://links.lww.com/AOSO/A487), duration (Supplemental Tables 8–10, see http://links.lww.com/AOSO/A487), and surgical strata (Supplemental Tables 11–13, see http://links.lww.com/AOSO/A487). The median (interquartile range) HFD-90 was 88.0 days (85.0–90.0) with minimal variation across study drug dosages, summed across doses (87.0 days [85.0–90.0], 500 mg metformin; 89.0 [86.2–90.0], 1000 mg metformin; 88.0 [85.0–90.0], 1500 mg metformin; and 87.5 [85.0–90.0] placebo) or study drug durations, summed across durations (88.0 days [85.0–90.0] short duration, 88.0 [86.0–90.0] medium duration, 89.0 [85.0–90.0] long duration, 87.5 [85.0–90.0] placebo; Fig. 2A). When compared with placebo, a stronger benefit from 1000 mg metformin (mpOR = 0.74 [95% CrI, 0.42–1.25]) than 500 mg metformin (mpOR = 1.14 [0.67–1.97]) and 1500 mg metformin (1.06 [0.61–1.84]), although none were statistically significant (Fig. 2B). The PPA cohort (N = 230) demonstrated similar results (Supplemental Figures 1A, B, see http://links.lww.com/AOSO/A487).

Concerning surgical strata, patients that underwent spine or colorectal surgery had lower HFD-90 than the general surgery/surgical oncology patients (5.65 [3.26–9.90] and 5.12 [2.60–10.21], respectively), although the total number of patients in each group was small and uneven (Fig. 2B). Finally, results for the proportional odds assumption testing can be found under the Supplemental results (Supplemental File 1, see http://links.lww.com/AOSO/A487).

**FIGURE 3.**
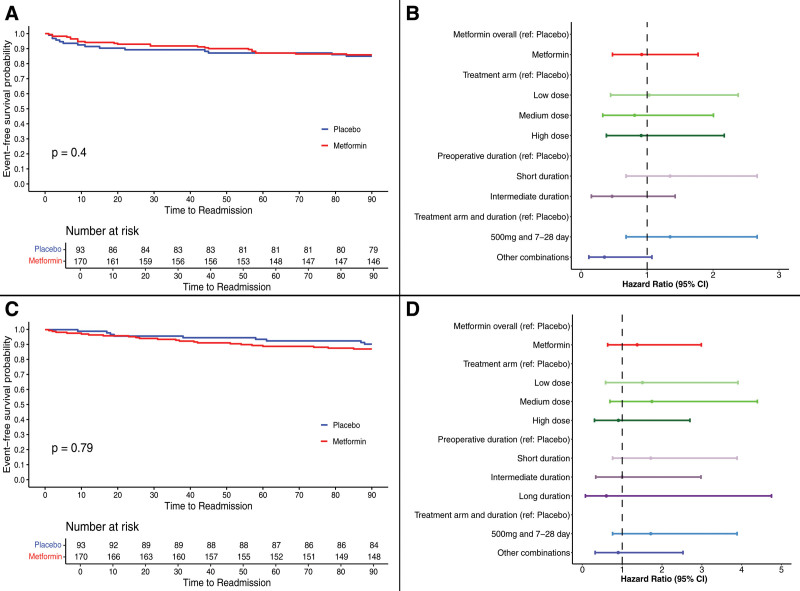
Rates of 90-day reoperation and readmission in the Intention-to-treat group. A, Kaplan-Meier curves representing the rate of a reoperation within 90 days of the initial operation. Nineteen participants that withdrew did not have complete information and were excluded. B, Forest plot representing the hazard ratios and 95% CI for primary and sensitivity analysis for the 90-day reoperation. A, Kaplan-Meier curves representing the rate of a readmission within 90 days of the initial operation, no events occurred for a long duration and was not included in the forest plot. Nineteen participants that withdrew did not have complete information and were excluded. B, Forest plot representing the hazard ratios and 95% CI for primary and sensitivity analysis for the 90-day readmission.

**FIGURE 4.**
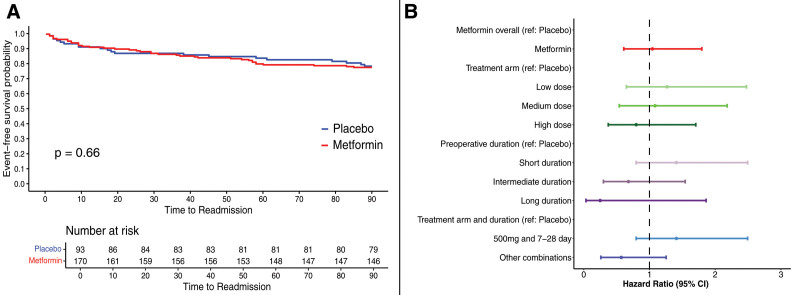
Rate of 90-day adverse event in the intention-to-treat group. A, Kaplan-Meier curves representing the rate of occurrence of an adverse event within 90 days of the operation. Nineteen participants that withdrew did not have complete information and were excluded. B, Forest plot representing the hazard ratios and 95% CI for primary and sensitivity analysis for the 90-day adverse events.

Secondary endpoint analysis showed no difference in the 90-day reoperation rates (13.0% vs 14.3%; OR = 1.08 [95% CI, 0.58–2.0]) (Supplemental Figures 2, 3A, see http://links.lww.com/AOSO/A487) and the occurrence of a reoperation over time were similar across the placebo and treatment group (HR [95% CI] = 0.91 [0.47–1.77]) (Figs. 3A, B). In addition, the 90-day rate of readmission did not vary significantly across the treatment and placebo group (12.0% vs 9.18%; OR = 1.45 [95% CI, 0.74–2.86]) (Supplemental Figures 3B, 4, see http://links.lww.com/AOSO/A487). Similarly, there was no statistically significant difference in the readmission rates over time following the procedure (HR = 1.37 [95% CI, 0.63–2.98]) (Figs. 3C, D). There was no difference in the rates of 90-day morbidity between metformin and placebo group (22.4% vs 21.5%; OR = 1.05 [95% CI, 0.56–1.93]) (Supplemental Figures 5, 6, see http://links.lww.com/AOSO/A487) nor the rate of occurrence of morbidity over time (HR = 1.04 [95% CI, 0.60–1.79]) (Figs. 4A, B). As in the primary analysis, the PPA cohort recapitulated the ITT findings (Supplemental Figures 2, 4, 5, 7–9, see http://links.lww.com/AOSO/A487).

### Sensitivity Analysis

Bayesian sensitivity analysis for the primary outcome comparing preoperative duration showed that among the ITT group, in comparison to placebo, the point estimates showed that there was a stronger benefit from medium duration (mpOR = 0.72 [95% CrI, 0.41–1.25]) than short (1.11 [0.69–1.79]) or long duration (1.04 [0.49–2.21]), although none were statistically significant (Fig. 2A, Supplemental Figure 10A, see http://links.lww.com/AOSO/A487). Similar results were also depicted in the PPA group (Supplemental Figures 1A, 10B, see http://links.lww.com/AOSO/A487).

The second sensitivity analysis recapitulated the primary analysis model using frequentist approach and showed similar results to the primary analysis in which there was a stronger benefit from 1000 mg metformin than 500 and 1500 mg metformin in comparison to placebo (Supplemental Figure 11, see http://links.lww.com/AOSO/A487). In addition, another sensitivity analysis using the frequentist approach comparing metformin regardless of the dose or duration to placebo showed again no difference in HFD-90 between the 2 groups (Supplemental Figure 11, see http://links.lww.com/AOSO/A487). Finally, similar to the Bayesian analysis, the frequentist sensitivity analysis of the primary outcome demonstrated that there was a stronger benefit from medium duration than short and long duration of preoperative metformin, although not statistically significant (Supplemental Figure 11, see http://links.lww.com/AOSO/A487). All those analyses were recapitulated in the PPA group (Supplemental Figure 12, see http://links.lww.com/AOSO/A487).

### Subgroup Analyses

In the ITT analysis, there was a statistically significant difference in HFD-90 between metformin and placebo groups for when we limited the cohort to participants with age less than the median age (67 years) of the overall cohort (OR [95% CI] = 2.41 [1.24–4.69], supplemental Figure 13A, see http://links.lww.com/AOSO/A487). While there was no difference in outcome in any of the other prespecified subgroups that included segregation by sex (Supplemental Figure 14A, see http://links.lww.com/AOSO/A487) frailty category (Supplemental Figure 15A, see http://links.lww.com/AOSO/A487), surgical strata (Supplemental Figure 16A, see http://links.lww.com/AOSO/A487), and operative stress score (Supplemental Figure 17A, see http://links.lww.com/AOSO/A487). The results were similar across the PPA analysis (Supplemental Figures 13–17, Panels B, see http://links.lww.com/AOSO/A487).

The results for the other secondary outcomes can be found under the Supplemental results (Supplemental File 1, Supplemental Figures 18–33, see http://links.lww.com/AOSO/A487). None of the secondary outcomes revealed statistically significant differences between treatment groups. Similarly, the results for the adverse events (AEs) have been listed under the Supplementary results (Supplemental File 1, Supplemental Figures 34–36, see http://links.lww.com/AOSO/A487). In brief, AEs were more common in metformin treatment arms, but these were mainly related to study drug tolerance and gastrointestinal side effects, which are well-known with metformin.

## DISCUSSION

In our REMAP trial, prehabilitation and postsurgical treatment with metformin did not increase HFD-90 following a major elective surgical intervention, nor did it decrease postoperative adverse events when compared with placebo. In the subgroup of participants that were younger than the median age of the study cohort metformin demonstrated a potential benefit through an increased HFD-90 in comparison to placebo. However, importantly, the study was underpowered due to early termination secondary to the COVID-19 pandemic. Furthermore, drug-related serious adverse events were infrequent with metformin allocation; however, there was an expected increase in gastrointestinal side effects when compared with placebo consistent with the known side effect profile of the treatment. This side effect profile contributed to poor study drug compliance resulting in withdrawals that also impacted trial enrollment and completion. The negative results that culminated from this trial need to be considered within the context of the lack of enrollment; therefore, we did not truly test our hypothesis limiting the conclusions from these data. However, numerous aspects of the methodology and results can and should inform future work.

Therapy concealment and randomization within a well-selected cohort optimally allow causal inference conclusions by minimizing biases and unmeasured confounding. However, traditional randomized controlled trials are slow, expensive, and inefficiently answer clinical questions.^12^ Novel trial strategies, including REMAP studies,^18–20^ have successfully tested hypotheses through synergizing trial methods within the data and infrastructure generated through patient care in the EHR. SPRY is the first REMAP study to enroll surgical patients. Although our superiority trial failed to reach an adaptative randomization sample size, limiting our ability to evaluate our primary hypothesis, SPRY demonstrates feasibility for REMAP trial designs among surgical patient populations. Specifically, we efficiently and automatically screened and recruited patients through the custom application while simultaneously notifying both the clinical and research teams of the potential trial participants. Further, the regulatory and EHR infrastructure provided the platform for testing additional hypotheses and facilitated the design of other novel perioperative trials within our healthsystem.^21^ Therefore, SPRY provided a foundational step toward creating a self-learning system integrating hypothesis testing naturally into daily healthcare activities.

On the heels of early successful Bayesian^20,22–24^ and embedded trials,^18–20^ SPRY was ultimately terminated before reaching the 500-patient adaptive randomization sample size largely driven by the COVID-19 pandemic. First, the safety and availability of elective major surgical interventions, our cohort of interest, came into question and nearly fully ceased for a period during the study interval. Second, the SPRY infrastructure and support staff were appropriately and rapidly adapted to support REMAP-CAP’s rapid and efficient evaluation of novel COVID-19 therapies in worldwide effort to inform the treatment of patients in an unprecedented pandemic.^20,25–30^ Third, the COVID-19 pandemic dramatically expanded the electronic healthcare visit. Although SPRY promptly adjusted, allowing for enrollment through virtual visits, this mechanism’s effect on study recruitment has an unknown effect on trial participation both during and following pandemic-related surgical restrictions. Beyond these enrollment limitations, although in line with other trial reports,^31^ the gastrointestinal side effects of metformin were high within the perioperative period affecting drug tolerability and compliance.^32^ As we failed to meet the sample size for enrollment. Therefore, the available data cannot adequately test our hypothesis that preoperative metformin would improve postoperative outcomes. As such, we neither can accept nor reject the null hypothesis. However, there are numerous potential reasons why SPRY may have had negative results if enrollment was achieved, including the heterogeneity of treatment effects across the surgical population, and inability to control for otherwise uncaptured confounders (eg, inequitable distributions of other prehabilitation), and misclassification of the EHR automated outcomes.

Evidence has accumulated supporting the systemic anti-inflammatory properties of metformin^33,34^ through its effect on the microbiome,^35^ muscle function,^36^ and cellular respiration,^37^ thus, mitigating and decreasing deleterious cellular inflammation.^38,39^ This anti-inflammatory characteristic of metformin has been demonstrated clinically through a retrospective evaluation that showed that perioperative metformin prescribing was associated with a reduction in the adjusted risk of both postoperative readmissions and mortality, 2 key aspects in the calculation of HFDs, among patients with type 2 diabetes.^11^ However, our prospective trial failed to support these findings in an evaluation of nondiabetic aged adults. As noted, SPRY did not reach the minimal sample size required for adaptive randomization. Nonetheless, in *a priori* determined subgroup analysis, we noted a statistically significant increase in HFD-90 among the subgroup of participants younger than the median age that used metformin perioperatively. Frailty was also assessed, and subgroup differences were not yielded. Therefore, despite the *a priori* selection of subgroup analyses, there is an ongoing risk of type I error. Although this is not a definitive answer for the effectiveness of metformin due to the discussed limitations, it will help shed light on the importance of continuing to explore the anti-aging effects of metformin. In addition, exploring the possible enhanced effects of metformin early in the aging process (<65 years), especially knowing that most clinical trials providing evidence for the frailty mitigating effects of metformin solely focus on elderly participants (>65 years old).^40–42^ Furthermore, ongoing clinical trials exploring the anti-inflammatory benefits of metformin^38,43,44^ will help provide a definitive answer concerning the clinical use of metformin as an anti-aging drive that increases functional reserve and improves patients’ outcomes.

The efficiency of the REMAP design linking to our healthcare systems' EHR data results with 3 resulting limitations in addition to the small sample size. First, study drug compliance was limited to self-report. Second, postoperative hospital admissions outside of our healthcare system were omitted from the HFD summation presumably leading to random and otherwise uncaptured missingness. Third, we primarily enrolled from our tertiary hospitals within the hospital system providing care to those within Southwestern Pennsylvania and the surrounding states yielding limited generalizability.

In conclusion, perioperative metformin did not increase 90-day HFD, nor did it decrease postoperative adverse events except in patients that were younger than 65 years old where we observed a significant increase in 90-day HFD in metformin treatment arm in comparison to placebo. However, considering the limitations of the study especially concerning participant enrollment, more studies are required to confirm this finding.

## ACKNOWLEDGMENTS

K.M. and H.N. were equal key contributors to concept and design with M.D.N, data acquisition, statistical analysis, and interpretation of data; drafted manuscript to completion; generated and reviewed the final documents. F.K., M.Q., and S.B. involved in key contributions to concept and design, data acquisition—main coauthor that worked on primary Bayesian analysis, and invaluable to interpretation of data; critically evaluated the order and presentation of data; reviewed the final documents. J.K., R.Z., C.W.S., K.V., M.D., A.M., B.M., B.Z., T.D.G., O.C.M., S.E., J.H.-M., A.B.N., T.R.B., and D.C.A. involved in key contributions to early discussions involving concept and design, data acquisition—invaluable for the reviewing the data analysis, and invaluable to interpretation of data; critically evaluated the first draft of the manuscript; reviewed the final documents. M.D.N. contributed to primary concept and design, data acquisition—all sources used, and interpretation of data; preliminary discussion and multiple rounds of edits for the drafted manuscript to completion; reviewed the final documents.

## Supplementary Material
