# Supplementary material for: Strategies to Promote Resiliency: A Randomized Embedded Multifactorial Adaptative Platform (REMAP) Clinical Trial to Study Interventions to Improve Recovery After Surgery in High-Risk Patients
Source: Ann Surg Open. 2025 Apr 2;6(2):e566. doi: 10.1097/AS9.0000000000000566 (PMC12185083; doi:10.1097/AS9.0000000000000566)
Supplement: Supplementary file 1 [file as9-6-e566-s001.pdf]

SUPPLEMENTAL RESULTS 5

PROPORTIONAL ODDS ASSUMPTION 5

SECONDARY OUTCOMES 5

SENSITIVITY ANALYSIS 6

SUPPLEMENTAL TABLE 1. CORE PROTOCOL AND SPRY-METFORMIN DOMAIN-SPECIFIC APPENDIX INCLUSION AND EXCLUSION CRITERIA 7

SUPPLEMENTAL TABLE 2. INTENTION-TO-TREAT ANALYSIS GROUPS' BASELINE CHARACTERISTICS AND DEMOGRAPHICS 8

SUPPLEMENTAL TABLE 3. OVERALL COHORTS' BASELINE CHARACTERISTICS AND DEMOGRAPHICS 10

SUPPLEMENTAL TABLE 4. PER-PROTOCOL ANALYSIS GROUPS' BASELINE CHARACTERISTICS AND DEMOGRAPHICS 13

SUPPLEMENTAL TABLE 5. OVERALL COHORTS' BASELINE CHARACTERISTICS AND DEMOGRAPHICS BY TREATMENT DOSE 16

SUPPLEMENTAL TABLE 6. INTENTION-TO-TREAT ANALYSIS GROUPS' BASELINE CHARACTERISTICS AND DEMOGRAPHICS BY TREATMENT DOSE 19

SUPPLEMENTAL TABLE 7. PER-PROTOCOL ANALYSIS GROUPS' BASELINE CHARACTERISTICS AND DEMOGRAPHICS BY TREATMENT DOSE 22

SUPPLEMENTAL TABLE 8. OVERALL COHORTS' BASELINE CHARACTERISTICS AND DEMOGRAPHICS BY TREATMENT DURATION 25

SUPPLEMENTAL TABLE 9. INTENTION-TO-TREAT ANALYSIS GROUPS' BASELINE CHARACTERISTICS AND DEMOGRAPHICS BY TREATMENT DURATION 28

SUPPLEMENTAL TABLE 10. PER-PROTOCOL ANALYSIS GROUPS' BASELINE CHARACTERISTICS AND DEMOGRAPHICS BY TREATMENT DURATION 31

SUPPLEMENTAL TABLE 11. OVERALL COHORTS' BASELINE CHARACTERISTICS AND DEMOGRAPHICS BY SURGICAL STRATA 34

SUPPLEMENTAL TABLE 12. INTENTION-TO-TREAT ANALYSIS GROUPS' BASELINE CHARACTERISTICS AND DEMOGRAPHICS BY SURGICAL STRATA 37

SUPPLEMENTAL TABLE 13. PER-PROTOCOL ANALYSIS GROUPS' BASELINE CHARACTERISTICS AND DEMOGRAPHICS BY SURGICAL STRATA 40

SUPPLEMENTAL FIGURE 1. HORIZONTAL STACKED BAR GRAPH AND FOREST PLOT DISPLAYING THE DISTRIBUTION OF 90-DAY HOSPITAL FREE DAYS IN THE PER-PROTOCOL ANALYSIS 43

SUPPLEMENTAL FIGURE 2. BAR GRAPH DISPLAYING THE RATE OF REOPERATION IN THE INTENTION-TO-TREAT AND PER-PROTOCOL ANALYSIS 44

SUPPLEMENTAL FIGURE 3. FOREST PLOT SHOWING THE RESULTS OF THE LOGISTIC REGRESSION MODEL FOR REOPERATION AND READMISSION IN THE INTENTION-TO-TREAT ANALYSIS 45

SUPPLEMENTAL FIGURE 4. BAR GRAPH DISPLAYING THE RATE OF READMISSION IN THE INTENTION-TO-TREAT AND PER-PROTOCOL ANALYSIS 46

SUPPLEMENTAL FIGURE 5. BAR GRAPH DISPLAYING THE RATE OF 90-DAY ADVERSE EVENTS IN THE INTENTION-TO-TREAT AND PER-PROTOCOL ANALYSIS 47

SUPPLEMENTAL FIGURE 6. FOREST PLOT SHOWING THE RESULTS OF THE LOGISTIC REGRESSION FOR THE 90-DAY ADVERSE EVENTS IN INTENTION-TO-TREAT ANALYSIS 48

SUPPLEMENTAL FIGURE 7. FOREST PLOT SHOWING THE RESULTS OF THE LOGISTIC REGRESSION FOR THE RATE OF REOPERATION AND READMISSION IN PER-PROTOCOL ANALYSIS 49

SUPPLEMENTAL FIGURE 8. KAPLAN-MEIER CURVES AND FOREST PLOT SHOWING THE RATE OF EVENT-FREE SURVIVAL AND RESULTS OF COX REGRESSION FOR THE 90-DAY REOPERATION AND READMISSION IN THE PER-PROTOCOL ANALYSIS 50

SUPPLEMENTAL FIGURE 9. KAPLAN-MEIER CURVES AND FOREST PLOT SHOWING THE RATE OF EVENT-FREE SURVIVAL AND RESULTS OF COX REGRESSION FOR THE 90-DAY ADVERSE EVENTS IN THE PER-PROTOCOL ANALYSIS 51

SUPPLEMENTAL FIGURE 10. FOREST PLOT SHOWING THE RESULTS OF THE BAYESIAN SENSITIVITY ANALYSIS IN THE INTENTION-TO-TREAT AND PER-PROTOCOL ANALYSIS 52

SUPPLEMENTAL FIGURE 11. FOREST PLOT SHOWING THE RESULTS OF THE FREQUENTIST SENSITIVITY ANALYSIS OF PRIMARY OUTCOME IN THE INTENTION-TO-TREAT ANALYSIS 53

SUPPLEMENTAL FIGURE 12. FOREST PLOT SHOWING THE RESULTS OF THE FREQUENTIST SENSITIVITY ANALYSIS OF PRIMARY OUTCOME IN THE PER-PROTOCOL ANALYSIS........ 54

SUPPLEMENTAL FIGURE 13. FOREST PLOT SHOWING THE RESULTS OF THE AGE SPECIFIC SUBGROUP ANALYSIS IN BOTH THE INTENTION-TO-TREAT AND PER-PROTOCOL ANALYSIS.. 55

SUPPLEMENTAL FIGURE 14. FOREST PLOT SHOWING THE RESULTS OF THE SEX SPECIFIC SUBGROUP ANALYSIS IN BOTH THE INTENTION-TO-TREAT AND PER-PROTOCOL ANALYSIS.. 56

SUPPLEMENTAL FIGURE 15. FOREST PLOT SHOWING THE RESULTS OF THE FRAILTY SPECIFIC SUBGROUP ANALYSIS IN BOTH THE INTENTION-TO-TREAT AND PER-PROTOCOL ANALYSIS 57

SUPPLEMENTAL FIGURE 16. FOREST PLOT SHOWING THE RESULTS OF THE SURGICAL STRATA SPECIFIC SUBGROUP ANALYSIS IN BOTH THE INTENTION-TO-TREAT AND PER-PROTOCOL ANALYSIS 58

SUPPLEMENTAL FIGURE 17. SENSITIVITY ANALYSIS OF THE DICHOTOMIZED CATEGORIES OF HFD-90 IN THE INTENTION-TO-TREAT ANALYSIS POPULATION BY ACTIVE DOSE 59

SUPPLEMENTAL FIGURE 18. SENSITIVITY ANALYSIS OF THE DICHOTOMIZED CATEGORIES OF HFD-90 IN THE INTENTION-TO-TREAT ANALYSIS POPULATION BY ACTIVE DURATION........ 61

SUPPLEMENTAL FIGURE 19. BAR GRAPH SHOWING THE RATE OF ICU (INTENSIVE CARE UNIT) ADMISSION BY INTERVENTION ARM IN BOTH THE INTENTION-TO-TREAT AND PER-PROTOCOL ANALYSIS 63

SUPPLEMENTAL FIGURE 20. FOREST PLOT SHOWING THE RESULTS OF THE LOGISTIC REGRESSION FOR THE RATE OF ICU (INTENSIVE CARE UNIT) ADMISSION IN INTENTION-TO-TREAT AND PER-PROTOCOL ANALYSIS 64

SUPPLEMENTAL FIGURE 21. BOX AND WHISKER PLOT SHOWING THE LENGTH OF POSTOPERATIVE ICU (INTENSIVE CARE UNIT) STAY BY INTERVENTION ARM IN BOTH THE INTENTION-TO-TREAT AND PER-PROTOCOL ANALYSIS 65

SUPPLEMENTAL FIGURE 22. BOX AND WHISKER PLOT SHOWING THE LENGTH OF POSTOPERATIVE HOSPITAL STAY BY INTERVENTION ARM IN BOTH THE INTENTION-TO-TREAT AND PER-PROTOCOL ANALYSIS 66

SUPPLEMENTAL FIGURE 23. BAR GRAPH SHOWING THE RATES OF DISCHARGE DESTINATION BY INTERVENTION ARM IN BOTH THE INTENTION-TO-TREAT AND PER-PROTOCOL ANALYSIS 67

SUPPLEMENTAL FIGURE 24. FOREST PLOT SHOWING THE RESULTS OF THE LOGISTIC REGRESSION FOR THE ODDS OF BEING DISCHARGED TO A SNF/LTAC IN THE INTENTION-TO-TREAT AND PER-PROTOCOL ANALYSIS 68

SUPPLEMENTAL FIGURE 25. BOX AND WHISKER PLOT SHOWING THE DISTRIBUTION OF 30-DAY ORGAN FAILURE FREE DAYS IN BOTH INTENTION-TO-TREAT AND PER-PROTOCOL ANALYSIS 69

SUPPLEMENTAL FIGURE 26. BAR GRAPH SHOWING THE RATE OF SURGICAL SITE INFECTION ACCORDING TO THE VASQUIP DEFINITION BY INTERVENTION ARM IN BOTH THE INTENTION-TO-TREAT AND PER-PROTOCOL ANALYSIS 70

SUPPLEMENTAL FIGURE 27. FOREST PLOT SHOWING THE RESULTS OF THE LOGISTIC REGRESSION FOR THE ODDS OF HAVING A SURGICAL SITE INFECTION ACCORDING TO THE VASQUIP DEFINITION IN THE INTENTION-TO-TREAT AND PER-PROTOCOL ANALYSIS 71

SUPPLEMENTAL FIGURE 28. BAR GRAPH SHOWING THE RATE OF SURGICAL SITE INFECTION ACCORDING TO THE VENTRAL HERNIA WORKING GROUP DEFINITION BY INTERVENTION ARM IN BOTH THE INTENTION-TO-TREAT AND PER-PROTOCOL ANALYSIS 72

SUPPLEMENTAL FIGURE 29. FOREST PLOT SHOWING THE RESULTS OF THE LOGISTIC REGRESSION FOR THE ODDS OF HAVING A SURGICAL SITE INFECTION ACCORDING TO THE VENTRAL HERNIA WORKING GROUP DEFINITION IN THE INTENTION-TO-TREAT AND PER-PROTOCOL ANALYSIS 73

SUPPLEMENTAL FIGURE 30. BAR GRAPH SHOWING THE RATE OF VENOUS-THROMBOEMBOLIC EVENTS BY INTERVENTION ARM IN BOTH THE INTENTION-TO-TREAT AND PER-PROTOCOL ANALYSIS 74

SUPPLEMENTAL FIGURE 31. FOREST PLOT SHOWING THE RESULTS OF THE LOGISTIC REGRESSION FOR THE ODDS OF HAVING A VENOUS-THROMBOEMBOLIC EVENT IN THE INTENTION-TO-TREAT AND PER-PROTOCOL ANALYSIS 75

SUPPLEMENTAL FIGURE 32. KAPLAN-MEIER CURVES AND FOREST PLOT SHOWING THE RATE OF VENOUS-THROMBOEMBOLIC (VTE) EVENT-FREE SURVIVAL AND RESULTS OF COX REGRESSION FOR THE 90-DAY VTE EVENTS IN THE INTENTION-TO-TREAT ANALYSIS 76

SUPPLEMENTAL FIGURE 33. KAPLAN-MEIER CURVES AND FOREST PLOT SHOWING THE RATE OF VENOUS-THROMBOEMBOLIC (VTE) EVENT-FREE SURVIVAL AND RESULTS OF COX REGRESSION FOR THE 90-DAY VTE EVENTS IN THE PER-PROTOCOL ANALYSIS 77

SUPPLEMENTAL FIGURE 34. BAR GRAPH SHOWING THE RATE OF GASTROINTESTINAL DISTURBANCES BY INTERVENTION ARM IN BOTH THE INTENTION-TO-TREAT AND PER-PROTOCOL ANALYSIS 78

SUPPLEMENTAL FIGURE 35. FOREST PLOT SHOWING THE RESULTS OF THE LOGISTIC REGRESSION FOR THE ODDS OF HAVING A GASTROINTESTINAL DISTURBANCE EVENT IN THE INTENTION-TO-TREAT AND PER-PROTOCOL ANALYSIS 79

SUPPLEMENTAL FIGURE 36. RATES OF SERIOUS ADVERSE EVENTS IN THE BOTH TREATMENT GROUPS ACROSS THE INTENTION-TO-TREAT AND PER-PROTOCOL ANALYSIS 80

Supplemental results

Proportional odds assumption

The ordinal logistic model estimates an odds ratio for each dose or duration that is constant across the 90-day ordinal scale of the primary endpoint, HFD-90. That is, the odds of being less than or equal to X is the same for all values of X on the 90-day ordinal scale. To explore the proportion odd assumption, we conduct a sensitivity analysis of the dichotomized categories of HFD-90 by active dose and active duration. Our analysis indicates that there is some evidence of a differential benefit in the lower part of the scale (small HFD-90 values) in the primary analysis model (**Supplemental Figure 17**). However, there is a high level of uncertainty in the lower values of the scale due to few observations. In the sensitivity analysis models, there is a clear differential effect in the medium and long durations with observations in the lower part of the scale being very few to none (**Supplemental Figure 18**). These data show there isn't a way to distinguish a treatment effect by duration. Rather, the data suggests that pre-operative durations represent different populations (e.g. patients with longer pre-operative durations have less emergent surgeries and a higher likelihood of post-surgical hospital free days).

Secondary outcomes

There were no significant differences in the rate of ICU admission following the operation in both ITT (metformin: 15 [8.1%] vs placebo: 10 [10.2%], OR [95%CI]=0.8 [0.3-1.9]) and PPA analysis (metformin: 12 [8.1%] vs placebo: 10 [12.2%], OR [95%CI]=0.3 [0.1-3.0]) (**Supplemental Figures 19-20**). Similarly, there was no significant difference in the length of stay in the ICU between the metformin and placebo groups (ITT mean±SD length of stay: 2.1±1.7 days vs 1.9±1.0 days, Beta-coefficient [95%CI, p-value]= 0.27 [-0.96-1.50, 0.67], PPA mean±SD length of stay: 2.2±1.8 days vs 1.9±1.0, Beta-coefficient [95%CI, p-value]= 0.30 [-1.04-1.66, 0.67]) (**Supplemental Figure 21**).

Although not statistically significant, the metformin group had a shorter hospital length of stay in comparison to the placebo group in both the ITT (3.3±4.5 days vs 4.1±7.3 days, Beta-coefficient [95%CI, p-value]= -0.76 [-2.22-0.69, 0.15) and PPA analysis (3.3±4.6 days vs 4.2±7.8 days, Beta-coefficient [95%CI, p-value]= -0.80 [-2.18-0.58, 0.13]) (**Supplemental Figure 22**). Additionally, less patients were discharged to skilled nursing facilities from the intervention group in comparison to the placebo group (13 [7.1%] vs 8 [8.2%]; OR [95% CI]=0.8 [0.4-2.2]), which was again recapitulated in the PPA group (11 [7.4%] vs 8 [9.8%]; OR [95% CI]= 0.7 [0.3-1.9]) (**Supplemental Figure 23-24**). There were no deaths or discharges to hospice following the initial hospitalization.

In both ITT and PPA analysis the metformin and placebo groups had similar organ failure free days which was 29.9±0.3 days (Beta-coefficient [95%CI, p-value]= 0.01 [-0.07-0.08, 0.54]) (**Supplemental Figure 25**).

Concerning surgical site infections, 4 (4.1%) patients had a surgical site infection from the placebo group in the ITT analysis while 12 (6.5%) patients from the metformin group (OR [95% CI]= 1.7 [0.5-5.4]) (**Supplemental Figure 26-27 panels A**). Similarly, the PPA analysis showed similar results (metformin: 12 [8.1%] vs placebo: 3 [3.6%], OR [95% CI]= 2.3 [0.6-8.5]) (**Supplemental Figure 26-27 panels B**). Moreover, we looked at the surgical site occurrence, where metformin group had more surgical site occurrences in comparison to the placebo group in both the ITT (19 [10.3%] vs 8 [8.2%], OR [95% CI]= 1.3 [0.6-3.2]) (**Supplemental Figure 28-29 panels A**) and PPA analysis (18 [12.1%] vs 6 [7.3%], OR [95% CI]= 1.7 [0.7-4.6]) (**Supplemental Figure 28-29 panels B**).

Finally, there were no differences in the rate of venous thromboembolic events in both ITT (metformin: 2 [1.1%] vs placebo: 2 [2.0%], OR [95% CI]= 0.5 [0.1-3.9]) (**Supplemental Figure 30-31 panels A)** and PPA analysis (metformin: 1 [0.7%] vs placebo: 2 [2.4%], OR [95% CI]= 0.3 [0.1-3.0]) (**Supplemental Figure 30-31 panels B**). Similarly, there is no difference in the occurrence of the venous thromboembolic events overtime between placebo and metformin groups (**Supplemental Figure 32-33**; ITT: HR [95% CI] = 0.5 [0.1-3.8], PPA: 0.3 [0.1-3.0]).

Adverse events

The rate of gastrointestinal disturbances was significantly higher in the group receiving metformin in comparison to the placebo group (ITT: 30 [16.3%] vs 6. [6.1%], OR [95% CI]=2.98 [1.19-7.44], PPA: 19 [12.8%] vs 4 [4.9%], OR [95% CI]=) (**Supplement Figure 34-35**). The overall rate of serious adverse events (SEA) was 50 (27.2%) in the metformin group, however, only 9 (4.9%) are related to the drug and 20 (10.9%) possibly related. Out of which 13 (7.1%, 6 [3.3%] related and 7 [3.8%] possibly related] are mild SEA, and 16 (8.7%, 3 [1.6%] related and 13 [7.1%] possibly related] are moderate SEA. There were only 6 [3.3%] severe or life threatening/disabling SEA that none were related to metformin. As for the placebo group only 6 [6.1%] SEA were related or possibly related to the placebo pill which were mild SEA (**Supplement Figure 36A**). The rates of SEA were slightly lower in the PPA group (**Supplement Figure 36B**).

Sensitivity analysis

Sensitivity analysis looking at the secondary outcomes using different combinations of dose and duration showed similar results as the primary analysis for the secondary outcomes.

SUPPLEMENTAL TABLE 1. Core protocol and SPRY-Metformin Domain-Specific appendix inclusion and exclusion criteria

**SPRY**
Inclusion criteria
- ≥18 years of age
- Evaluation within the UPMC healthcare system at any preoperative elective clinic
- Planned surgical intervention ≥7 and <365 days after the preoperative encounter
Exclusion criteria
- Surgeon determines that inclusion may be potentially harmful
- Non-elective surgical intervention
- Patient was part of SPRY within the last 90 days
**SPRY-Metformin**
Inclusion criteria
- Men and post-menopausal women who are ≥60 years of age or are <60 years of age with a Charlson Comorbidity Index >2
- Participant can swallow medication in pill form
Exclusion criteria
- Type I or type II diabetes
- Metformin use within the last 6 months
- Allergy to metformin
- Acute or chronic metabolic acidosis with or without coma
- History of lactic acidosis
- History of excessive alcohol intake
- Severe hepatic dysfunction
- Acute or chronic metabolic acidosis
- Hemodialysis, end-stage renal disease or estimated glomerular filtration rate <45 in the 30 days prior to or on the day of in-person screening

SUPPLEMENTAL TABLE 2. Intention-to-treat analysis groups' baseline characteristics and demographics

	Placebo (n=98)	Metformin (n=184)
**Age** (mean±SD)	67.7±5.8	68.8±6.9
**Sex** n(%)		
Female	46(46.9%)	80(43.5%)
Male	52(53.1%)	104(56.5%)
**Race** n(%)		
Black	5(5.1%)	10(5.4%)
White	93(94.9%)	167(90.8%)
Other	0 (0%)	3(1.6%)
Unknown	0 (0%)	4(2.2%)
**Ethnicity** n(%)		
Not Hispanic or Latino	98(100%)	178(96.7%)
Hispanic or Latino	0 (0%)	1(0.5%)
Unknown	0 (0%)	5(2.7%)
**Risk analysis index category** n(%)		
Robust	80(81.6%)	145(78.8%)
Normal	8(8.2%)	24(13%)
Frail	9(9.2%)	13(7.1%)
Very Frail	1(1%)	2(1.1%)
**Surgical strata** n(%)		
Spine	34(34.7%)	48(26.1%)
General surgery/Surgical oncology	33(33.7%)	74(40.2%)
Colorectal surgery	13(13.3%)	23(12.5%)
Other	18(18.4%)	39(21.2%)
**Smoking status** n(%)		
Never	45(45.9%)	101(54.9%)
Passive	1(1%)	0 (0%)
Quit	44(44.9%)	69(37.5%)
Yes	7(7.1%)	12(6.5%)
Unknown	1(1%)	2(1.1%)
**Body mass index** (mean±SD)	29.9 ± 6.3	29.9 ± 5.6
**Coronary artery disease** n(%)		
No	83(84.7%)	159(86.4%)
Yes	15(15.3%)	25(13.6%)
**Cancer** n(%)		
No	67(68.4%)	133(72.3%)

Yes	31(31.6%)	51(27.7%)
**Congestive heart failure** n(%)		
No	96(98%)	181(98.4%)
Yes	2(2%)	3(1.6%)
**Chronic obstructive pulmonary disease** n(%)		
No	86(87.8%)	169(91.8%)
Yes	12(12.2%)	15(8.2%)
**Depression** n(%)		
No	76(77.6%)	151(82.1%)
Yes	22(22.4%)	33(17.9%)
**Deep vein thrombosis** n(%)		
No	96(98%)	176(95.7%)
Yes	2(2%)	8(4.3%)
**Osteoarthritis** n(%)		
No	82(83.7%)	153(83.2%)
Yes	16(16.3%)	31(16.8%)
**Pulmonary embolism** n(%)		
No	96(98%)	175(95.1%)
Yes	2(2%)	9(4.9%)
**Stroke** n(%)		
No	94(95.9%)	177(96.2%)
Yes	4(4.1%)	7(3.8%)
**Peripheral vascular disease** n(%)		
No	95(96.9%)	177(96.2%)
Yes	3(3.1%)	7(3.8%)
**Year of randomization** n(%)		
2019	13(13.3%)	23(12.5%)
2020	16(16.3%)	24(13%)
2021	56(57.1%)	111(60.3%)
2022	13(13.3%)	26(14.1%)

SUPPLEMENTAL TABLE 3. Overall cohorts' baseline characteristics and demographics

	Placebo	Metformin
**Age** (mean±SD)	67.8 ± 5.6	68.6 ± 7.2
**Sex** n(%)		
Female	50(47.2%)	84(42.9%)
Male	56(52.8%)	112(57.1%)
**Race** n(%)		
Black	5(4.7%)	10(5.1%)
White	101(95.3%)	178(90.8%)
Other	0	4(2%)
Unknown	0	4(2%)
**Ethnicity** n(%)		
Not Hispanic or Latino	106(100%)	189(96.4%)
Hispanic or Latino	0	1(0.5%)
Unknown	0	6(3.1%)
**Risk analysis index category** n(%)		
Robust	86(81.1%)	155(79.1%)
Normal	9(8.5%)	26(13.3%)
Frail	9(8.5%)	13(6.6%)
Very Frail	2(1.9%)	2(1%)
**Surgical strata** n(%)		
No operation	8(7.5%)	12(6.1%)
Spine	34(32.1%)	48(24.5%)

General surgery/Surgical oncology	33(31.1%)	74(37.8%)
Colorectal surgery	13(12.3%)	23(11.7%)
Other	18(17%)	39(19.9%)
**Smoking status** n(%)		
Never	49(46.2%)	106(54.1%)
Passive	1(0.9%)	0
Quit	48(45.3%)	74(37.8%)
Yes	7(6.6%)	14(7.1%)
Unknown	1(0.9%)	2(1%)
**Body mass index** (mean±SD)	30.2 ± 6.4	30.3 ± 7.0
**Coronary artery disease** n(%)		
No	91(85.8%)	170(86.7%)
Yes	15(14.2%)	26(13.3%)
**Cancer** n(%)		
No	73(68.9%)	142(72.4%)
Yes	33(31.1%)	54(27.6%)
**Congestive heart failure** n(%)		
No	104(98.1%)	193(98.5%)
Yes	2(1.9%)	3(1.5%)
**Chronic obstructive pulmonary disease** n(%)		
No	94(88.7%)	179(91.3%)
Yes	12(11.3%)	17(8.7%)
**Depression** n(%)		
No	81(76.4%)	160(81.6%)

Yes	25(23.6%)	36(18.4%)
**Deep vein thrombosis** n(%)		
No	104(98.1%)	188(95.9%)
Yes	2(1.9%)	8(4.1%)
**Osteoarthritis** n(%)		
No	88(83%)	162(82.7%)
Yes	18(17%)	34(17.3%)
**Pulmonary embolism** n(%)		
No	102(96.2%)	187(95.4%)
Yes	4(3.8%)	9(4.6%)
**Stroke** n(%)		
No	102(96.2%)	189(96.4%)
Yes	4(3.8%)	7(3.6%)
**Peripheral vascular disease** n(%)		
No	103(97.2%)	189(96.4%)
Yes	3(2.8%)	7(3.6%)
**Year of randomization** n(%)		
2019	13(12.3%)	23(11.7%)
2020	17(16%)	27(13.8%)
2021	62(58.5%)	118(60.2%)
2022	14(13.2%)	28(14.3%)

SUPPLEMENTAL TABLE 4. Per-protocol analysis groups' baseline characteristics and demographics

	Placebo	Metformin
**Age** (mean±SD)	68.1 ± 5.8	68.2 ± 6.6
**Sex** n(%)		
Female	40(48.8%)	63(42.6%)
Male	42(51.2%)	85(57.4%)
**Race** n(%)		
Black	4(4.9%)	9(6.1%)
White	78(95.1%)	132(89.2%)
Other	0	3(2%)
Unknown	0	4(2.7%)
**Ethnicity** n(%)		
Not Hispanic or Latino	82(100%)	143(96.6%)
Hispanic or Latino	0	1(0.7%)
Unknown	0	4(2.7%)
**Risk analysis index category** n(%)		
Robust	68(82.9%)	119(80.4%)
Normal	7(8.5%)	18(12.2%)
Frail	6(7.3%)	9(6.1%)
Very Frail	1(1.2%)	2(1.4%)
**Surgical strata** n(%)		
Spine	31(37.8%)	40(27%)
General surgery/Surgical oncology	27(32.9%)	62(41.9%)

Colorectal surgery	10(12.2%)	19(12.8%)
Other	14(17.1%)	27(18.2%)
**Smoking status** n(%)		
Never	43(52.4%)	79(53.4%)
Passive	1(1.2%)	0
Quit	35(42.7%)	57(38.5%)
Yes	3(3.7%)	10(6.8%)
Unknown	0	2(1.4%)
**Body mass index** (mean±SD)	30.4 ± 6.6	29.9 ± 5.4
**Coronary artery disease** n(%)		
No	69(84.1%)	128(86.5%)
Yes	13(15.9%)	20(13.5%)
**Cancer** n(%)		
No	59(72%)	108(73%)
Yes	23(28%)	40(27%)
**Congestive heart failure** n(%)		
No	81(98.8%)	147(99.3%)
Yes	1(1.2%)	1(0.7%)
**Chronic obstructive pulmonary disease** n(%)		
No	73(89%)	136(91.9%)
Yes	9(11%)	12(8.1%)
**Depression** n(%)		
No	64(78%)	124(83.8%)
Yes	18(22%)	24(16.2%)
**Deep vein thrombosis** n(%)		

No	80(97.6%)	141(95.3%)
Yes	2(2.4%)	7(4.7%)
**Osteoarthritis** n(%)		
No	68(82.9%)	124(83.8%)
Yes	14(17.1%)	24(16.2%)
**Pulmonary embolism** n(%)		
No	80(97.6%)	141(95.3%)
Yes	2(2.4%)	7(4.7%)
**Stroke n**(%)		
No	79(96.3%)	142(95.9%)
Yes	3(3.7%)	6(4.1%)
**Peripheral vascular disease** n(%)		
No	80(97.6%)	143(96.6%)
Yes	2(2.4%)	5(3.4%)
**Year of randomization** n(%)		
2019	10(12.2%)	14(9.5%)
2020	9(11%)	15(10.1%)
2021	50(61%)	94(63.5%)
2022	13(15.9%)	25(16.9%)

SUPPLEMENTAL TABLE 5. Overall cohorts' baseline characteristics and demographics by treatment dose

	Low dose metformin	Intermediate dose metformin	High dose metformin	Placebo
**Age** (mean±SD)	69.1 ± 6.5	68.4 ± 7.5	68.6 ± 7.6	67.8 ± 5.6
**Sex** n(%)				
Female	32(50%)	27(40.9%)	25(37.9%)	50(47.2%)
Male	32(50%)	39(59.1%)	41(62.1%)	56(52.8%)
**Race** n(%)				
Black	1(1.6%)	5(7.6%)	4(6.1%)	5(4.7%)
White	59(92.2%)	59(89.4%)	60(90.9%)	101(95.3%)
Other	1(1.6%)	2(3%)	1(1.5%)	0
Unknown	3(4.7%)	0	1(1.5%)	0
**Ethnicity** n(%)				
Not Hispanic or Latino	61(95.3%)	64(97%)	64(97%)	106(100%)
Hispanic or Latino	0	1(1.5%)	0	0
Unknown	3(4.7%)	1(1.5%)	2(3%)	0
**Risk analysis index category** n(%)				
Robust	46(71.9%)	57(86.4%)	52(78.8%)	86(81.1%)
Normal	9(14.1%)	7(10.6%)	10(15.2%)	9(8.5%)
Frail	8(12.5%)	2(3%)	3(4.5%)	9(8.5%)
Very Frail	1(1.6%)	0	1(1.5%)	2(1.9%)
**Surgical strata** n(%)				
No operation	3(4.7%)	4(6.1%)	5(7.6%)	8(7.5%)
Spine	17(26.6%)	13(19.7%)	18(27.3%)	34(32.1%)

General surgery/Surgical oncology	25(39.1%)	25(37.9%)	24(36.4%)	33(31.1%)
Colorectal surgery	6(9.4%)	9(13.6%)	8(12.1%)	13(12.3%)
Other	13(20.3%)	15(22.7%)	11(16.7%)	18(17%)
**Smoking status** n(%)				
Never	38(59.4%)	34(51.5%)	34(51.5%)	49(46.2%)
Passive	0	0	0	1(0.9%)
Quit	21(32.8%)	25(37.9%)	28(42.4%)	48(45.3%)
Yes	4(6.2%)	7(10.6%)	3(4.5%)	7(6.6%)
Unknown	1(1.6%)	0	1(1.5%)	1(0.9%)
**Body mass index** (mean±SD)	30.8 ± 5.1	29.6 ± 5.8	30.7 ± 9.3	30.2 ± 6.4
**Coronary artery disease** n(%)				
No	56(87.5%)	58(87.9%)	56(84.8%)	91(85.8%)
Yes	8(12.5%)	8(12.1%)	10(15.2%)	15(14.2%)
**Cancer** n(%)				
No	40(62.5%)	51(77.3%)	51(77.3%)	73(68.9%)
Yes	24(37.5%)	15(22.7%)	15(22.7%)	33(31.1%)
**Congestive heart failure** n(%)				
No	63(98.4%)	65(98.5%)	65(98.5%)	104(98.1%)
Yes	1(1.6%)	1(1.5%)	1(1.5%)	2(1.9%)
**Chronic obstructive pulmonary disease** n(%)				
No	61(95.3%)	60(90.9%)	58(87.9%)	94(88.7%)
Yes	3(4.7%)	6(9.1%)	8(12.1%)	12(11.3%)
**Depression** n(%)				
No	57(89.1%)	52(78.8%)	51(77.3%)	81(76.4%)

Yes	7(10.9%)	14(21.2%)	15(22.7%)	25(23.6%)
**Deep vein thrombosis** n(%)				
No	59(92.2%)	64(97%)	65(98.5%)	104(98.1%)
Yes	5(7.8%)	2(3%)	1(1.5%)	2(1.9%)
**Osteoarthritis** n(%)				
No	52(81.2%)	58(87.9%)	52(78.8%)	88(83%)
Yes	12(18.8%)	8(12.1%)	14(21.2%)	18(17%)
**Pulmonary embolism** n(%)				
No	62(96.9%)	60(90.9%)	65(98.5%)	102(96.2%)
Yes	2(3.1%)	6(9.1%)	1(1.5%)	4(3.8%)
**Stroke** n(%)				
No	61(95.3%)	62(93.9%)	66(100%)	102(96.2%)
Yes	3(4.7%)	4(6.1%)	0	4(3.8%)
**Peripheral vascular disease** n(%)				
No	59(92.2%)	64(97%)	66(100%)	103(97.2%)
Yes	5(7.8%)	2(3%)	0	3(2.8%)
**Year of randomization** n(%)				
2019	8(12.5%)	8(12.1%)	7(10.6%)	13(12.3%)
2020	9(14.1%)	8(12.1%)	10(15.2%)	17(16%)
2021	39(60.9%)	38(57.6%)	41(62.1%)	62(58.5%)
2022	8(12.5%)	12(18.2%)	8(12.1%)	14(13.2%)

SUPPLEMENTAL TABLE 6. Intention-to-treat analysis groups' baseline characteristics and demographics by treatment dose

	Low dose metformin	Intermediate dose metformin	High dose metformin	Placebo
**Age** (mean±SD)	69.2 ± 6.4	68.5 ± 7.5	68.9 ± 6.8	67.8 ± 5.8
**Sex** n(%)				
Female	31(50.8%)	25(40.3%)	24(39.3%)	46(46.9%)
Male	30(49.2%)	37(59.7%)	37(60.7%)	52(53.1%)
**Race** n(%)				
Black	1(1.6%)	5(8.1%)	4(6.6%)	5(5.1%)
White	56(91.8%)	56(90.3%)	55(90.2%)	93(94.9%)
Other	1(1.6%)	1(1.6%)	1(1.6%)	0
Unknown	3(4.9%)	0	1(1.6%)	0
**Ethnicity** n(%)				
Not Hispanic or Latino	59(96.7%)	60(96.8%)	59(96.7%)	98(100%)
Hispanic or Latino	0	1(1.6%)	0	0
Unknown	2(3.3%)	1(1.6%)	2(3.3%)	0
**Risk analysis index category** n(%)				
Robust	44(72.1%)	53(85.5%)	48(78.7%)	80(81.6%)
Normal	8(13.1%)	7(11.3%)	9(14.8%)	8(8.2%)
Frail	8(13.1%)	2(3.2%)	3(4.9%)	9(9.2%)
Very Frail	1(1.6%)	0	1(1.6%)	1(1%)
**Surgical strata** n(%)				
Spine	17(27.9%)	13(21%)	18(29.5%)	34(34.7%)
General surgery/Surgical oncology	25(41%)	25(40.3%)	24(39.3%)	33(33.7%)

Colorectal surgery	6(9.8%)	9(14.5%)	8(13.1%)	13(13.3%)
Other	13(21.3%)	15(24.2%)	11(18%)	18(18.4%)
**Smoking status** n(%)				
Never	36(59%)	33(53.2%)	32(52.5%)	45(45.9%)
Passive	0	0	0	1(1%)
Quit	20(32.8%)	23(37.1%)	26(42.6%)	44(44.9%)
Yes	4(6.6%)	6(9.7%)	2(3.3%)	7(7.1%)
Unknown	1(1.6%)	0	1(1.6%)	1(1%)
**Body mass index** (mean±SD)	30.9 ± 5.1	29.1± 5.4	29.8 ± 6.1	29.9 ± 6.3
**Coronary artery disease** n(%)				
No	54(88.5%)	54(87.1%)	51(83.6%)	83(84.7%)
Yes	7(11.5%)	8(12.9%)	10(16.4%)	15(15.3%)
**Cancer** n(%)				
No	38(62.3%)	48(77.4%)	47(77%)	67(68.4%)
Yes	23(37.7%)	14(22.6%)	14(23%)	31(31.6%)
**Congestive heart failure** n(%)				
No	60(98.4%)	61(98.4%)	60(98.4%)	96(98%)
Yes	1(1.6%)	1(1.6%)	1(1.6%)	2(2%)
**Chronic obstructive pulmonary disease** n(%)				
No	58(95.1%)	57(91.9%)	54(88.5%)	86(87.8%)
Yes	3(4.9%)	5(8.1%)	7(11.5%)	12(12.2%)
**Depression** n(%)				
No	54(88.5%)	50(80.6%)	47(77%)	76(77.6%)
Yes	7(11.5%)	12(19.4%)	14(23%)	22(22.4%)
**Deep vein thrombosis** n(%)				

No	56(91.8%)	60(96.8%)	60(98.4%)	96(98%)
Yes	5(8.2%)	2(3.2%)	1(1.6%)	2(2%)
**Osteoarthritis** n(%)				
No	49(80.3%)	55(88.7%)	49(80.3%)	82(83.7%)
Yes	12(19.7%)	7(11.3%)	12(19.7%)	16(16.3%)
**Pulmonary embolism** n(%)				
No	59(96.7%)	56(90.3%)	60(98.4%)	96(98%)
Yes	2(3.3%)	6(9.7%)	1(1.6%)	2(2%)
**Stroke** n(%)				
No	58(95.1%)	58(93.5%)	61(100%)	94(95.9%)
Yes	3(4.9%)	4(6.5%)	0	4(4.1%)
**Peripheral vascular disease** n(%)				
No	56(91.8%)	60(96.8%)	61(100%)	95(96.9%)
Yes	5(8.2%)	2(3.2%)	0	3(3.1%)
**Year of randomization** n(%)				
2019	8(13.1%)	8(12.9%)	7(11.5%)	13(13.3%)
2020	8(13.1%)	7(11.3%)	9(14.8%)	16(16.3%)
2021	37(60.7%)	36(58.1%)	38(62.3%)	56(57.1%)
2022	8(13.1%)	11(17.7%)	7(11.5%)	13(13.3%)

SUPPLEMENTAL TABLE 7. Per-protocol analysis groups' baseline characteristics and demographics by treatment dose

	Low dose metformin	Intermediate dose metformin	High dose metformin	Placebo
**Age** (mean±SD)	68.8 ± 6.5	67.4 ± 6.8	68.2 ± 6.6	68.1 ± 5.8
**Sex** n(%)				
Female	26(50%)	19(38.8%)	18(38.3%)	40(48.8%)
Male	26(50%)	30(61.2%)	29(61.7%)	42(51.2%)
**Race** n(%)				
Black	1(1.9%)	5(10.2%)	3(6.4%)	4(4.9%)
White	47(90.4%)	43(87.8%)	42(89.4%)	78(95.1%)
Other	1(1.9%)	1(2%)	1(2.1%)	0
Unknown	3(5.8%)	0	1(2.1%)	0
**Ethnicity** n(%)				
Not Hispanic or Latino	50(96.2%)	47(95.9%)	46(97.9%)	82(100%)
Hispanic or Latino	0	1(2%)	0	0
Unknown	2(3.8%)	1(2%)	1(2.1%)	0
**Risk analysis index category** n(%)				
Robust	39(75%)	42(85.7%)	38(80.9%)	68(82.9%)
Normal	7(13.5%)	5(10.2%)	6(12.8%)	7(8.5%)
Frail	5(9.6%)	2(4.1%)	2(4.3%)	6(7.3%)
Very Frail	1(1.9%)	0	1(2.1%)	1(1.2%)
**Surgical strata** n(%)				
Spine	16(30.8%)	11(22.4%)	13(27.7%)	31(37.8%)
General surgery/Surgical oncology	20(38.5%)	22(44.9%)	20(42.6%)	27(32.9%)

Colorectal surgery	6(11.5%)	6(12.2%)	7(14.9%)	10(12.2%)
Other	10(19.2%)	10(20.4%)	7(14.9%)	14(17.1%)
**Smoking status** n(%)				
Never	31(59.6%)	26(53.1%)	22(46.8%)	43(52.4%)
Passive	0	0	0	1(1.2%)
Quit	17(32.7%)	17(34.7%)	23(48.9%)	35(42.7%)
Yes	3(5.8%)	6(12.2%)	1(2.1%)	3(3.7%)
Unknown	1(1.9%)	0	1(2.1%)	0
**Body mass index** (mean±SD)	30.8 ± 5.3	28.4 ± 4.7	30.5 ± 6.0	30.4 ± 6.6
**Coronary artery disease** n(%)				
No	46(88.5%)	44(89.8%)	38(80.9%)	69(84.1%)
Yes	6(11.5%)	5(10.2%)	9(19.1%)	13(15.9%)
**Cancer** n(%)				
No	33(63.5%)	37(75.5%)	38(80.9%)	59(72%)
Yes	19(36.5%)	12(24.5%)	9(19.1%)	23(28%)
**Congestive heart failure** n(%)				
No	52(100%)	49(100%)	46(97.9%)	81(98.8%)
Yes	0	0	1(2.1%)	1(1.2%)
**Chronic obstructive pulmonary disease** n(%)				
No	50(96.2%)	45(91.8%)	41(87.2%)	73(89%)
Yes	2(3.8%)	4(8.2%)	6(12.8%)	9(11%)
**Depression** n(%)				
No	46(88.5%)	39(79.6%)	39(83%)	64(78%)
Yes	6(11.5%)	10(20.4%)	8(17%)	18(22%)
**Deep vein thrombosis** n(%)				

No	47(90.4%)	48(98%)	46(97.9%)	80(97.6%)
Yes	5(9.6%)	1(2%)	1(2.1%)	2(2.4%)
**Osteoarthritis** n(%)				
No	42(80.8%)	44(89.8%)	38(80.9%)	68(82.9%)
Yes	10(19.2%)	5(10.2%)	9(19.1%)	14(17.1%)
**Pulmonary embolism** n(%)				
No	50(96.2%)	45(91.8%)	46(97.9%)	80(97.6%)
Yes	2(3.8%)	4(8.2%)	1(2.1%)	2(2.4%)
**Stroke** n(%)				
No	50(96.2%)	45(91.8%)	47(100%)	79(96.3%)
Yes	2(3.8%)	4(8.2%)	0	3(3.7%)
**Peripheral vascular disease** n(%)				
No	48(92.3%)	48(98%)	47(100%)	80(97.6%)
Yes	4(7.7%)	1(2%)	0	2(2.4%)
**Year of randomization** n(%)				
2019	6(11.5%)	3(6.1%)	5(10.6%)	10(12.2%)
2020	6(11.5%)	4(8.2%)	5(10.6%)	9(11%)
2021	33(63.5%)	31(63.3%)	30(63.8%)	50(61%)
2022	7(13.5%)	11(22.4%)	7(14.9%)	13(15.9%)

SUPPLEMENTAL TABLE 8. Overall cohorts' baseline characteristics and demographics by treatment duration

	Short duration	Intermediate duration	Long duration	Placebo
**Age** (mean±SD)	68.0 ± 7.5	69.2 ± 7.0	70.9 ± 5.5	67.8 ± 5.6
**Sex** n(%)				
Female	49(41.5%)	26(45.6%)	9(42.9%)	50(47.2%)
Male	69(58.5%)	31(54.4%)	12(57.1%)	56(52.8%)
**Race** n(%)				
Black	5(4.2%)	5(8.8%)	0	5(4.7%)
White	110(93.2%)	49(86%)	19(90.5%)	101(95.3%)
Other	2(1.7%)	2(3.5%)	0	0
Unknown	1(0.8%)	1(1.8%)	2(9.5%)	0
**Ethnicity** n(%)				
Not Hispanic or Latino	114(96.6%)	55(96.5%)	20(95.2%)	106(100%)
Hispanic or Latino	1(0.8%)	0	0	0
Unknown	3(2.5%)	2(3.5%)	1(4.8%)	0
**Risk analysis index category** n(%)				
Robust	89(75.4%)	47(82.5%)	19(90.5%)	86(81.1%)
Normal	17(14.4%)	8(14%)	1(4.8%)	9(8.5%)
Frail	11(9.3%)	2(3.5%)	0	9(8.5%)
Very Frail	1(0.8%)	0	1(4.8%)	2(1.9%)
**Surgical strata** n(%)				
No operation	9(7.6%)	1(1.8%)	2(9.5%)	8(7.5%)
Spine	31(26.3%)	14(24.6%)	3(14.3%)	34(32.1%)

General surgery/Surgical oncology	35(29.7%)	27(47.4%)	12(57.1%)	33(31.1%)
Colorectal surgery	12(10.2%)	10(17.5%)	1(4.8%)	13(12.3%)
Other	31(26.3%)	5(8.8%)	3(14.3%)	18(17%)
**Smoking status** n(%)				
Never	58(49.2%)	37(64.9%)	11(52.4%)	49(46.2%)
Passive	0	0	0	1(0.9%)
Quit	50(42.4%)	16(28.1%)	8(38.1%)	48(45.3%)
Yes	9(7.6%)	4(7%)	1(4.8%)	7(6.6%)
Unknown	1(0.8%)	0	1(4.8%)	1(0.9%)
BMI	30.8 ± 7.7	29.5 ± 5.3	29.5 ± 6.4	30.2 ± 6.4
**Coronary artery disease** n(%)				
No	102(86.4%)	48(84.2%)	20(95.2%)	91(85.8%)
Yes	16(13.6%)	9(15.8%)	1(4.8%)	15(14.2%)
**Cancer** n(%)				
No	77(65.3%)	47(82.5%)	18(85.7%)	73(68.9%)
Yes	41(34.7%)	10(17.5%)	3(14.3%)	33(31.1%)
**Congestive heart failure** n(%)				
No	115(97.5%)	57(100%)	21(100%)	104(98.1%)
Yes	3(2.5%)	0	0	2(1.9%)
**Chronic obstructive pulmonary disease** n(%)				
No	109(92.4%)	53(93%)	17(81%)	94(88.7%)
Yes	9(7.6%)	4(7%)	4(19%)	12(11.3%)
**Depression** n(%)				
No	95(80.5%)	47(82.5%)	18(85.7%)	81(76.4%)
Yes	23(19.5%)	10(17.5%)	3(14.3%)	25(23.6%)

**Deep vein thrombosis** n(%)				
No	113(95.8%)	55(96.5%)	20(95.2%)	104(98.1%)
Yes	5(4.2%)	2(3.5%)	1(4.8%)	2(1.9%)
**Osteoarthritis** n(%)				
No	98(83.1%)	47(82.5%)	17(81%)	88(83%)
Yes	20(16.9%)	10(17.5%)	4(19%)	18(17%)
**Pulmonary embolism** n(%)				
No	113(95.8%)	55(96.5%)	19(90.5%)	102(96.2%)
Yes	5(4.2%)	2(3.5%)	2(9.5%)	4(3.8%)
**Stroke** n(%)				
No	115(97.5%)	55(96.5%)	19(90.5%)	102(96.2%)
Yes	3(2.5%)	2(3.5%)	2(9.5%)	4(3.8%)
**Peripheral vascular disease** n(%)				
No	115(97.5%)	54(94.7%)	20(95.2%)	103(97.2%)
Yes	3(2.5%)	3(5.3%)	1(4.8%)	3(2.8%)
**Year of randomization** n(%)				
2019	18(15.3%)	3(5.3%)	2(9.5%)	13(12.3%)
2020	16(13.6%)	8(14%)	3(14.3%)	17(16%)
2021	68(57.6%)	37(64.9%)	13(61.9%)	62(58.5%)
2022	16(13.6%)	9(15.8%)	3(14.3%)	14(13.2%)

SUPPLEMENTAL TABLE 9. Intention-to-treat analysis groups' baseline characteristics and demographics by treatment duration

	Short duration	Intermediate duration	Long duration	Placebo
Age (mean±SD)	68.3 ± 6.9	69.2 ± 7.1	70.8 ± 5.7	67.8 ± 5.8
**Sex** n(%)				
Female	45(41.3%)	26(46.4%)	9(47.4%)	46(46.9%)
Male	64(58.7%)	30(53.6%)	10(52.6%)	52(53.1%)
**Race** n(%)				
Black	5(4.6%)	5(8.9%)	0	5(5.1%)
White	102(93.6%)	48(85.7%)	17(89.5%)	93(94.9%)
Other	1(0.9%)	2(3.6%)	0	0
Unknown	1(0.9%)	1(1.8%)	2(10.5%)	0
**Ethnicity** n(%)				
Not Hispanic or Latino	106(97.2%)	54(96.4%)	18(94.7%)	98(100%)
Hispanic or Latino	1(0.9%)	0	0	0
Unknown	2(1.8%)	2(3.6%)	1(5.3%)	0
**Risk analysis index category** n(%)				
Robust	81(74.3%)	47(83.9%)	17(89.5%)	80(81.6%)
Normal	16(14.7%)	7(12.5%)	1(5.3%)	8(8.2%)
Frail	11(10.1%)	2(3.6%)	0	9(9.2%)
Very Frail	1(0.9%)	0	1(5.3%)	1(1%)
**Surgical strata** n(%)				
Spine	31(28.4%)	14(25%)	3(15.8%)	34(34.7%)
General surgery/Surgical oncology	35(32.1%)	27(48.2%)	12(63.2%)	33(33.7%)

Colorectal surgery	12(11%)	10(17.9%)	1(5.3%)	13(13.3%)
Other	31(28.4%)	5(8.9%)	3(15.8%)	18(18.4%)
**Smoking status** n(%)				
Never	54(49.5%)	37(66.1%)	10(52.6%)	45(45.9%)
Passive	0	0	0	1(1%)
Quit	47(43.1%)	15(26.8%)	7(36.8%)	44(44.9%)
Yes	7(6.4%)	4(7.1%)	1(5.3%)	7(7.1%)
Unknown	1(0.9%)	0	1(5.3%)	1(1%)
**Body mass index** (mean±SD)	30.3 ± 5.6	29.4 ± 5.2	29.5 ± 6.7	29.9 ± 6.3
**Coronary artery disease** n(%)				
No	94(86.2%)	47(83.9%)	18(94.7%)	83(84.7%)
Yes	15(13.8%)	9(16.1%)	1(5.3%)	15(15.3%)
**Cancer** n(%)				
No	70(64.2%)	47(83.9%)	16(84.2%)	67(68.4%)
Yes	39(35.8%)	9(16.1%)	3(15.8%)	31(31.6%)
**Congestive heart failure** n(%)				
No	106(97.2%)	56(100%)	19(100%)	96(98%)
Yes	3(2.8%)	0	0	2(2%)
**Chronic obstructive pulmonary disease** n(%)				
No	101(92.7%)	52(92.9%)	16(84.2%)	86(87.8%)
Yes	8(7.3%)	4(7.1%)	3(15.8%)	12(12.2%)
**Depression** n(%)				
No	89(81.7%)	46(82.1%)	16(84.2%)	76(77.6%)
Yes	20(18.3%)	10(17.9%)	3(15.8%)	22(22.4%)
**Deep vein thrombosis** n(%)				

No	104(95.4%)	54(96.4%)	18(94.7%)	96(98%)
Yes	5(4.6%)	2(3.6%)	1(5.3%)	2(2%)
**Osteoarthritis** n(%)				
No	90(82.6%)	47(83.9%)	16(84.2%)	82(83.7%)
Yes	19(17.4%)	9(16.1%)	3(15.8%)	16(16.3%)
**Pulmonary embolism** n(%)				
No	104(95.4%)	54(96.4%)	17(89.5%)	96(98%)
Yes	5(4.6%)	2(3.6%)	2(10.5%)	2(2%)
**Stroke** n(%)				
No	106(97.2%)	54(96.4%)	17(89.5%)	94(95.9%)
Yes	3(2.8%)	2(3.6%)	2(10.5%)	4(4.1%)
**Peripheral vascular disease** n(%)				
No	106(97.2%)	53(94.6%)	18(94.7%)	95(96.9%)
Yes	3(2.8%)	3(5.4%)	1(5.3%)	3(3.1%)
**Year of randomization** n(%)				
2019	18(16.5%)	3(5.4%)	2(10.5%)	13(13.3%)
2020	14(12.8%)	8(14.3%)	2(10.5%)	16(16.3%)
2021	63(57.8%)	36(64.3%)	12(63.2%)	56(57.1%)
2022	14(12.8%)	9(16.1%)	3(15.8%)	13(13.3%)

SUPPLEMENTAL TABLE 10. Per-protocol analysis groups' baseline characteristics and demographics by treatment duration

	Short duration	Intermediate duration	Long duration	Placebo
**Age** (mean±SD)	67.4 ± 6.5	69.0 ± 7.1	70.3 ± 5.9	68.1 ± 5.8
**Sex** n(%)				
Female	35(39.8%)	21(46.7%)	7(46.7%)	40(48.8%)
Male	53(60.2%)	24(53.3%)	8(53.3%)	42(51.2%)
**Race** n(%)				
Black	5(5.7%)	4(8.9%)	0	4(4.9%)
White	81(92%)	38(84.4%)	13(86.7%)	78(95.1%)
Other	1(1.1%)	2(4.4%)	0	0
Unknown	1(1.1%)	1(2.2%)	2(13.3%)	0
**Ethnicity** n(%)				
Not Hispanic or Latino	86(97.7%)	43(95.6%)	14(93.3%)	82(100%)
Hispanic or Latino	1(1.1%)	0	0	0
Unknown	1(1.1%)	2(4.4%)	1(6.7%)	0
**Risk analysis index category** n(%)				
Robust	68(77.3%)	38(84.4%)	13(86.7%)	68(82.9%)
Normal	12(13.6%)	5(11.1%)	1(6.7%)	7(8.5%)
Frail	7(8%)	2(4.4%)	0	6(7.3%)
Very Frail	1(1.1%)	0	1(6.7%)	1(1.2%)
**Surgical strata** n(%)				
Spine	27(30.7%)	11(24.4%)	2(13.3%)	31(37.8%)
General surgery/Surgical oncology	27(30.7%)	24(53.3%)	11(73.3%)	27(32.9%)

Colorectal surgery	11(12.5%)	7(15.6%)	1(6.7%)	10(12.2%)
Other	23(26.1%)	3(6.7%)	1(6.7%)	14(17.1%)
**Smoking status** n(%)				
Never	42(47.7%)	31(68.9%)	6(40%)	43(52.4%)
Passive	0	0	0	1(1.2%)
Quit	38(43.2%)	12(26.7%)	7(46.7%)	35(42.7%)
Yes	7(8%)	2(4.4%)	1(6.7%)	3(3.7%)
Unknown	1(1.1%)	0	1(6.7%)	0
**Body mass index** (mean±SD)	30.2 ± 5.6	29.1 ± 4.5	30.5 ± 6.9	30.4 ± 6.6
**Coronary artery disease** n(%)				
No	76(86.4%)	38(84.4%)	14(93.3%)	69(84.1%)
Yes	12(13.6%)	7(15.6%)	1(6.7%)	13(15.9%)
**Cancer** n(%)				
No	58(65.9%)	36(80%)	14(93.3%)	59(72%)
Yes	30(34.1%)	9(20%)	1(6.7%)	23(28%)
**Congestive heart failure** n(%)				
No	87(98.9%)	45(100%)	15(100%)	81(98.8%)
Yes	1(1.1%)	0	0	1(1.2%)
**Chronic obstructive pulmonary disease** n(%)				
No	81(92%)	43(95.6%)	12(80%)	73(89%)
Yes	7(8%)	2(4.4%)	3(20%)	9(11%)
**Depression** n(%)				
No	73(83%)	38(84.4%)	13(86.7%)	64(78%)
Yes	15(17%)	7(15.6%)	2(13.3%)	18(22%)
**Deep vein thrombosis** n(%)				

No	84(95.5%)	43(95.6%)	14(93.3%)	80(97.6%)
Yes	4(4.5%)	2(4.4%)	1(6.7%)	2(2.4%)
**Osteoarthritis** n(%)				
No	72(81.8%)	38(84.4%)	14(93.3%)	68(82.9%)
Yes	16(18.2%)	7(15.6%)	1(6.7%)	14(17.1%)
**Pulmonary embolism** n(%)				
No	84(95.5%)	44(97.8%)	13(86.7%)	80(97.6%)
Yes	4(4.5%)	1(2.2%)	2(13.3%)	2(2.4%)
**Stroke** n(%)				
No	85(96.6%)	43(95.6%)	14(93.3%)	79(96.3%)
Yes	3(3.4%)	2(4.4%)	1(6.7%)	3(3.7%)
**Peripheral vascular disease** n(%)				
No	87(98.9%)	42(93.3%)	14(93.3%)	80(97.6%)
Yes	1(1.1%)	3(6.7%)	1(6.7%)	2(2.4%)
**Year of randomization** n(%)				
2019	12(13.6%)	1(2.2%)	1(6.7%)	10(12.2%)
2020	10(11.4%)	5(11.1%)	0	9(11%)
2021	53(60.2%)	30(66.7%)	11(73.3%)	50(61%)
2022	13(14.8%)	9(20%)	3(20%)	13(15.9%)

SUPPLEMENTAL TABLE 11. Overall cohorts' baseline characteristics and demographics by surgical strata

	No operation	Spine	General Surgery/Surgical oncology	Colorectal surgery	Other
**Age** (mean±SD)	66.8 ± 8.5	68.4 ± 5.9	69.1 ± 6.4	66.0 ± 5.8	68.9 ± 7.7
**Sex** n(%)					
Female	8(40%)	44(53.7%)	37(34.6%)	19(52.8%)	26(45.6%)
Male	12(60%)	38(46.3%)	70(65.4%)	17(47.2%)	31(54.4%)
**Race** n(%)					
Black	0	7(8.5%)	2(1.9%)	2(5.6%)	4(7%)
White	19(95%)	74(90.2%)	100(93.5%)	34(94.4%)	52(91.2%)
Other	1(5%)	1(1.2%)	2(1.9%)	0	0
Unknown	0	0	3(2.8%)	0	1(1.8%)
**Ethnicity** n(%)					
Not Hispanic or Latino	19(95%)	82(100%)	105(98.1%)	34(94.4%)	55(96.5%)
Hispanic or Latino	0	0	0	1(2.8%)	0
Unknown	1(5%)	0	2(1.9%)	1(2.8%)	2(3.5%)
**Risk analysis index category** n(%)					
Robust	16(80%)	74(90.2%)	83(77.6%)	26(72.2%)	42(73.7%)
Normal	3(15%)	5(6.1%)	13(12.1%)	7(19.4%)	7(12.3%)
Frail	0	3(3.7%)	10(9.3%)	2(5.6%)	7(12.3%)
Very Frail	1(5%)	0	1(0.9%)	1(2.8%)	1(1.8%)
**Smoking status** n(%)					
Never	9(45%)	41(50%)	59(55.1%)	19(52.8%)	27(47.4%)
Passive	0	1(1.2%)	0	0	0

Quit	9(45%)	31(37.8%)	43(40.2%)	15(41.7%)	24(42.1%)
Yes	2(10%)	9(11%)	3(2.8%)	2(5.6%)	5(8.8%)
Unknown	0	0	2(1.9%)	0	1(1.8%)
**Body mass index** (mean±SD)	34.7 ± 13.9	30.7 ± 5.2	29.5 ± 6.2	28.9 ± 5.7	30.4 ± 5.9
**Coronary artery disease** n(%)					
No	19(95%)	71(86.6%)	86(80.4%)	35(97.2%)	50(87.7%)
Yes	1(5%)	11(13.4%)	21(19.6%)	1(2.8%)	7(12.3%)
**Cancer** n(%)					
No	15(75%)	67(81.7%)	74(69.2%)	21(58.3%)	38(66.7%)
Yes	5(25%)	15(18.3%)	33(30.8%)	15(41.7%)	19(33.3%)
**Congestive heart failure** n(%)					
No	20(100%)	82(100%)	104(97.2%)	35(97.2%)	56(98.2%)
Yes	0	0	3(2.8%)	1(2.8%)	1(1.8%)
**Chronic obstructive pulmonary disease** n(%)					
No	18(90%)	76(92.7%)	97(90.7%)	32(88.9%)	50(87.7%)
Yes	2(10%)	6(7.3%)	10(9.3%)	4(11.1%)	7(12.3%)
**Depression** n(%)					
No	14(70%)	65(79.3%)	87(81.3%)	29(80.6%)	46(80.7%)
Yes	6(30%)	17(20.7%)	20(18.7%)	7(19.4%)	11(19.3%)
**Deep vein thrombosis** n(%)					
No	20(100%)	81(98.8%)	102(95.3%)	35(97.2%)	54(94.7%)
Yes	0	1(1.2%)	5(4.7%)	1(2.8%)	3(5.3%)
**Osteoarthritis** n(%)					
No	15(75%)	61(74.4%)	95(88.8%)	30(83.3%)	49(86%)
Yes	5(25%)	21(25.6%)	12(11.2%)	6(16.7%)	8(14%)

**Pulmonary embolism** n(%)					
No	18(90%)	77(93.9%)	104(97.2%)	35(97.2%)	55(96.5%)
Yes	2(10%)	5(6.1%)	3(2.8%)	1(2.8%)	2(3.5%)
**Stroke** n(%)					
No	20(100%)	76(92.7%)	105(98.1%)	34(94.4%)	56(98.2%)
Yes	0	6(7.3%)	2(1.9%)	2(5.6%)	1(1.8%)
**Peripheral vascular disease** n(%)					
No	20(100%)	78(95.1%)	103(96.3%)	35(97.2%)	56(98.2%)
Yes	0	4(4.9%)	4(3.7%)	1(2.8%)	1(1.8%)
**Year of randomization** n(%)					
2019	0	8(9.8%)	13(12.1%)	6(16.7%)	9(15.8%)
2020	4(20%)	9(11%)	17(15.9%)	5(13.9%)	9(15.8%)
2021	13(65%)	57(69.5%)	58(54.2%)	22(61.1%)	30(52.6%)
2022	3(15%)	8(9.8%)	19(17.8%)	3(8.3%)	9(15.8%)

SUPPLEMENTAL TABLE 12. Intention-to-treat analysis groups' baseline characteristics and demographics by surgical strata

	Spine	General Surgery/Surgical oncology	Colorectal surgery	Other
**Age** (mean±SD)	68.4 ± 5.9	69.1 ± 6.4	66.0 ± 5.8	68.9 ± 7.7
**Sex** n(%)				
Female	44(53.7%)	37(34.6%)	19(52.8%)	26(45.6%)
Male	38(46.3%)	70(65.4%)	17(47.2%)	31(54.4%)
**Race** n(%)				
Black	7(8.5%)	2(1.9%)	2(5.6%)	4(7%)
White	74(90.2%)	100(93.5%)	34(94.4%)	52(91.2%)
Other	1(1.2%)	2(1.9%)	0	0
Unknown	0	3(2.8%)	0	1(1.8%)
**Ethnicity** n(%)				
Not Hispanic or Latino	82(100%)	105(98.1%)	34(94.4%)	55(96.5%)
Hispanic or Latino	0	0	1(2.8%)	0
Unknown	0	2(1.9%)	1(2.8%)	2(3.5%)
**Risk analysis index category** n(%)				
Robust	74(90.2%)	83(77.6%)	26(72.2%)	42(73.7%)
Normal	5(6.1%)	13(12.1%)	7(19.4%)	7(12.3%)
Frail	3(3.7%)	10(9.3%)	2(5.6%)	7(12.3%)
Very Frail	0	1(0.9%)	1(2.8%)	1(1.8%)
**Smoking status** n(%)				
Never	41(50%)	59(55.1%)	19(52.8%)	27(47.4%)
Passive	1(1.2%)	0	0	0

Quit	31(37.8%)	43(40.2%)	15(41.7%)	24(42.1%)
Yes	9(11%)	3(2.8%)	2(5.6%)	5(8.8%)
Unknown	0	2(1.9%)	0	1(1.8%)
**Body mass index** (mean±SD)	30.7 ± 5.2	29.5 ± 6.2	28.9 ± 5.7	30.3 ± 5.9
**Coronary artery disease** n(%)				
No	71(86.6%)	86(80.4%)	35(97.2%)	50(87.7%)
Yes	11(13.4%)	21(19.6%)	1(2.8%)	7(12.3%)
**Cancer** n(%)				
No	67(81.7%)	74(69.2%)	21(58.3%)	38(66.7%)
Yes	15(18.3%)	33(30.8%)	15(41.7%)	19(33.3%)
**Congestive heart failure** n(%)				
No	82(100%)	104(97.2%)	35(97.2%)	56(98.2%)
Yes	0	3(2.8%)	1(2.8%)	1(1.8%)
**Chronic obstructive pulmonary disease** n(%)				
No	76(92.7%)	97(90.7%)	32(88.9%)	50(87.7%)
Yes	6(7.3%)	10(9.3%)	4(11.1%)	7(12.3%)
**Depression** n(%)				
No	65(79.3%)	87(81.3%)	29(80.6%)	46(80.7%)
Yes	17(20.7%)	20(18.7%)	7(19.4%)	11(19.3%)
**Deep vein thrombosis** n(%)				
No	81(98.8%)	102(95.3%)	35(97.2%)	54(94.7%)
Yes	1(1.2%)	5(4.7%)	1(2.8%)	3(5.3%)
**Osteoarthritis** n(%)				
No	61(74.4%)	95(88.8%)	30(83.3%)	49(86%)
Yes	21(25.6%)	12(11.2%)	6(16.7%)	8(14%)

**Pulmonary embolism** n(%)				
No	77(93.9%)	104(97.2%)	35(97.2%)	55(96.5%)
Yes	5(6.1%)	3(2.8%)	1(2.8%)	2(3.5%)
**Stroke** n(%)				
No	76(92.7%)	105(98.1%)	34(94.4%)	56(98.2%)
Yes	6(7.3%)	2(1.9%)	2(5.6%)	1(1.8%)
**Peripheral vascular disease** n(%)				
No	78(95.1%)	103(96.3%)	35(97.2%)	56(98.2%)
Yes	4(4.9%)	4(3.7%)	1(2.8%)	1(1.8%)
**Year of randomization** n(%)				
2019	8(9.8%)	13(12.1%)	6(16.7%)	9(15.8%)
2020	9(11%)	17(15.9%)	5(13.9%)	9(15.8%)
2021	57(69.5%)	58(54.2%)	22(61.1%)	30(52.6%)
2022	8(9.8%)	19(17.8%)	3(8.3%)	9(15.8%)

SUPPLEMENTAL TABLE 13. Per-protocol analysis groups' baseline characteristics and demographics by surgical strata

	Spine	General Surgery/Surgical oncology	Colorectal surgery	Other
**Age**(mean±SD)	68.2 ± 5.8	68.9 ± 6.3	65.7 ± 6.1	68.1 ± 7.2
**Sex** n(%)				
Female	37(52.1%)	30(33.7%)	15(51.7%)	21(51.2%)
Male	34(47.9%)	59(66.3%)	14(48.3%)	20(48.8%)
**Race** n(%)				
Black	5(7%)	2(2.2%)	2(6.9%)	4(9.8%)
White	65(91.5%)	82(92.1%)	27(93.1%)	36(87.8%)
Other	1(1.4%)	2(2.2%)	0	0
Unknown	0	3(3.4%)	0	1(2.4%)
**Ethnicity** n(%)				
Not Hispanic or Latino	71(100%)	87(97.8%)	27(93.1%)	40(97.6%)
Hispanic or Latino	0	0	1(3.4%)	0
Unknown	0	2(2.2%)	1(3.4%)	1(2.4%)
**Risk analysis index category** n(%)				
Robust	65(91.5%)	68(76.4%)	20(69%)	34(82.9%)
Normal	3(4.2%)	13(14.6%)	6(20.7%)	3(7.3%)
Frail	3(4.2%)	7(7.9%)	2(6.9%)	3(7.3%)
Very Frail	0	1(1.1%)	1(3.4%)	1(2.4%)
**Smoking status** n(%)				
Never	35(49.3%)	52(58.4%)	16(55.2%)	19(46.3%)
Passive	1(1.4%)	0	0	0

Quit	28(39.4%)	33(37.1%)	12(41.4%)	19(46.3%)
Yes	7(9.9%)	3(3.4%)	1(3.4%)	2(4.9%)
Unknown	0	1(1.1%)	0	1(2.4%)
**Body mass index** (mean±SD)	30.5± 5.2	29.7 ± 6.7	28.9 ± 5.5	31.1 ± .5.6
**Coronary artery disease** n(%)				
No	62(87.3%)	72(80.9%)	29(100%)	34(82.9%)
Yes	9(12.7%)	17(19.1%)	0	7(17.1%)
**Cancer** n(%)				
No	58(81.7%)	65(73%)	15(51.7%)	29(70.7%)
Yes	13(18.3%)	24(27%)	14(48.3%)	12(29.3%)
**Congestive heart failure** n(%)				
No	71(100%)	88(98.9%)	28(96.6%)	41(100%)
Yes	0	1(1.1%)	1(3.4%)	0
**Chronic obstructive pulmonary disease** n(%)				
No	66(93%)	79(88.8%)	26(89.7%)	38(92.7%)
Yes	5(7%)	10(11.2%)	3(10.3%)	3(7.3%)
**Depression** n(%)				
No	56(78.9%)	72(80.9%)	26(89.7%)	34(82.9%)
Yes	15(21.1%)	17(19.1%)	3(10.3%)	7(17.1%)
**Deep vein thrombosis** n(%)				
No	70(98.6%)	84(94.4%)	28(96.6%)	39(95.1%)
Yes	1(1.4%)	5(5.6%)	1(3.4%)	2(4.9%)
**Osteoarthritis** n(%)				
No	54(76.1%)	80(89.9%)	24(82.8%)	34(82.9%)
Yes	17(23.9%)	9(10.1%)	5(17.2%)	7(17.1%)

**Pulmonary embolism** n(%)				
No	66(93%)	87(97.8%)	28(96.6%)	40(97.6%)
Yes	5(7%)	2(2.2%)	1(3.4%)	1(2.4%)
**Stroke** n(%)				
No	66(93%)	88(98.9%)	27(93.1%)	40(97.6%)
Yes	5(7%)	1(1.1%)	2(6.9%)	1(2.4%)
**Peripheral vascular disease** n(%)				
No	68(95.8%)	87(97.8%)	28(96.6%)	40(97.6%)
Yes	3(4.2%)	2(2.2%)	1(3.4%)	1(2.4%)
**Year of randomization** n(%)				
2019	6(8.5%)	10(11.2%)	5(17.2%)	3(7.3%)
2020	7(9.9%)	12(13.5%)	2(6.9%)	3(7.3%)
2021	50(70.4%)	49(55.1%)	19(65.5%)	26(63.4%)
2022	8(11.3%)	18(20.2%)	3(10.3%)	9(22%)

SUPPLEMENTAL FIGURE 1. Horizontal stacked bar graph and forest plot displaying the distribution of 90-day hospital free days in the Per-protocol analysis

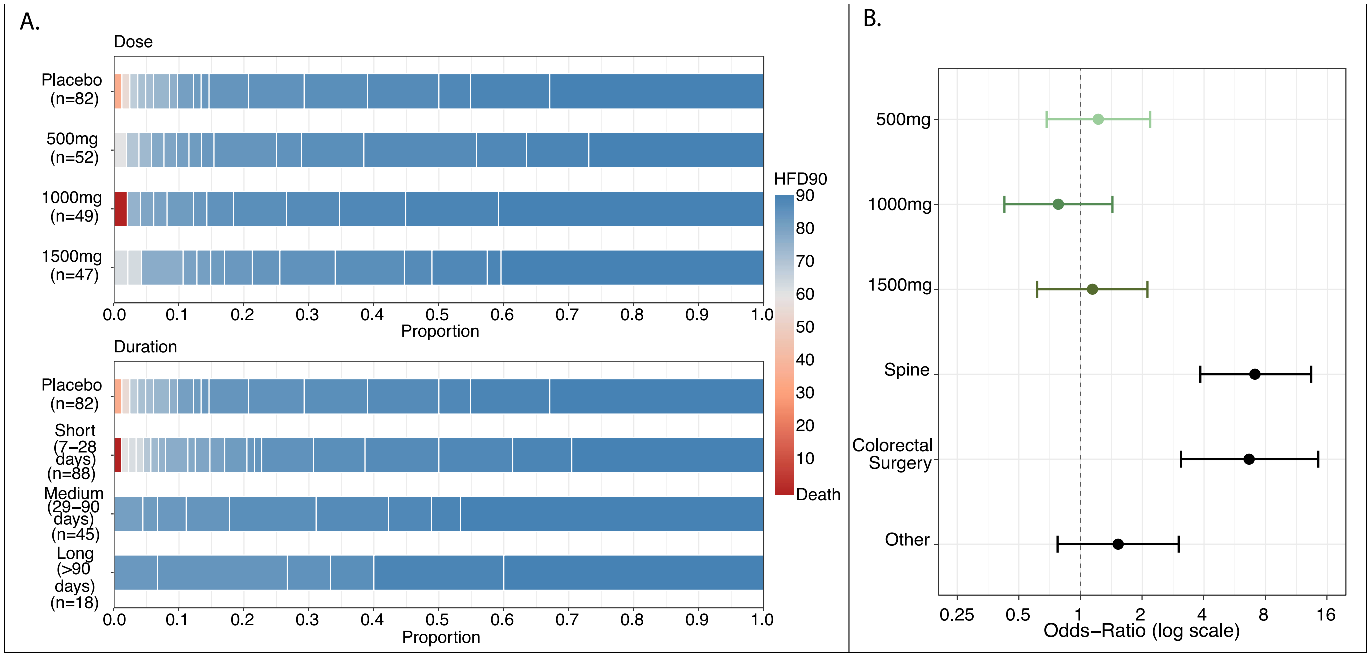

(A) Stacked proportion of post-discharge 90-day hospital free days for each intervention and duration. Red represents worse outcomes and blue represents better outcomes. (B) forest plot representing model-estimated odds-ratios. The dot is the posterior median and the line corresponds to the 95% credible interval.

SUPPLEMENTAL FIGURE 2. Bar graph displaying the rate of reoperation in the Intention-to-treat and Per-protocol analysis

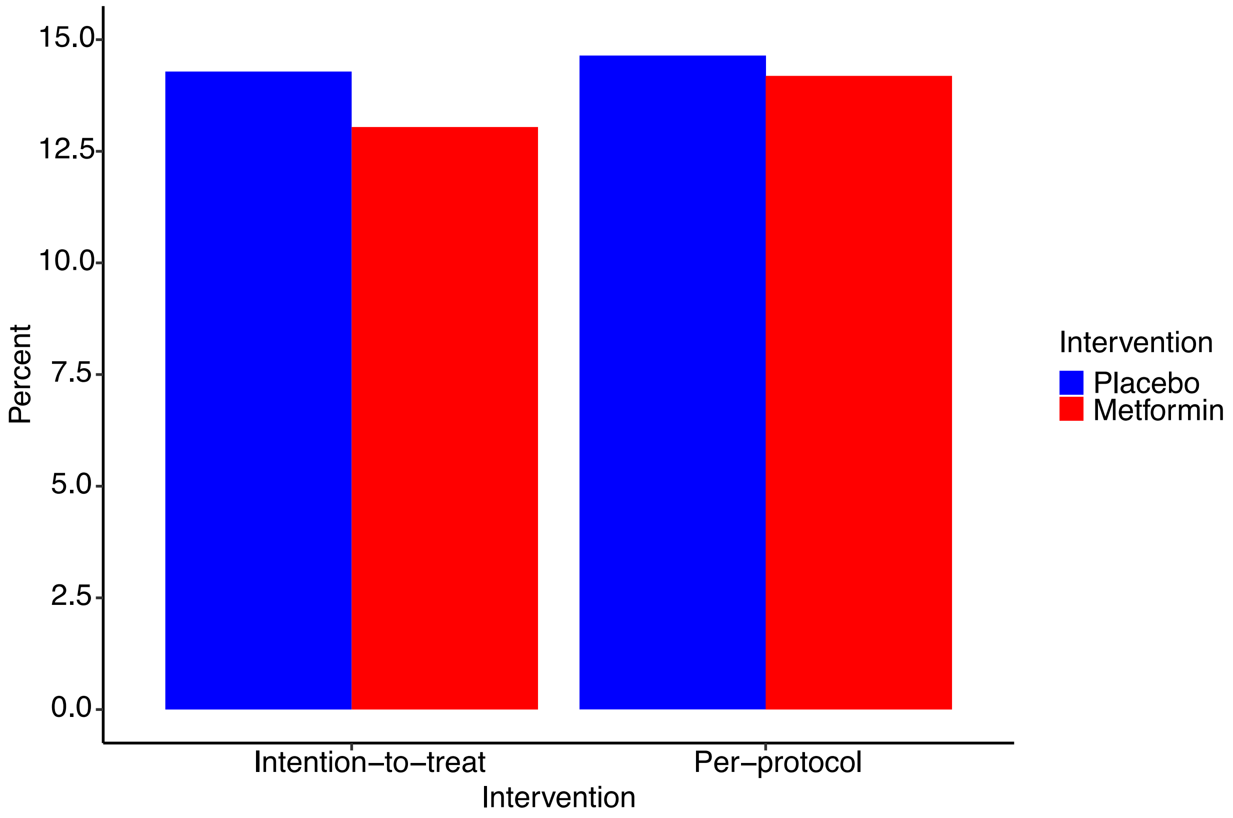

SUPPLEMENTAL FIGURE 3. Forest plot showing the results of the logistic regression model for reoperation and readmission in the Intention-to-treat analysis

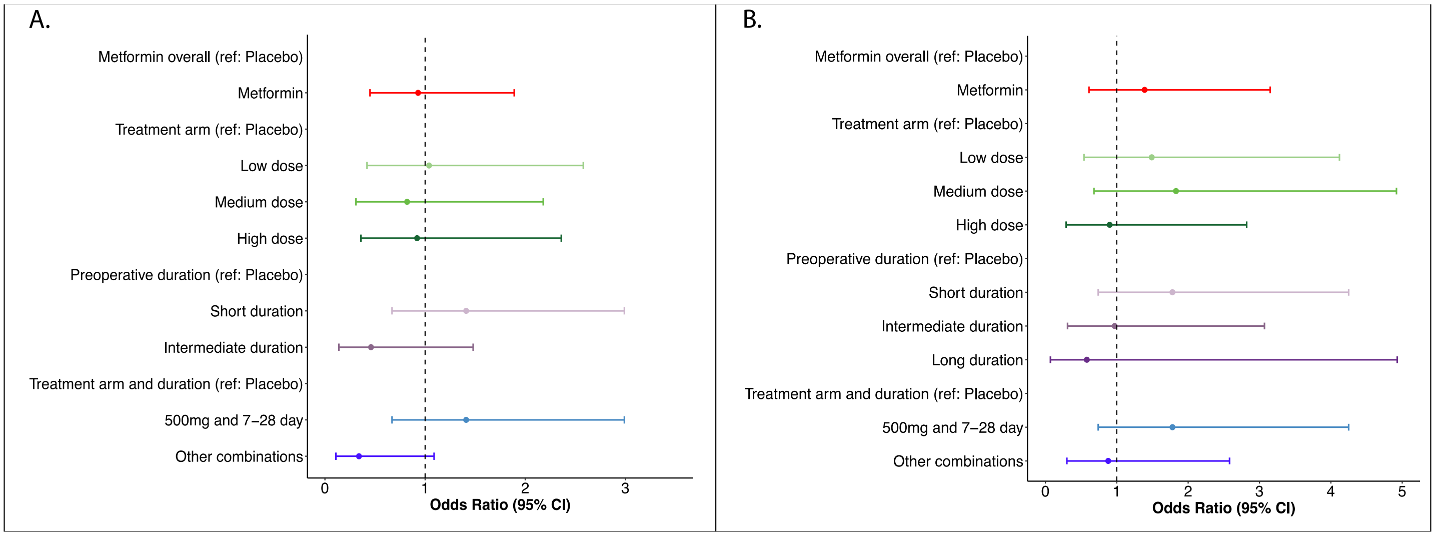

Panel A shows the results of the logistic regression model for reoperation. Long duration group was omitted because there were no reoperations in the group. Panel B shows the results of the logistic regression model for readmission.

SUPPLEMENTAL FIGURE 4. Bar graph displaying the rate of readmission in the Intention-to-treat and Per-protocol analysis

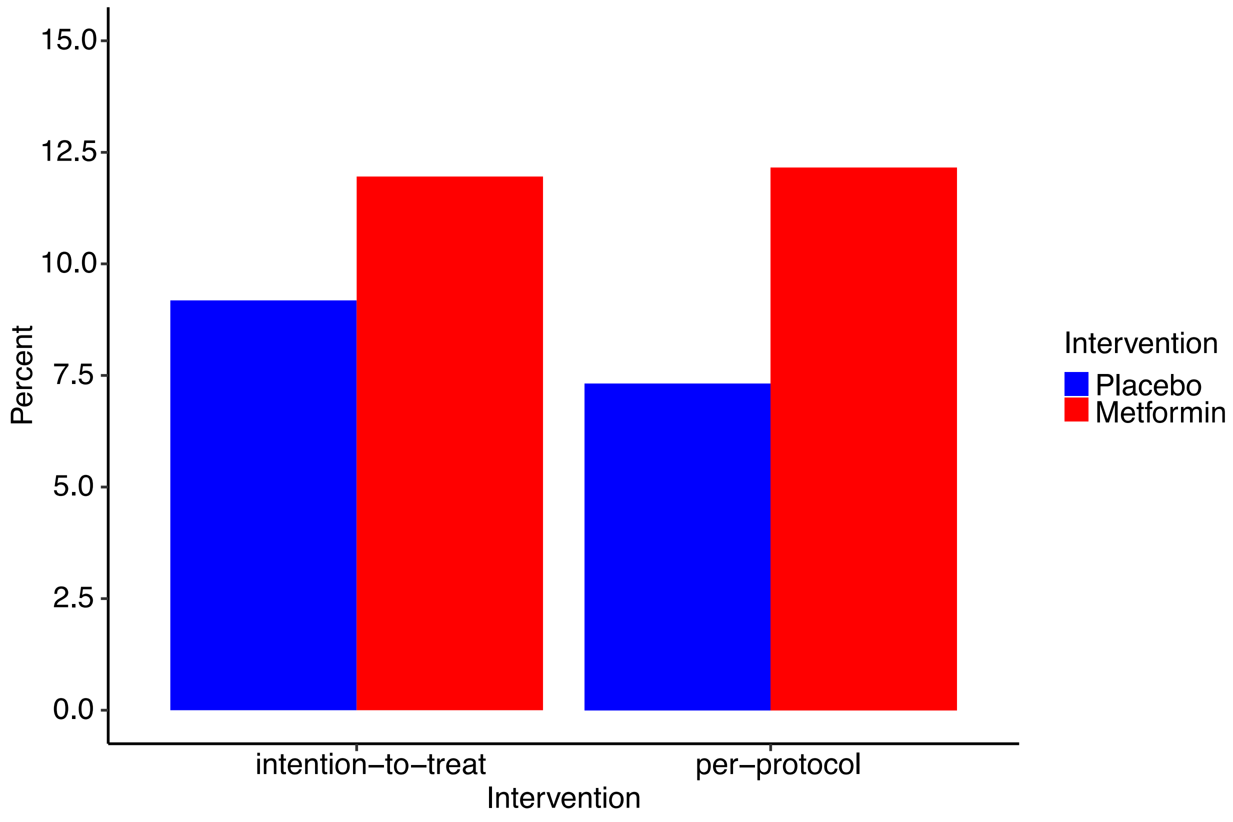

SUPPLEMENTAL FIGURE 5. Bar graph displaying the rate of 90-day adverse events in the Intention-to-treat and Per-protocol analysis

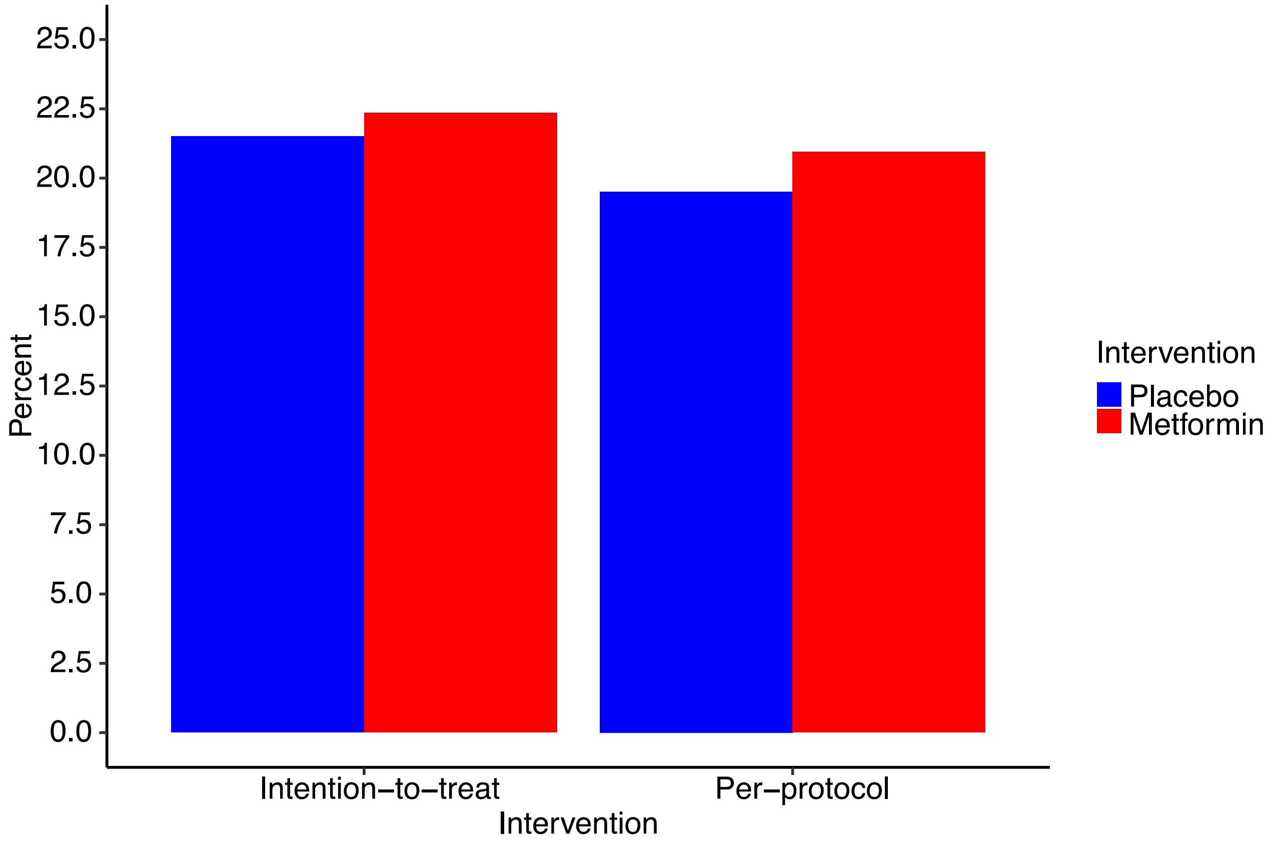

SUPPLEMENTAL FIGURE 6. Forest plot showing the results of the logistic regression for the 90-day adverse events in Intention-to-treat analysis

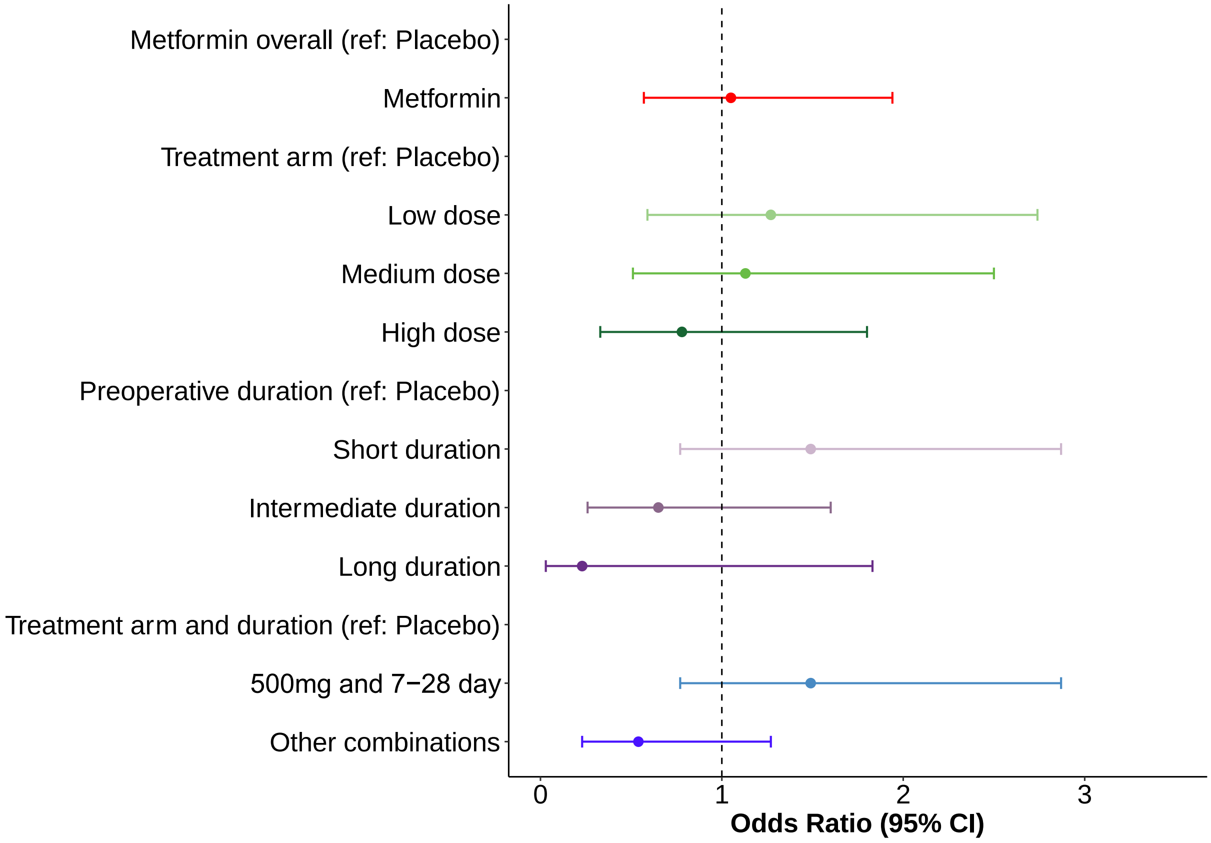

SUPPLEMENTAL FIGURE 7. Forest plot showing the results of the logistic regression for the rate of reoperation and readmission in Per-protocol analysis

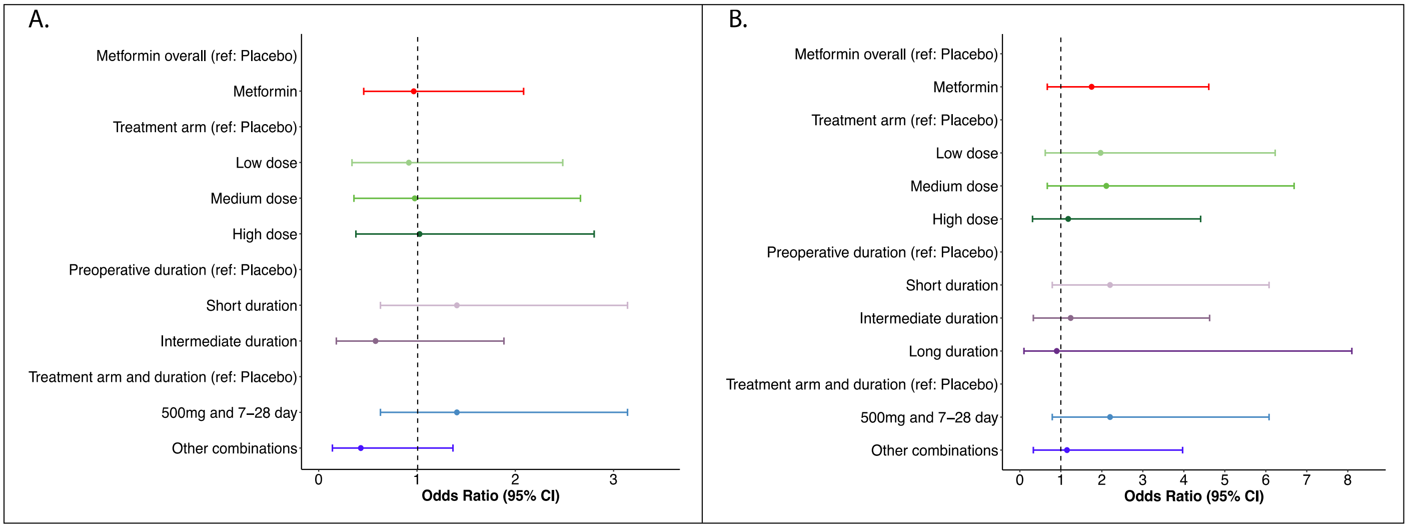

Panel A shows the results of the logistic regression model for reoperation. Long duration group was omitted because there were no reoperations in the group. Panel B shows the results of the logistic regression model for readmission.

SUPPLEMENTAL FIGURE 8. Kaplan-Meier curves and forest plot showing the rate of event-free survival and results of cox regression for the 90-day reoperation and readmission in the Per-protocol analysis

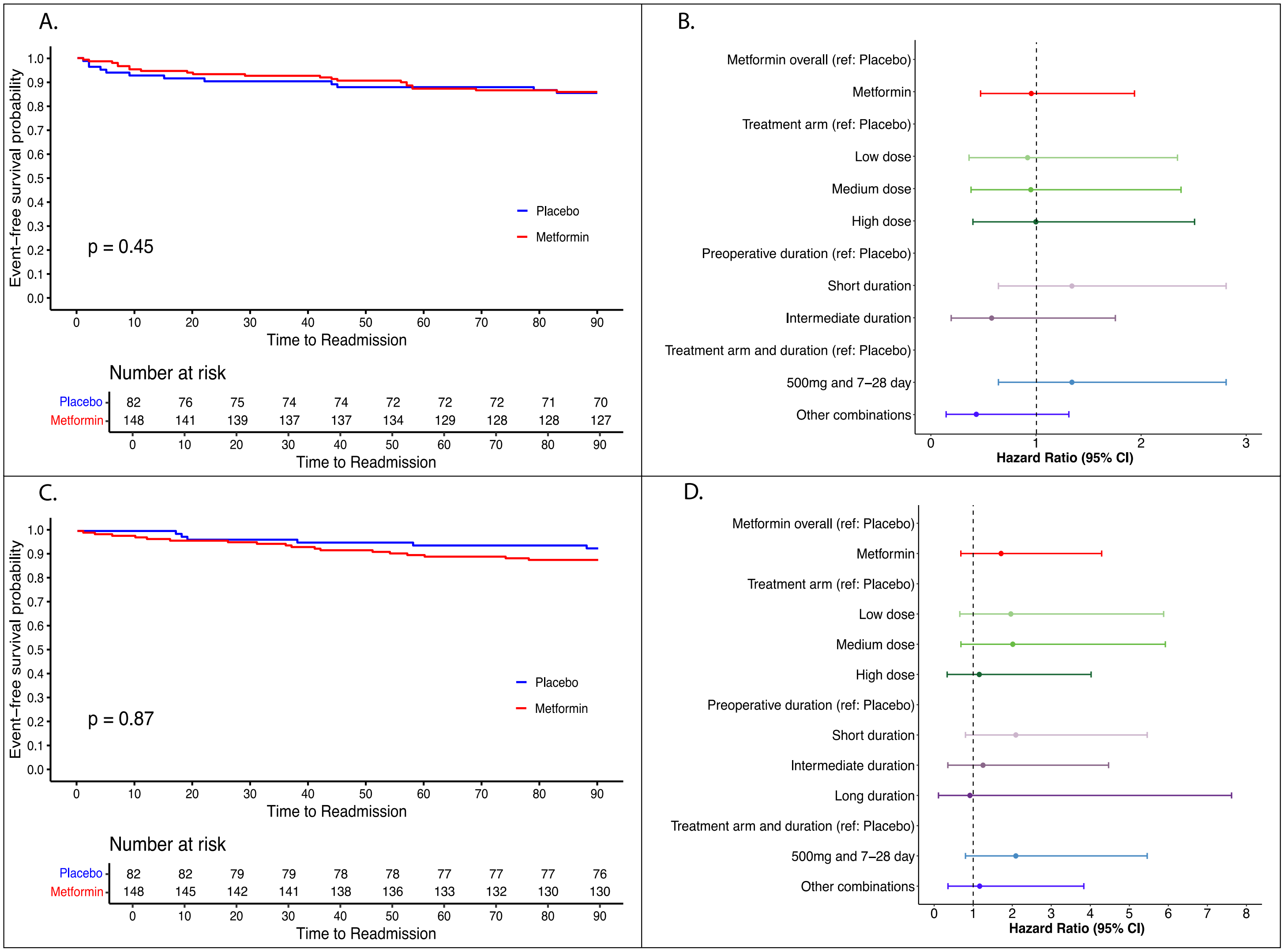

(A) Kaplan-Meier curves representing the rate of a reoperation within 90 days of the initial operation. (B) forest plot representing the hazard ratios and 95% CI for primary and sensitivity analysis for the 90-day reoperation. (A) Kaplan-Meier curves representing the rate of a readmission within 90 days of the initial operation, no events occurred for long duration and was not included in the forest plot. (B) forest plot representing the hazard ratios and 95% CI for primary and sensitivity analysis for the 90-day readmission.

SUPPLEMENTAL FIGURE 9. Kaplan-Meier curves and forest plot showing the rate of event-free survival and results of cox regression for the 90-day adverse events in the Per-protocol analysis

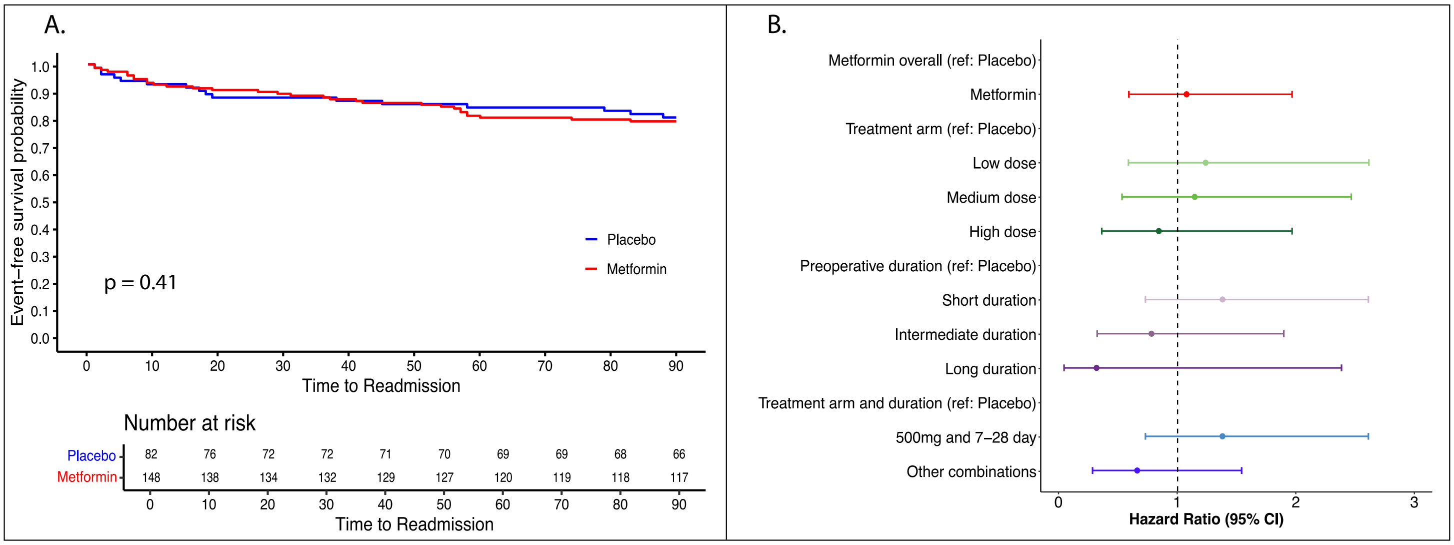

(A) Kaplan-Meier curves representing the rate of occurrence of an adverse event within 90 days of the operation. 19 participants that withdrew did not have complete information and were excluded. (B) forest plot representing the hazard ratios and 95% CI for primary and sensitivity analysis for the 90-day adverse events.

SUPPLEMENTAL FIGURE 10. Forest plot showing the results of the Bayesian sensitivity analysis in the Intention-to-treat and Per-protocol analysis

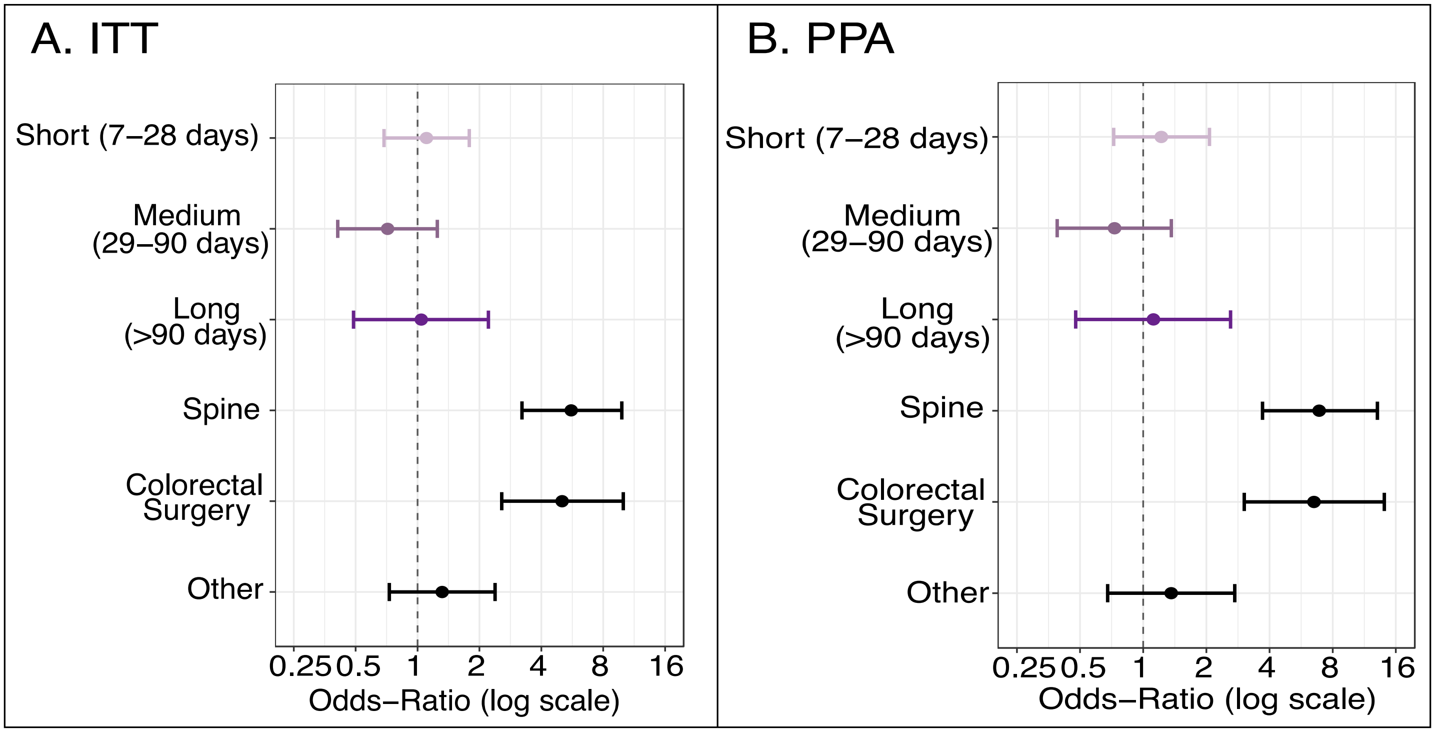

ITT: intention-to-treat, PPA: Per protocol

SUPPLEMENTAL FIGURE 11. Forest plot showing the results of the frequentist sensitivity analysis of primary outcome in the Intention-to-treat analysis

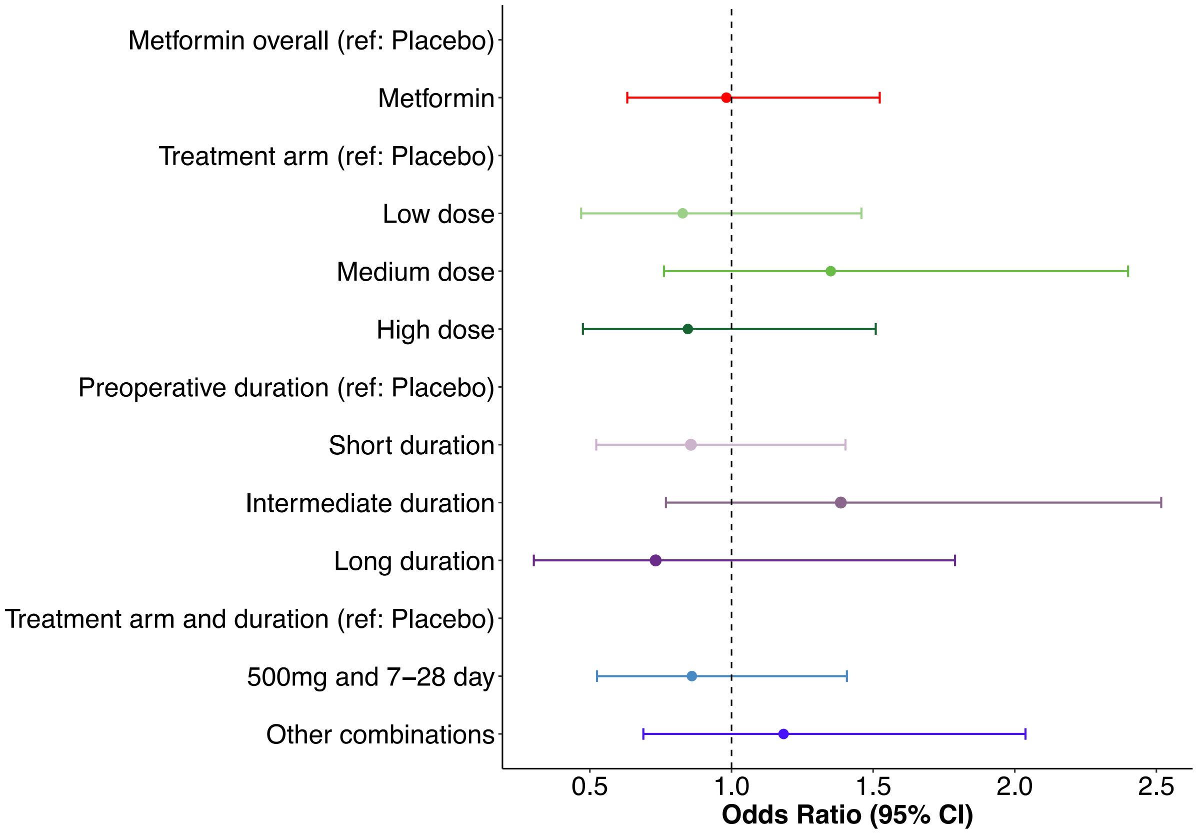

SUPPLEMENTAL FIGURE 12. Forest plot showing the results of the frequentist sensitivity analysis of primary outcome in the Per-protocol analysis

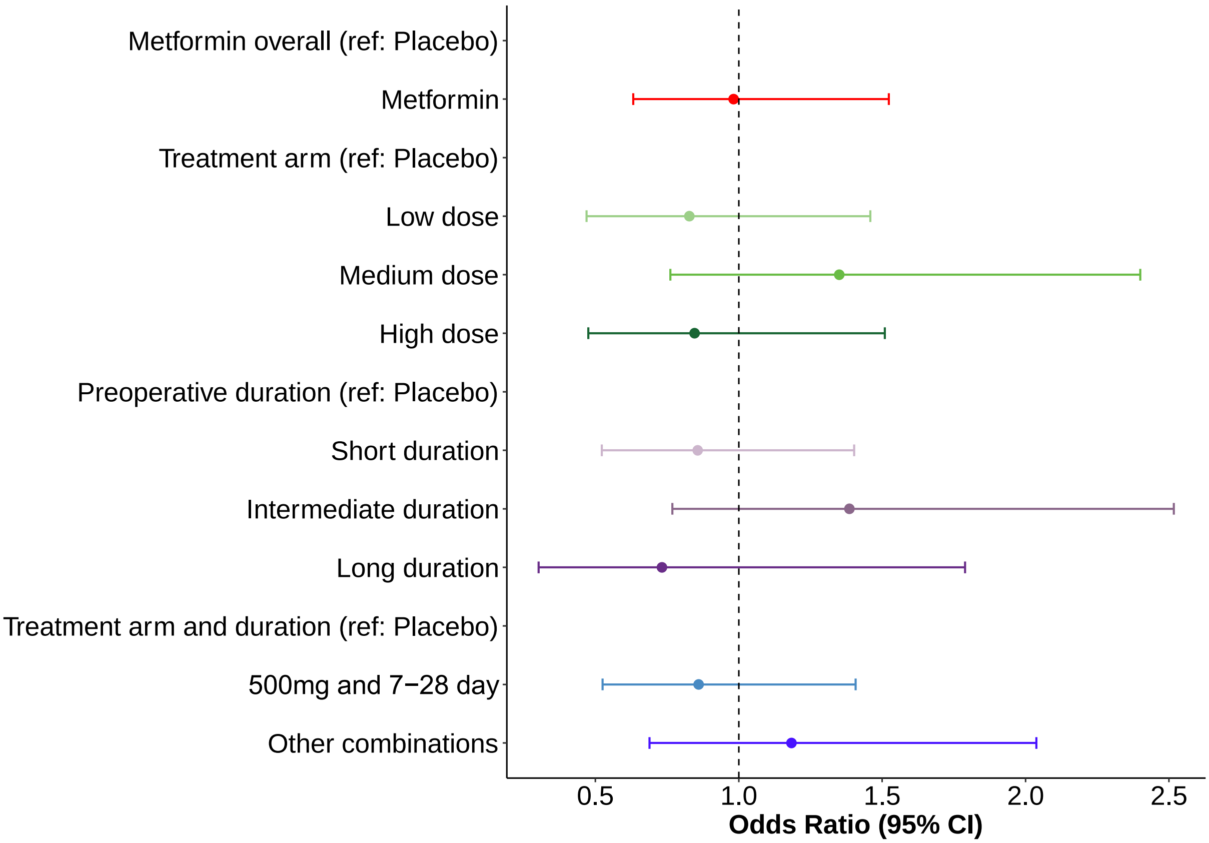

SUPPLEMENTAL FIGURE 13. Forest plot showing the results of the age specific subgroup analysis in both the Intention-to-treat and Per-protocol analysis

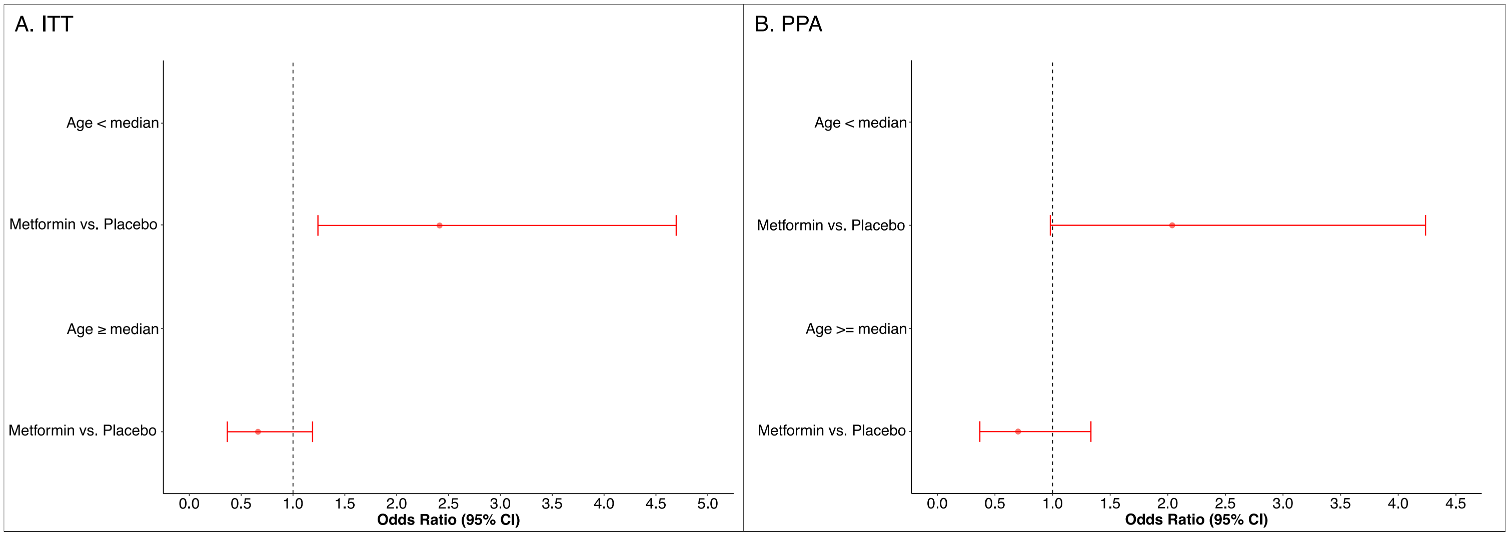

ITT: intention-to-treat, PPA: Per protocol

SUPPLEMENTAL FIGURE 14. Forest plot showing the results of the sex specific subgroup analysis in both the Intention-to-treat and Per-protocol analysis

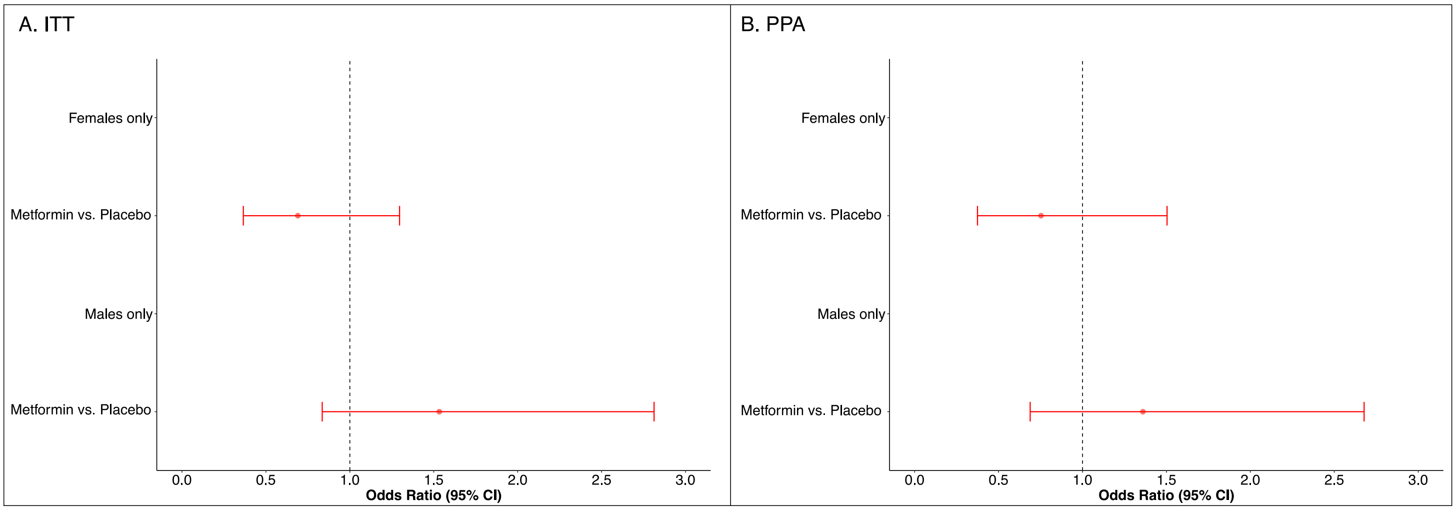

ITT: intention-to-treat, PPA: Per protocol

SUPPLEMENTAL FIGURE 15. Forest plot showing the results of the frailty specific subgroup analysis in both the Intention-to-treat and Per-protocol analysis

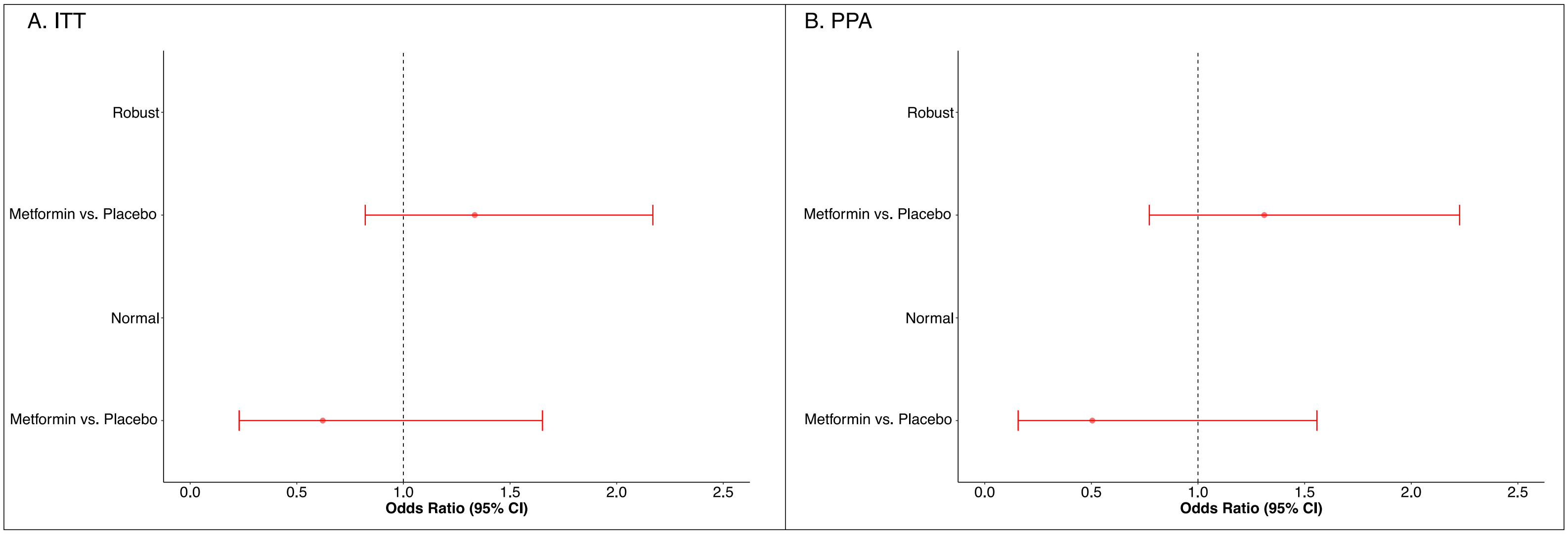

ITT: intention-to-treat, PPA: Per protocol

SUPPLEMENTAL FIGURE 16. Forest plot showing the results of the surgical strata specific subgroup analysis in both the Intention-to-treat and Per-protocol analysis

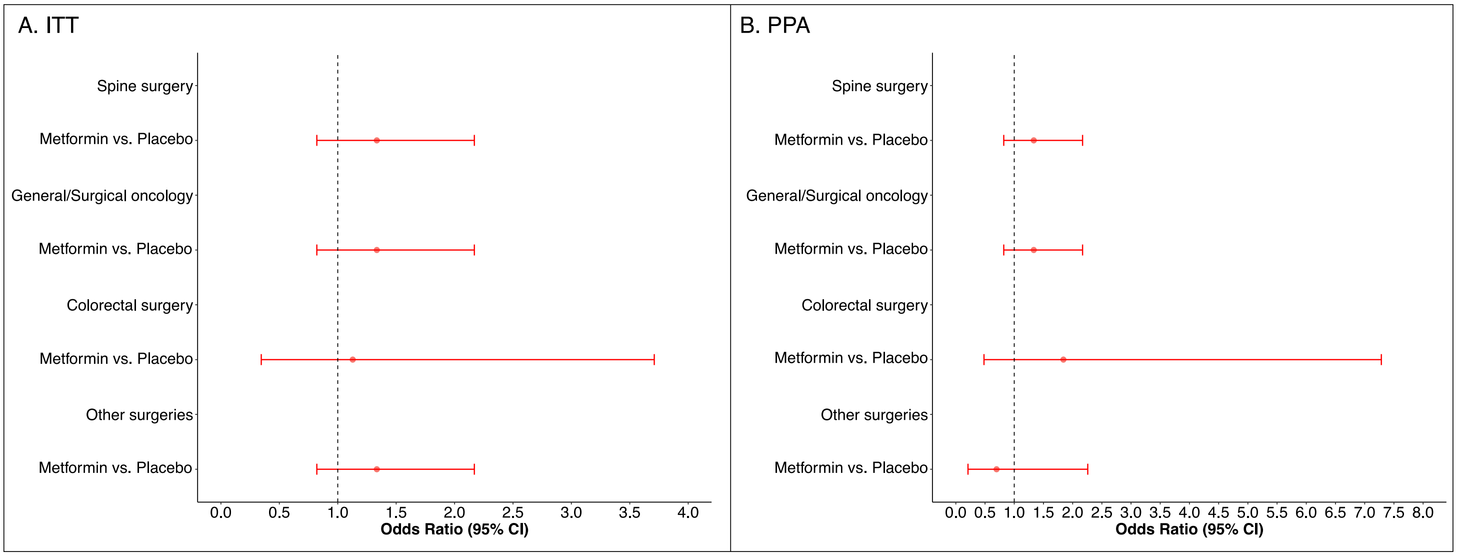

ITT: intention-to-treat, PPA: Per protocol

SUPPLEMENTAL FIGURE 17. Sensitivity analysis of the dichotomized categories of HFD-90 in the Intention-to-treat

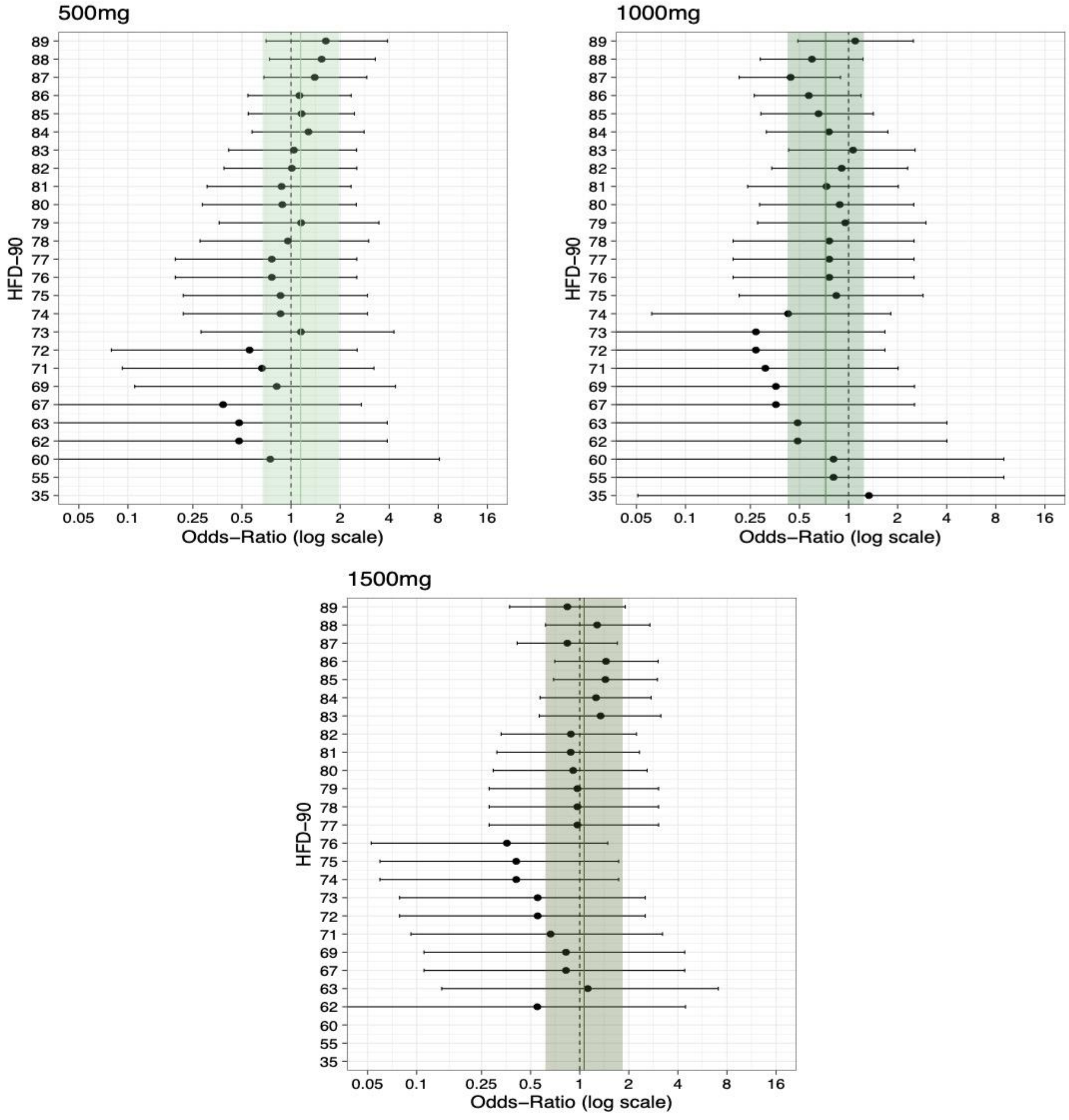

analysis population by active dose

Each dot represents the odds ratio estimate for the dichotomized endpoint using the cut point indicated on the y-axis (where the dichotomization is ≤ cut point versus > cut point). The horizontal lines represent the 95% credible intervals of the odds ratio estimate for the dichotomized endpoint. These estimates are compared to the common odds ratio from the

primary analysis where the solid vertical line is the posterior mean, and the shaded region is the 95% credible interval. The dashed vertical lines show, for reference, an odds ratio of 1.0 (black). Missing points are outside of the bounds of the axis.

SUPPLEMENTAL FIGURE 18. Sensitivity analysis of the dichotomized categories of HFD-90 in the Intention-to-treat analysis population by active duration

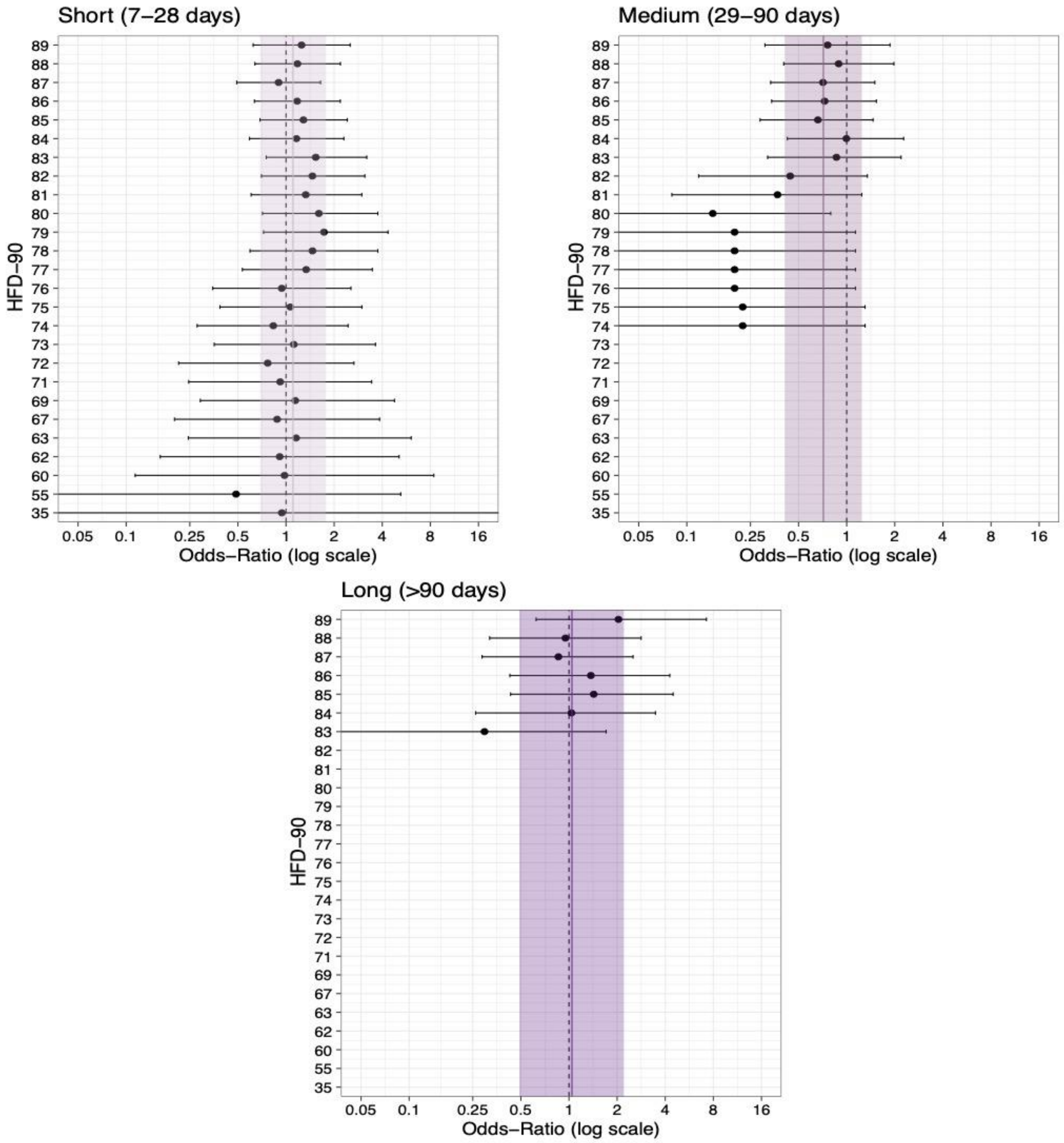

Each dot represents the odds ratio estimate for the dichotomized endpoint using the cut point indicated on the y-axis (where the dichotomization is ≤ cut point versus > cut point). The horizontal lines represent the 95% credible intervals of the odds ratio estimate for the dichotomized endpoint. These estimates are compared to the common odds ratio from the

primary analysis where the solid vertical line is the posterior mean, and the shaded region is the 95% credible interval. The dashed vertical lines show, for reference, an odds ratio of 1.0 (black). Missing points are outside of the bounds of the axis.

SUPPLEMENTAL FIGURE 19. Bar graph showing the rate of ICU (intensive care unit) admission by intervention arm in both the Intention-to-treat and Per-protocol analysis

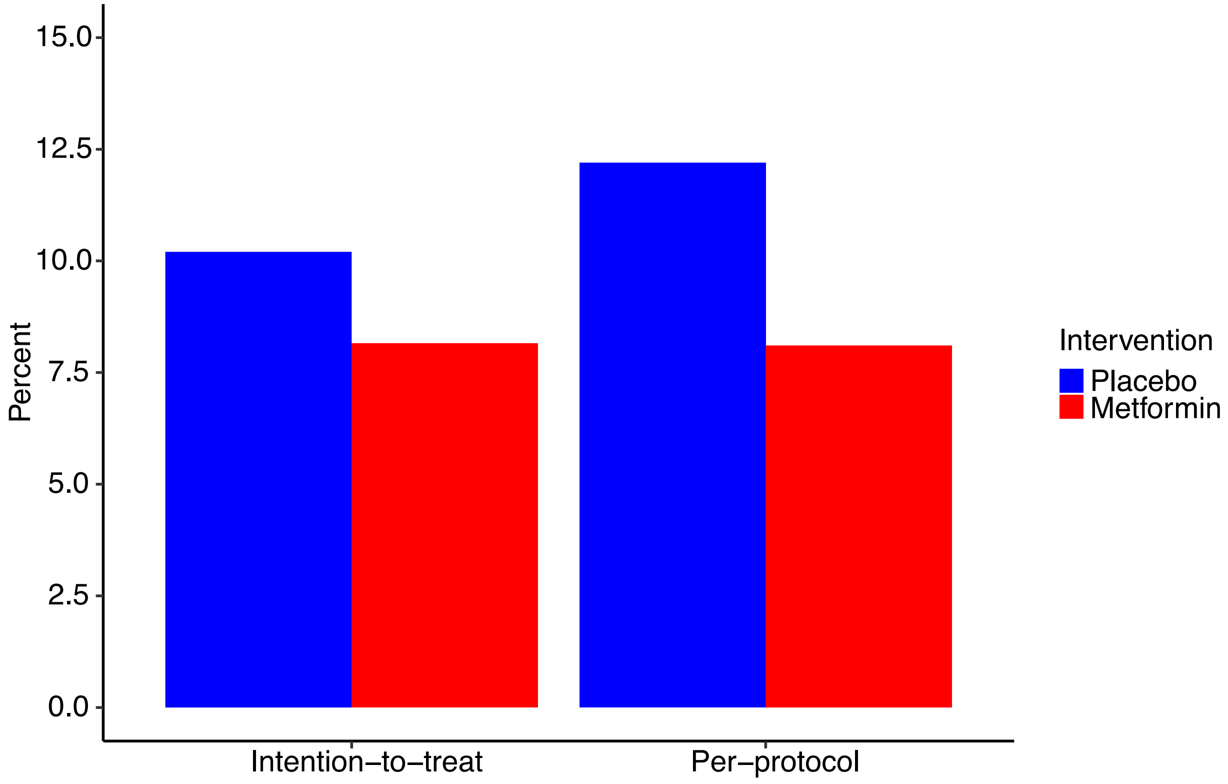

SUPPLEMENTAL FIGURE 20. Forest plot showing the results of the logistic regression for the rate of ICU (intensive care unit) admission in Intention-to-treat and Per-protocol analysis

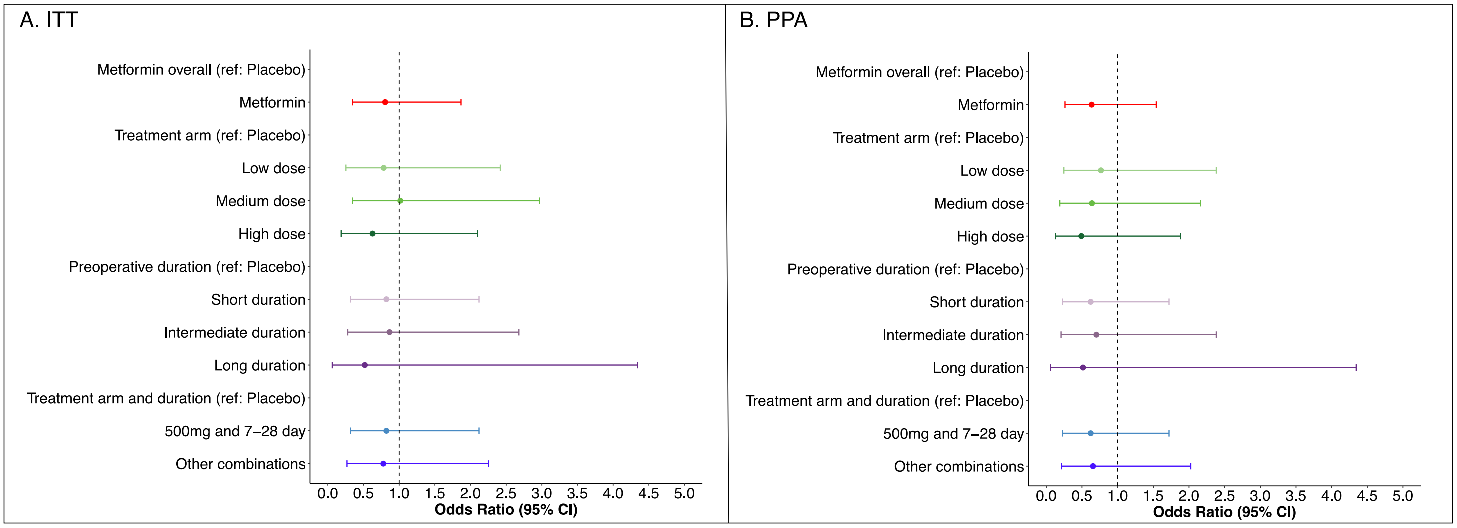

ITT: intention-to-treat, PPA: Per protocol

SUPPLEMENTAL FIGURE 21. Box and whisker plot showing the length of postoperative ICU (intensive care unit) stay by intervention arm in both the Intention-to-treat and Per-protocol analysis

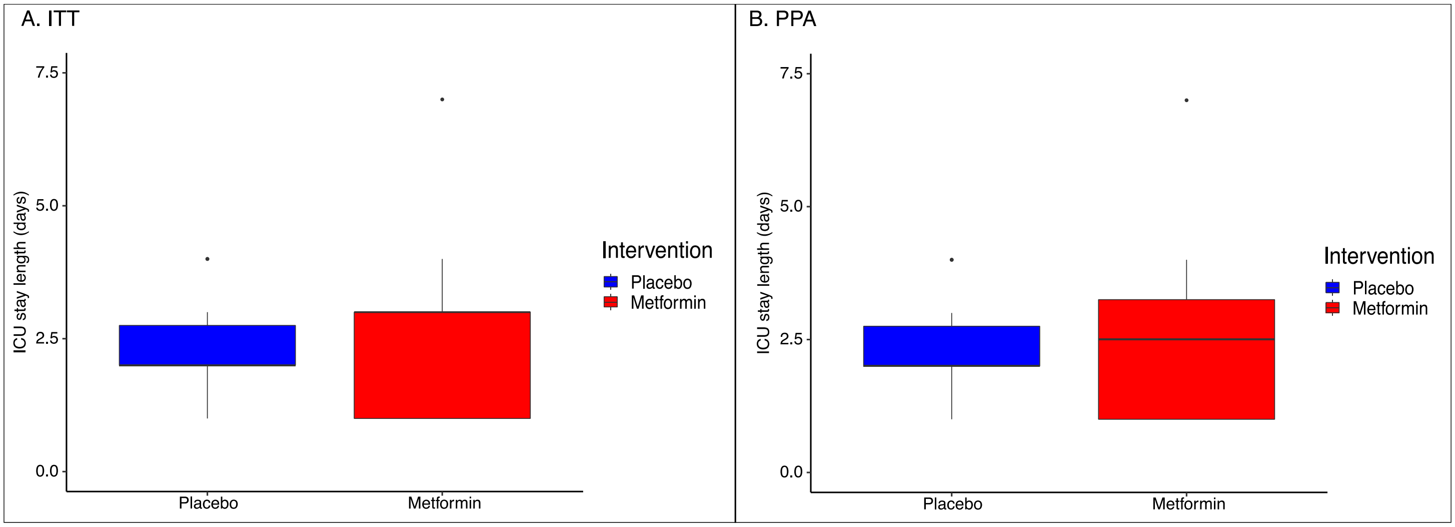

ITT: intention-to-treat, PPA: Per protocol

SUPPLEMENTAL FIGURE 22. Box and whisker plot showing the length of postoperative hospital stay by intervention arm in both the Intention-to-treat and Per-protocol analysis

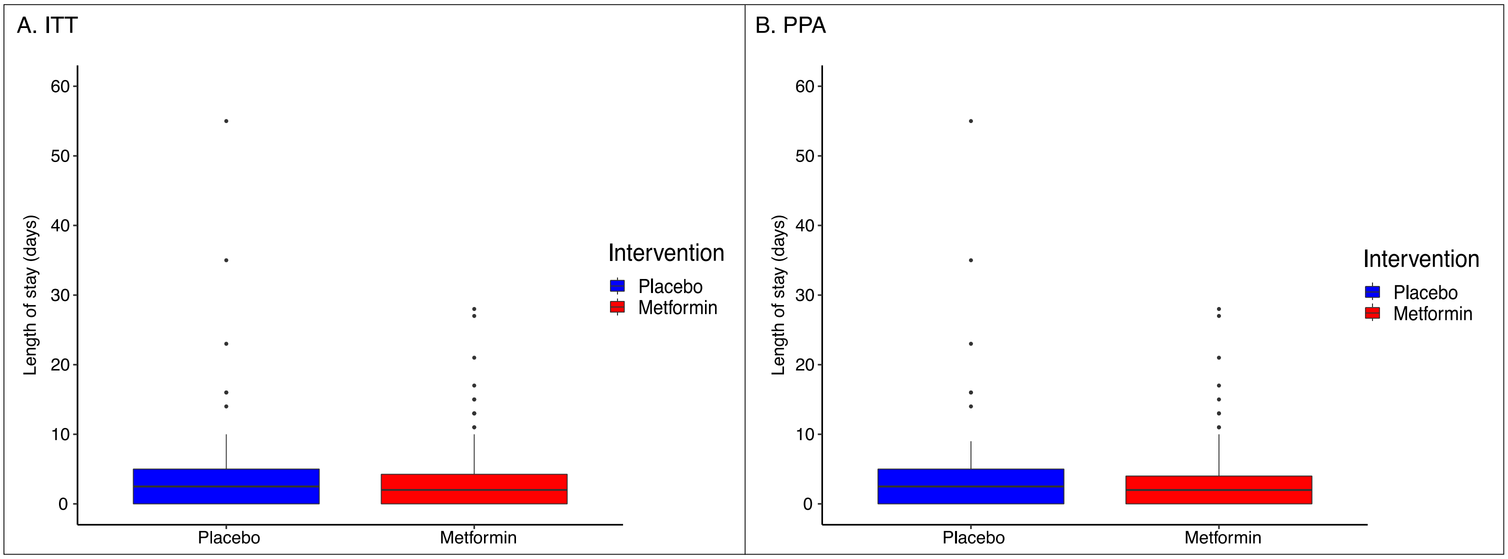

ITT: intention-to-treat, PPA: Per protocol

SUPPLEMENTAL FIGURE 23. Bar graph showing the rates of discharge destination by intervention arm in both the Intention-to-treat and Per-protocol analysis

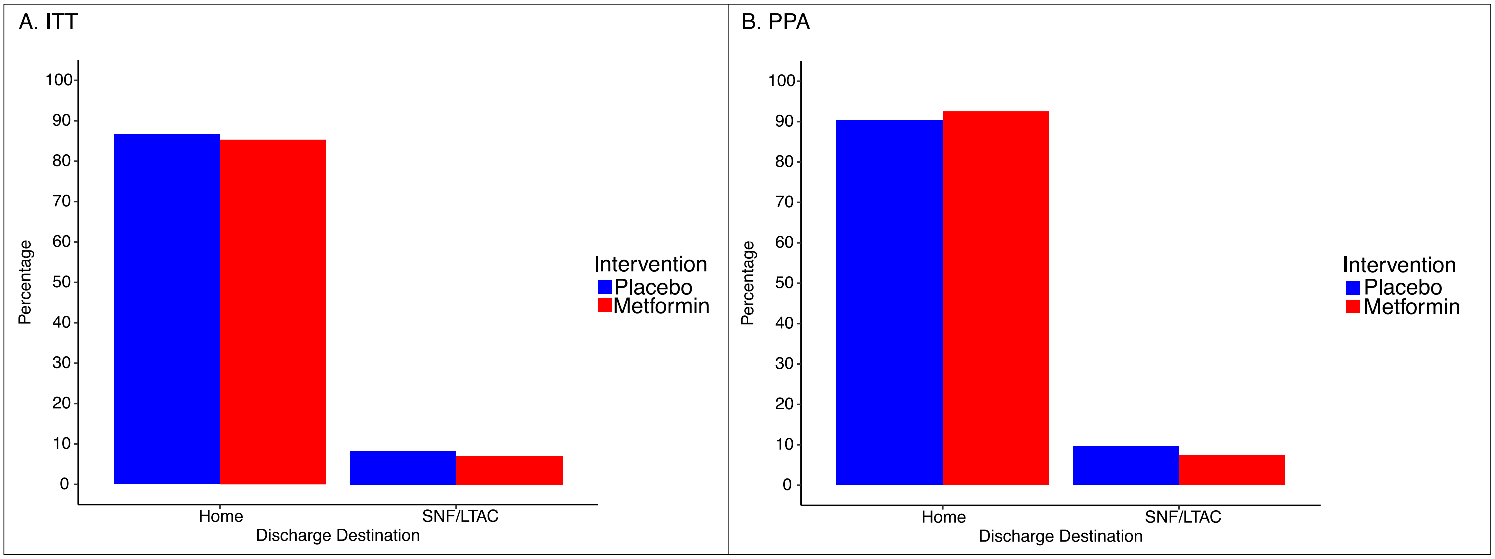

ITT: intention-to-treat, PPA: Per protocol

SUPPLEMENTAL FIGURE 24. Forest plot showing the results of the logistic regression for the odds of being discharged to a SNF/LTAC in the Intention-to-treat and Per-protocol analysis

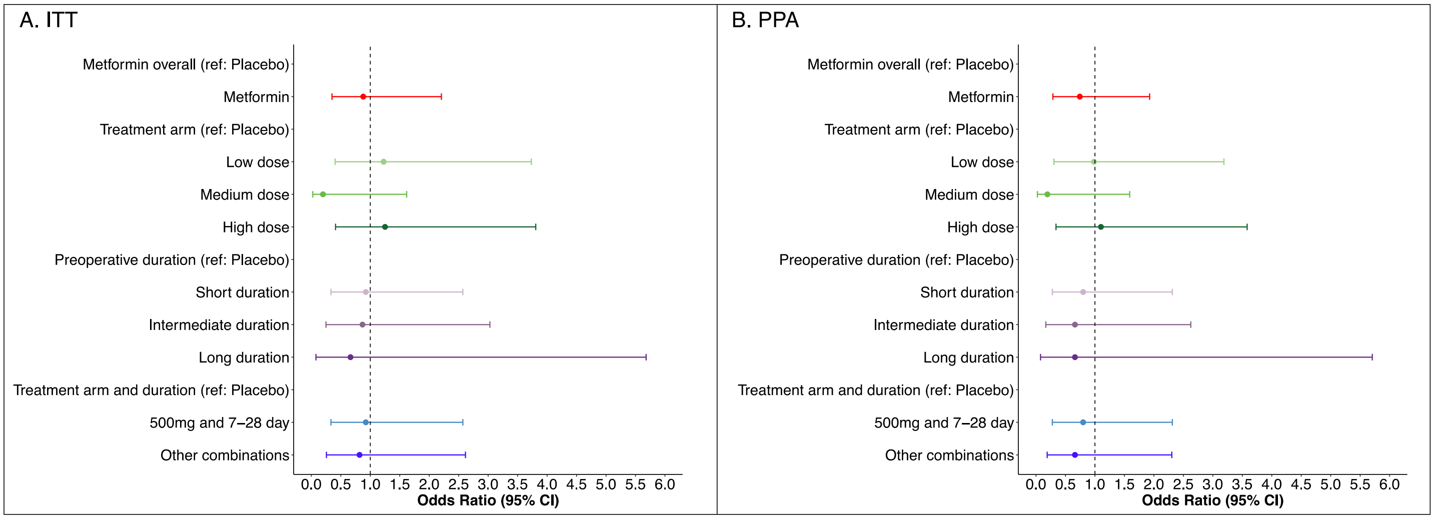

ITT: intention-to-treat, PPA: Per protocol

SUPPLEMENTAL FIGURE 25. Box and whisker plot showing the distribution of 30-day organ failure free days in both Intention-to-treat and Per-protocol analysis

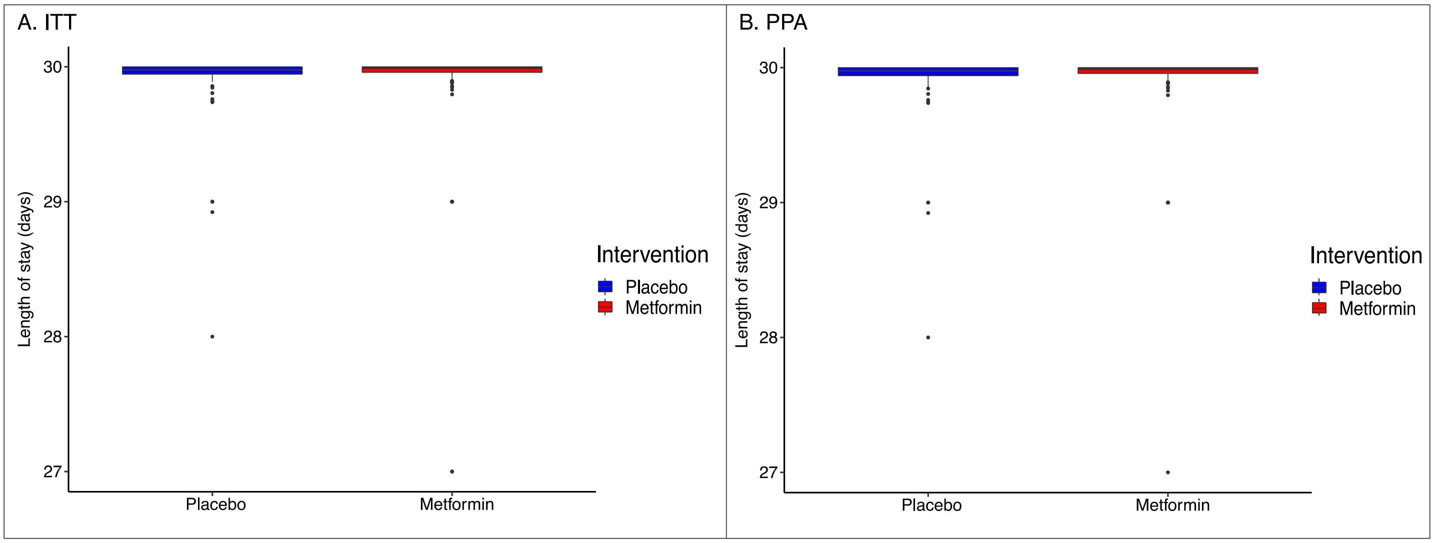

ITT: intention-to-treat, PPA: Per protocol

SUPPLEMENTAL FIGURE 26. Bar graph showing the rate of surgical site infection according to the VASQUIP definition by intervention arm in both the Intention-to-treat and Per-protocol analysis

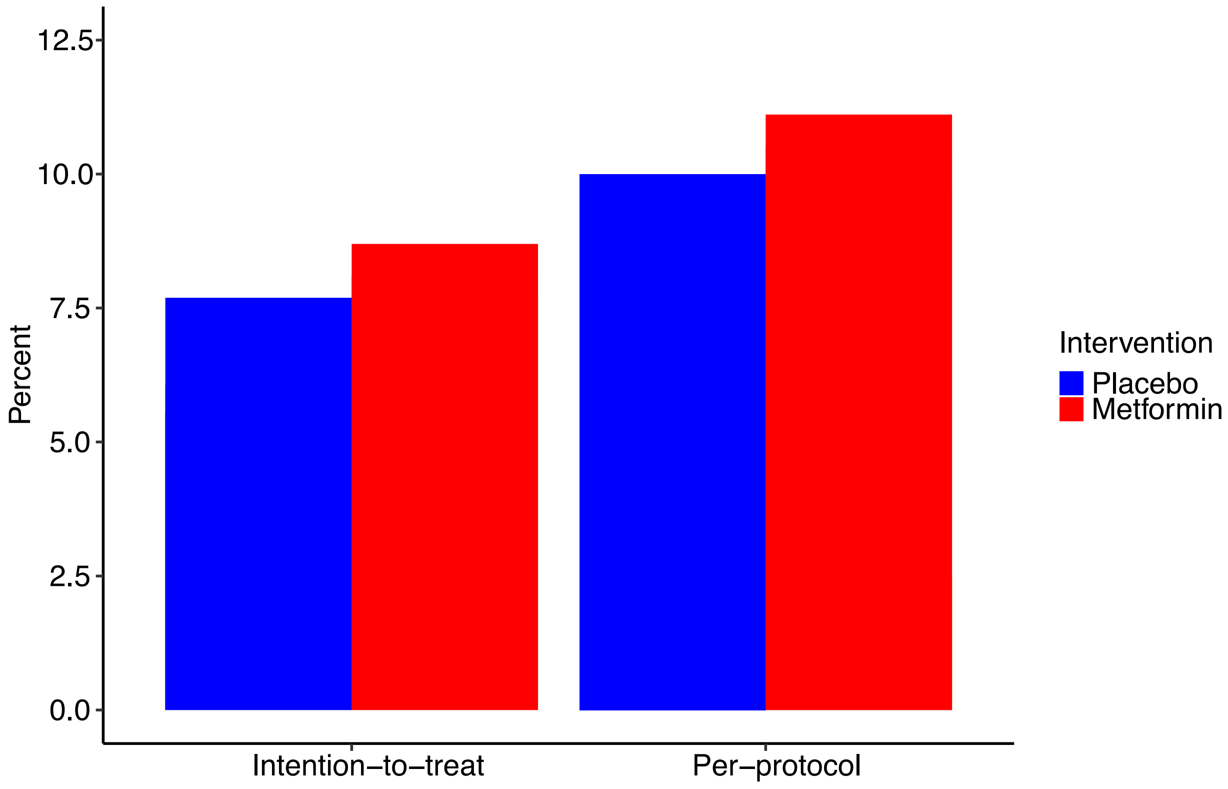

SUPPLEMENTAL FIGURE 27. Forest plot showing the results of the logistic regression for the odds of having a surgical site infection according to the VASQUIP definition in the Intention-to-treat and Per-protocol analysis

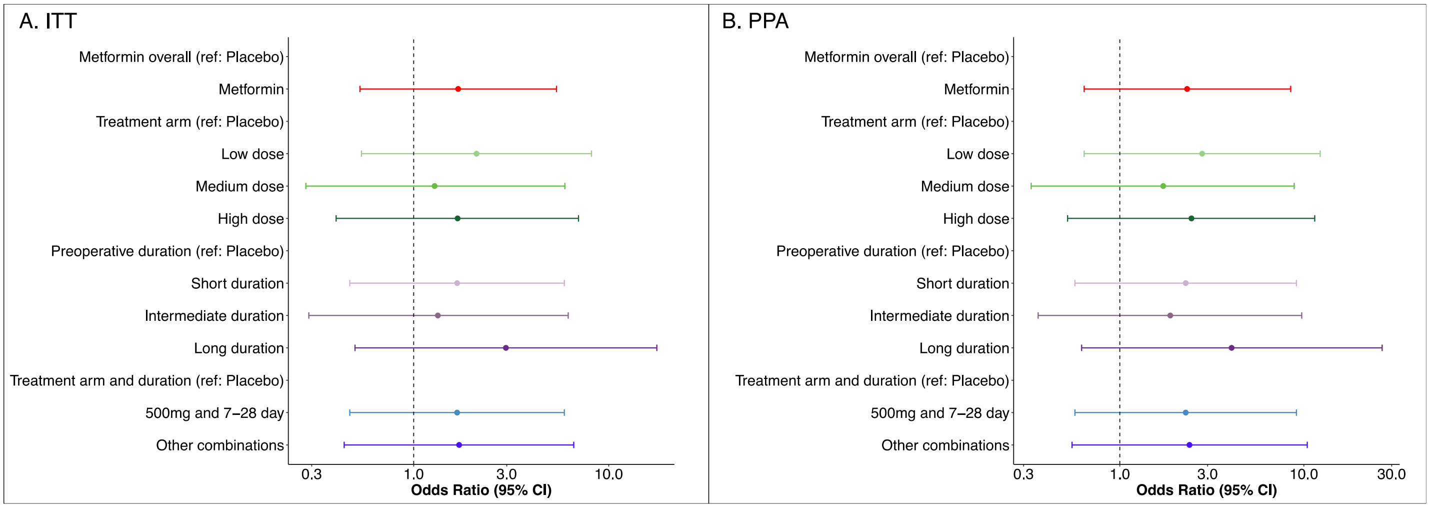

ITT: intention-to-treat, PPA: Per protocol

x-axis is in log scale

SUPPLEMENTAL FIGURE 28. Bar graph showing the rate of surgical site infection according to the ventral hernia working group definition by intervention arm in both the Intention-to-treat

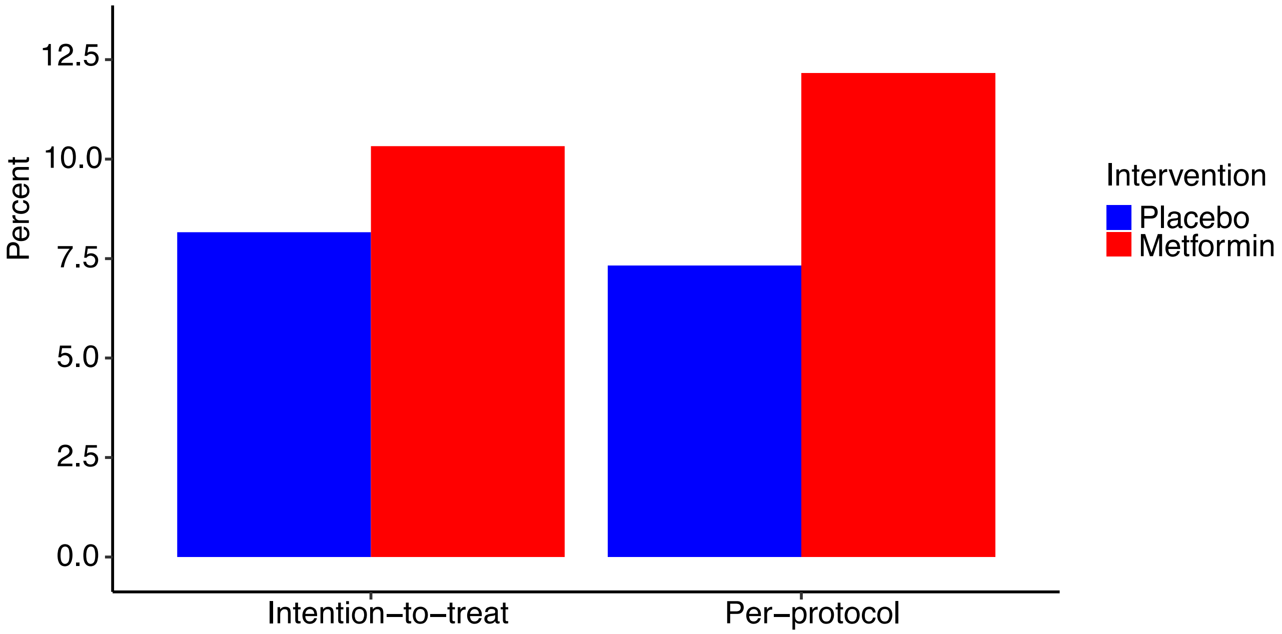

and Per-protocol analysis

SUPPLEMENTAL FIGURE 29. Forest plot showing the results of the logistic regression for the odds of having a surgical site infection according to the ventral hernia working group definition in the Intention-to-treat and Per-protocol analysis

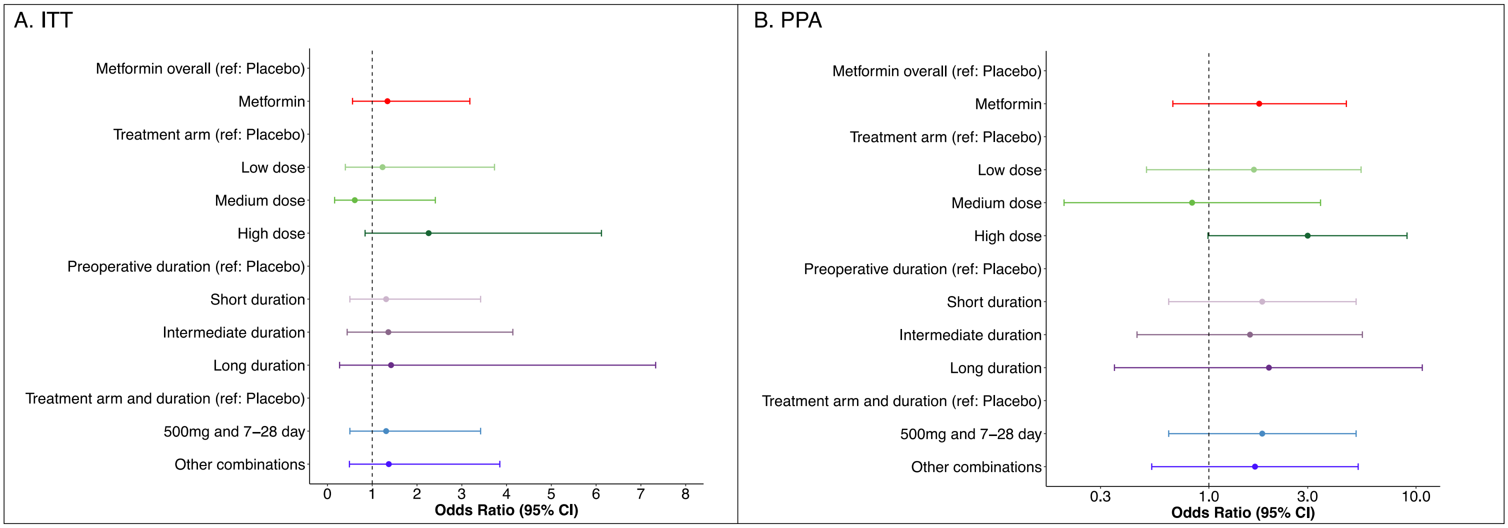

ITT: intention-to-treat, PPA: Per protocol

Panel B: x-axis is in log scale

SUPPLEMENTAL FIGURE 30. Bar graph showing the rate of venous-thromboembolic events by intervention arm in both the Intention-to-treat and Per-protocol analysis

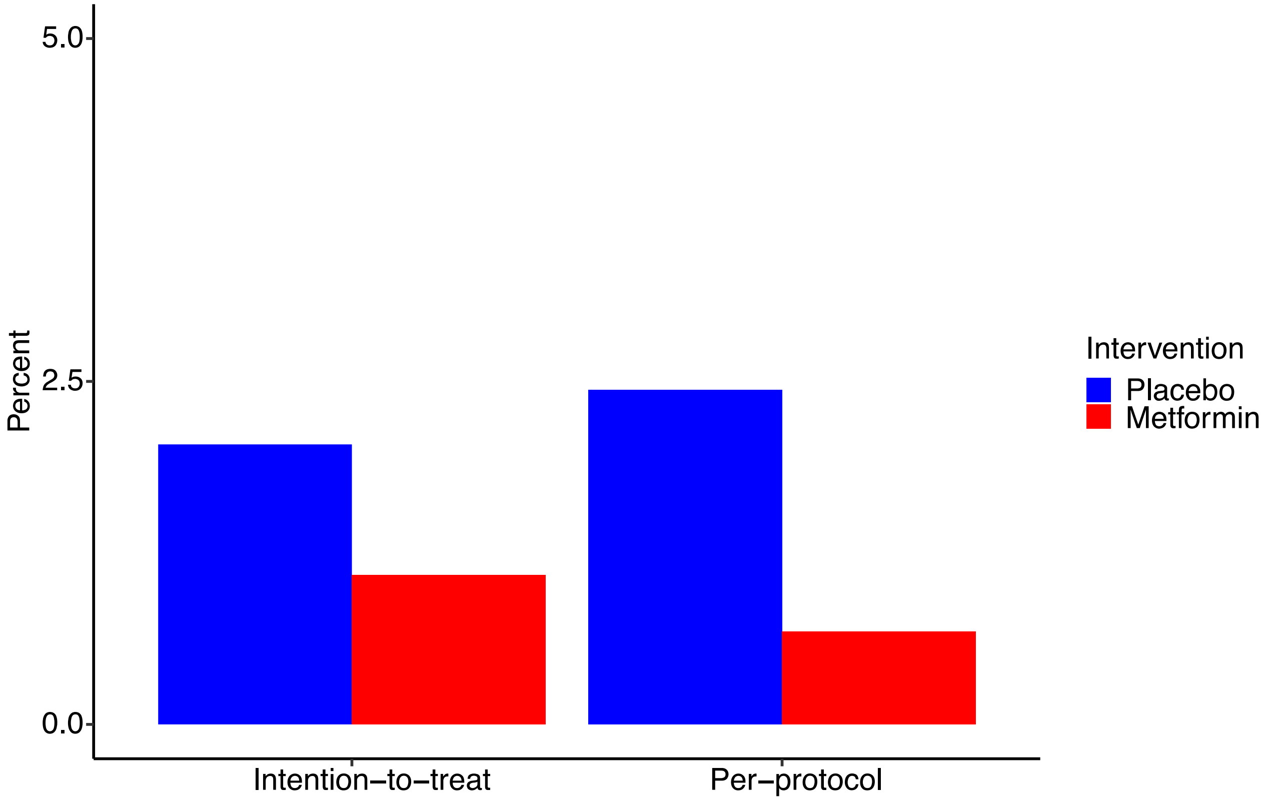

SUPPLEMENTAL FIGURE 31. Forest plot showing the results of the logistic regression for the odds of having a venous-thromboembolic event in the Intention-to-treat and Per-protocol

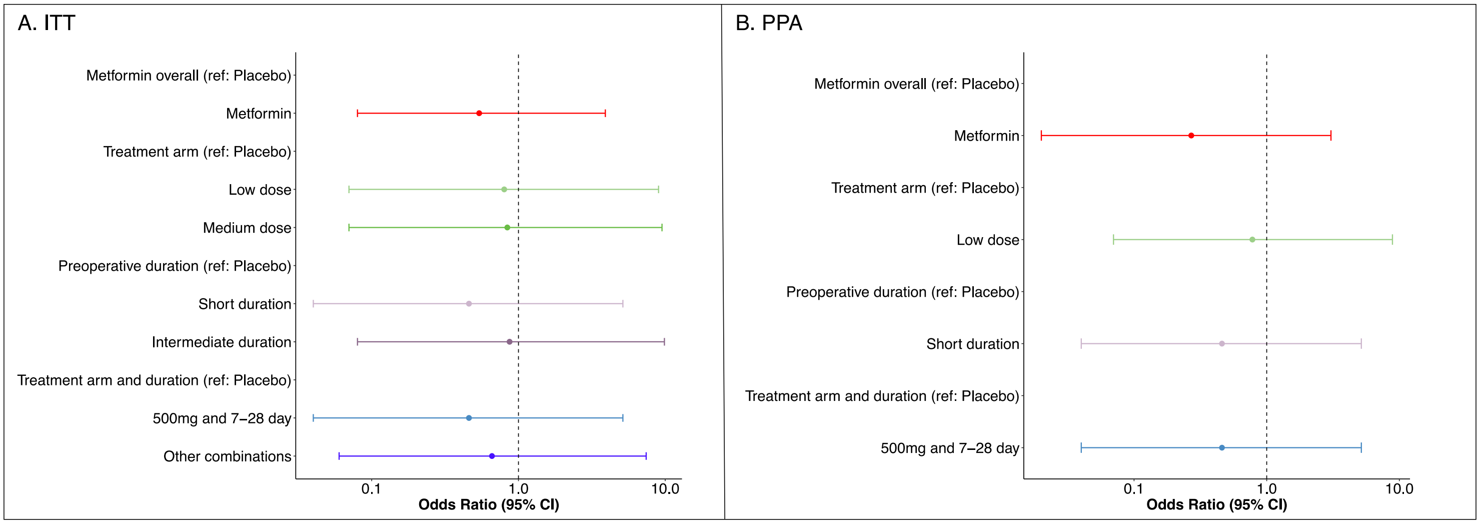

analysis

ITT: intention-to-treat, PPA: Per protocol

x-axis is in log scale. Panel A: long duration group was not included because no events occurred

SUPPLEMENTAL FIGURE 32. Kaplan-Meier curves and forest plot showing the rate of venous-thromboembolic (VTE) event-free survival and results of cox regression for the 90-day VTE events in the Intention-to-treat analysis

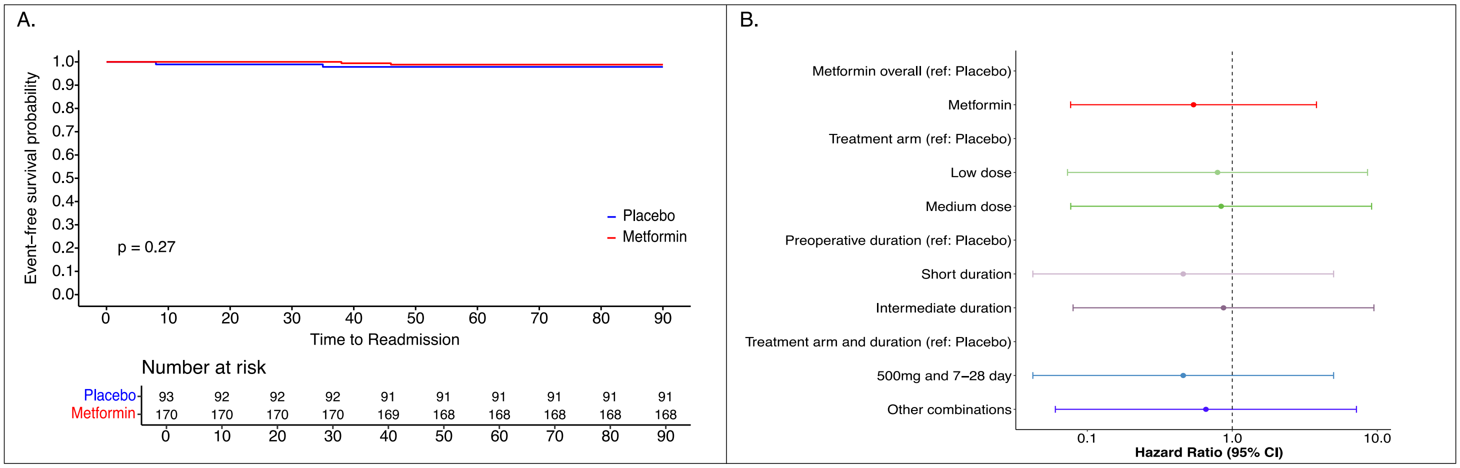

x-axis log scale

Panel B: long duration group was not included because no events occurred

SUPPLEMENTAL FIGURE 33. Kaplan-Meier curves and forest plot showing the rate of venous-thromboembolic (VTE) event-free survival and results of cox regression for the 90-day VTE events in the Per-protocol analysis

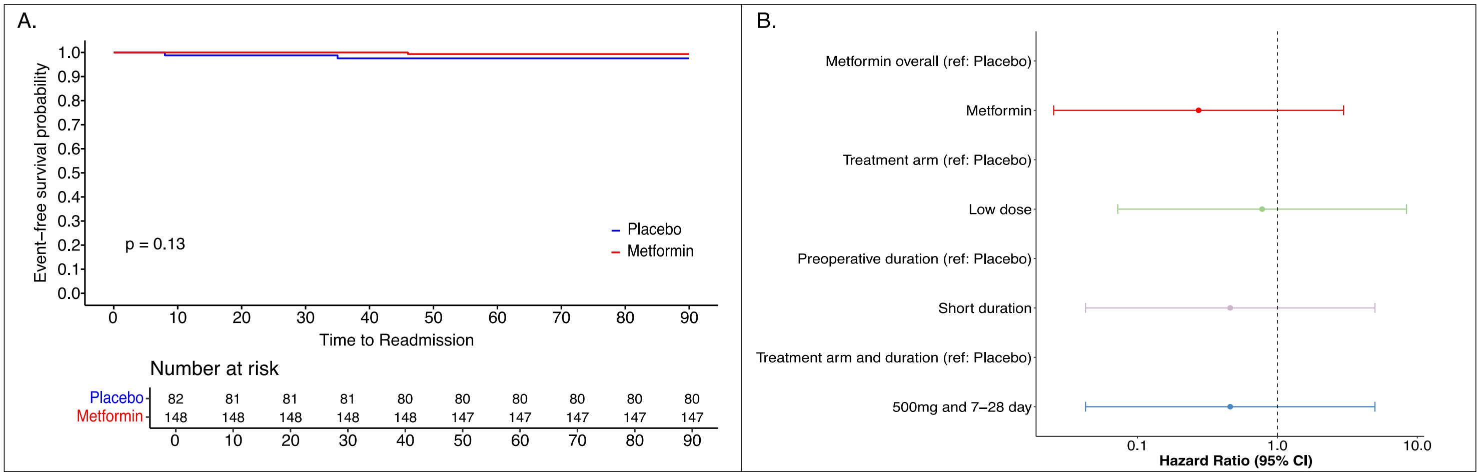

x-axis log scale

Panel A: medium and long duration groups were not included because no events occurred

SUPPLEMENTAL FIGURE 34. Bar graph showing the rate of gastrointestinal disturbances by intervention arm in both the Intention-to-treat and Per-protocol analysis

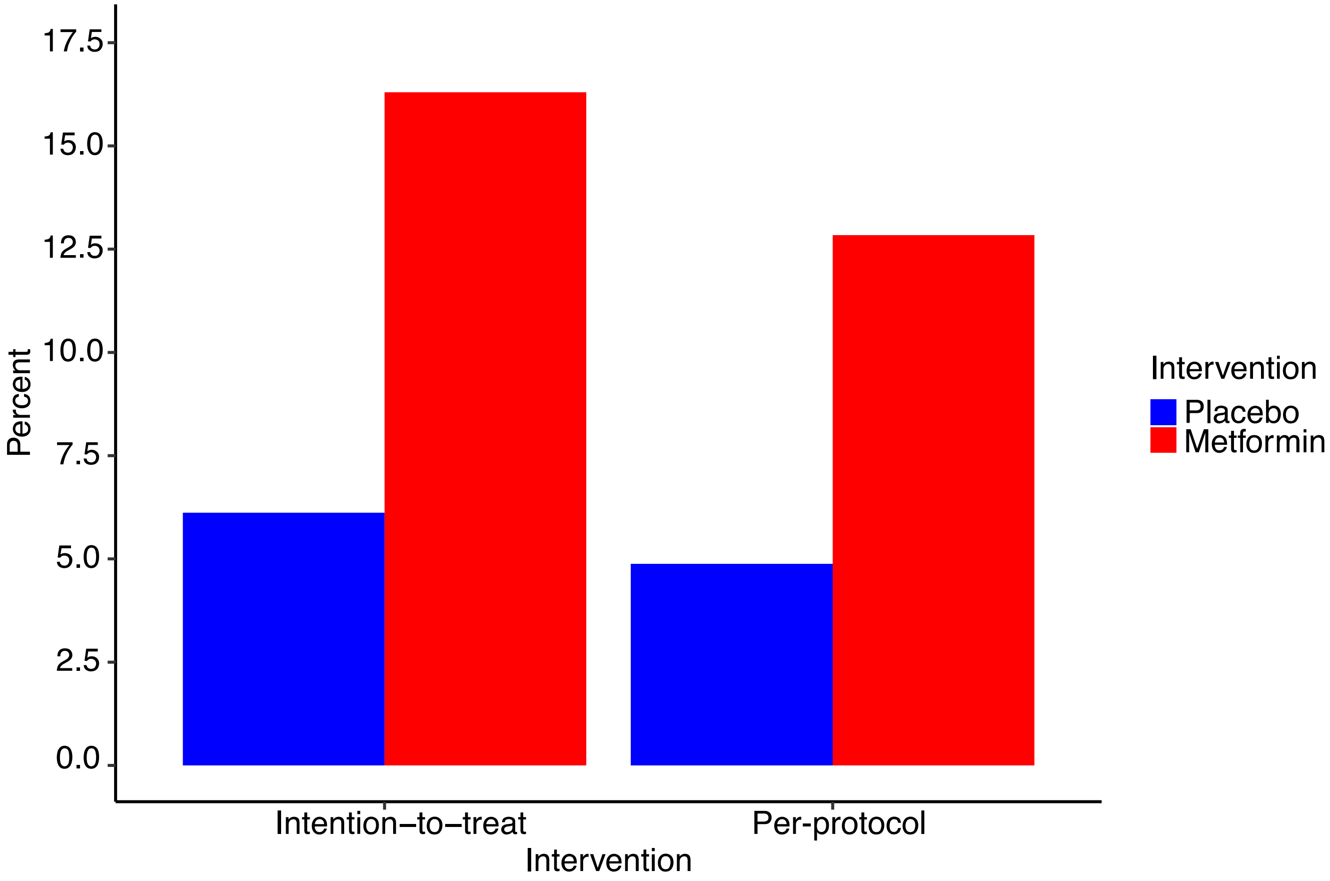

SUPPLEMENTAL FIGURE 35. Forest plot showing the results of the logistic regression for the odds of having a gastrointestinal disturbance event in the Intention-to-treat and Per-protocol

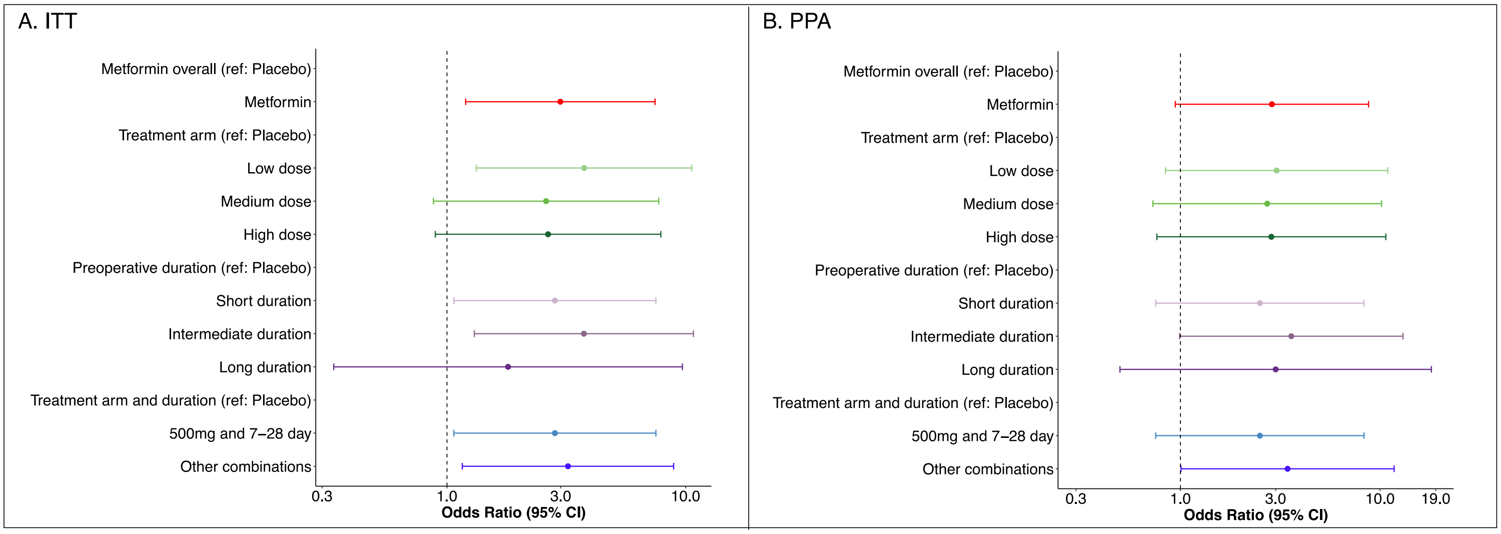

analysis

ITT: intention-to-treat, PPA: Per protocol

x-axis is in log scale

SUPPLEMENTAL FIGURE 36. Rates of serious adverse events in the both treatment groups across the Intention-to-treat and Per-

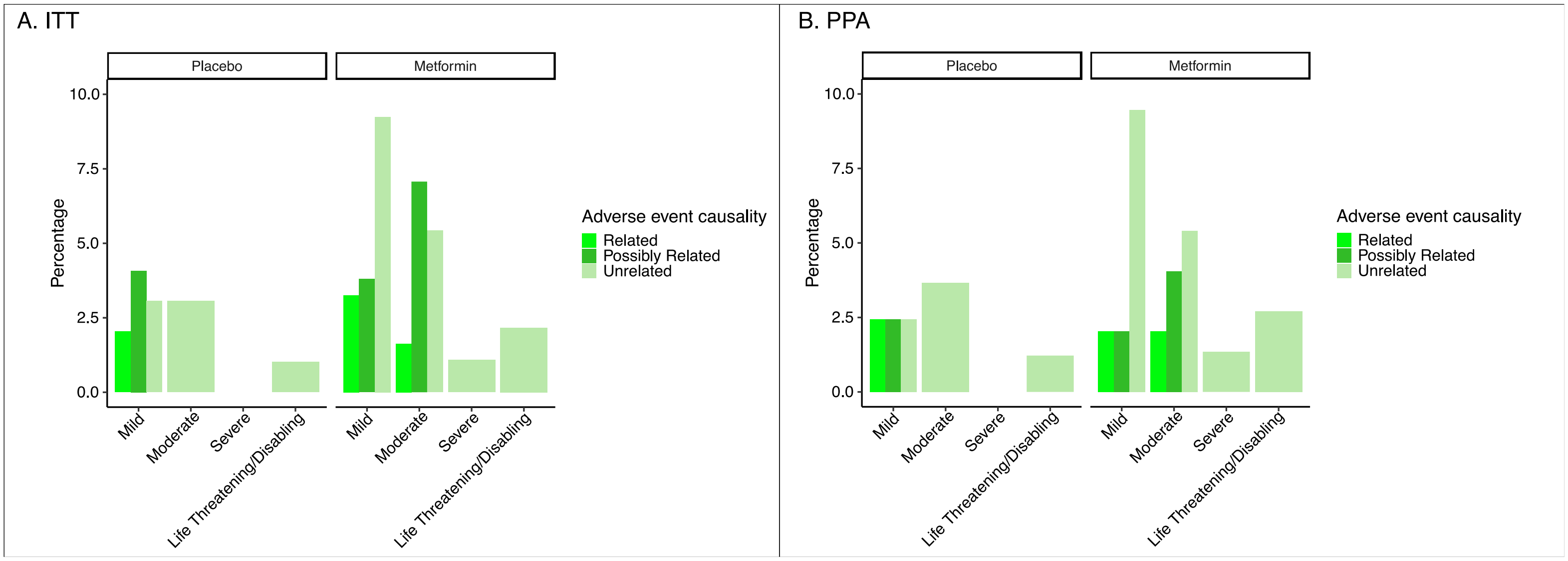

protocol analysis

ITT: intention-to-treat, PPA: Per protocol
